# When Adverse Experiences Influence the Interpretation of Ourselves, Others and the World: A Systematic Review and Meta‐Analysis of Maladaptive Schemas in Victims of Violence

**DOI:** 10.1002/cpp.70114

**Published:** 2025-07-15

**Authors:** Allison Uvelli, Marta Floridi, Giuseppe Agrusti, Anna Chiara Franquillo, Lucia Fiumalbi, Tommaso Micheloni, Andreina Arcuri, Stefania Iazzetta, Andrea Gragnani

**Affiliations:** ^1^ Department of Medical Science, Surgery, and Neurosciences University of Siena Siena Italy; ^2^ School of Cognitive Psychotherapy (SPC) Grosseto Italy; ^3^ School of Cognitive Psychotherapy (SPC) Rome Italy

**Keywords:** adverse experiences, beliefs, early maladaptive schemas, systematic review and meta‐analysis, violence

## Abstract

**Purpose:**

Early maladaptive schemas (EMS) are dysfunctional emotional and cognitive patterns formed in childhood due to negative experiences that hinder basic psychological needs. These schemas shape beliefs about oneself, others and the world, influencing relationships and overall functioning. This study aims to identify common EMS among victims of violence and their associations with specific types of abuse, offering insight into underlying beliefs that may contribute to repeated victimization.

**Methods:**

Following PRISMA guidelines, we conducted a systematic review of observational studies published between May and January 2025, analysing data from online databases. Studies included survivors aged 14 to 60, assessed with the Young Schema Questionnaire. Meta‐analyses with random‐effects models calculated mean effect sizes and correlation coefficients, while meta‐regressions examined the influence of age, gender and country.

**Results:**

A total of 20 studies were included. Survivors exhibited various EMS, including self‐sacrifice, unrelenting standards, abuse, abandonment, dependence and vulnerability to harm. Psychological abuse was strongly linked to emotional deprivation, abuse, social isolation, failure, abandonment, emotional inhibition, vulnerability to harm, defectiveness, dependence and subjugation. Survivors of physical abuse frequently displayed emotional deprivation, social isolation and vulnerability to harm. Among survivors of intimate partner violence, the most prevalent schemas were subjugation, emotional deprivation, abuse and social isolation. Meta‐regressions indicated that age, gender and country influenced certain EMS.

**Conclusions:**

Identifying maladaptive schemas enhances our understanding of survivors' dysfunctional beliefs, which is essential for developing more effective, individualized interventions and preventive strategies.

## Summary

- Early traumatic experiences contribute to the development of maladaptive schemas, negatively impacting self‐perception, relationships and emotional well‐being.
- Different forms of abuse are associated with specific maladaptive schemas, highlighting the need for abuse‐type‐specific interventions.
- Recognizing maladaptive schemas is essential for personalized and effective therapeutic strategies in victims of violence.
- Schema development is influenced by age, gender and cultural context, underscoring the importance of culturally sensitive interventions.
- Early identification and treatment of maladaptive schemas can prevent repeated victimization and promote long‐term psychological health.

## 1 Introduction

### 1.1 Early Maladaptive Schemas (EMS) and Young Schema Questionnaire (YSQ)

EMS are dysfunctional cognitive and emotional structures that develop during early life stages and persist into adulthood (Young 1990, 1999). These schemas originate from adverse childhood experiences (ACEs) and shape an individual's perception of themselves, others and the world (Petrocelli et al. 2001; Delattre et al. 2004; Mason et al. 2005; Bosmans et al. 2010). EMS arise from the frustration of one or more fundamental psychological needs, including secure attachment to others; autonomy, competence and identity; freedom to express needs and emotions; spontaneity and play; and realistic limits and self‐control (Pilkington et al. 2020; Louis et al. 2024; Azadfar et al. 2025). Comprising memories, beliefs, emotions and somatic sensations, EMS serve as core cognitive frameworks that influence an individual's thoughts, behaviours and interpersonal dynamics throughout life (Csukly et al. 2011; Mizara et al. 2012; Zirakbash et al. 2015). They tend to be rigid and resistant to change, significantly impacting emotional well‐being and social functioning (Cockram et al. 2010; Mander et al. 2014; Taylor et al. 2016).

Young and his colleagues (2003) categorized EMS into five overarching domains, each reflecting specific unmet emotional needs or distorted beliefs about oneself, others and the world.

1. **Disconnection and rejection:** This domain develops in individuals from environments characterized by instability, emotional coldness, excessive criticism or social alienation. It includes schemas such as
  • *Abandonment/instability*: the expectation that significant others will be unreliable or unavailable.
  • *Mistrust/abuse*: a belief that others will harm, exploit or deceive.
  • *Emotional deprivation*: a perception of insufficient emotional support or empathy.
  • *Defectiveness/shame*: a deep sense of inferiority and unworthiness.
  • *Social isolation/alienation*: a belief that one is fundamentally different from others and does not belong.
2. **Impaired autonomy and performance:** eshed family environments, leading to a lack of self‐efficacy and personal identity. Key schemas include
  • *Dependence/incompetence*: a belief in one's inability to function independently.
  • *Vulnerability to harm or illness*: an excessive fear of catastrophe or illness.
  • *Enmeshment/undeveloped self*: a lack of personal identity due to excessive emotional involvement with caregivers.
  • *Failure*: a pervasive belief in personal inadequacy and inevitable failure.
3. **Impaired limits:** This domain is associated with permissive or indulgent parenting, resulting in a lack of self‐discipline and empathy. It includes
  • *Entitlement/grandiosity*: a belief in being superior or deserving special treatment.
  • *Insufficient self‐control/self‐discipline*: difficulty maintaining self‐control and perseverance in goal‐directed behaviour.
4. **Other‐directedness:** Individuals with these schemas excessively prioritize others' needs to gain approval or avoid conflict, often at the cost of personal authenticity. These schemas are primarily influenced by the schema in the first domain. It includes
  • *Subjugation*: suppression of personal needs and emotions to avoid disapproval.
  • *Self‐sacrifice*: excessive focus on meeting others' needs at one's own expense.
  • *Approval‐seeking/recognition‐seeking*: an overreliance on external validation for self‐worth.
5. **Over‐vigilance and Inhibition:** These schemas develop in environments with strict, critical or punitive parenting, leading to excessive emotional suppression and rigid control. Key schemas include
  • *Negativity/pessimism*: a focus on potential failures and negative outcomes.
  • *Emotional inhibition*: suppressing emotional expression to avoid disapproval.
  • *Unrelenting standards/hyper‐criticism*: a need for perfection and excessive self‐criticism.
  • *Punitiveness*: a belief that oneself and others deserve harsh punishment for mistakes.

The development of EMS is influenced by sociocultural and demographic factors. Gender differences indicate that women are more likely to develop schemas related to submission, dependence/incompetence and self‐sacrifice, whereas men more commonly exhibit emotional deprivation, emotional inhibition and entitlement/grandiosity—EMS shaped by traditional gender roles (Prince 2009; Irkörücü 2016). Cultural context also plays a crucial role: collectivist societies, which emphasize group belonging, foster schemas related to submission, self‐sacrifice and emotional inhibition, whereas individualistic cultures, which prioritize autonomy, are more associated with unrelenting standards and entitlement/grandiosity (Bakhtiari Moghaddam and Jomehri 2016). Additionally, sexual minorities, due to experiences of discrimination and stigma, are more prone to schemas of submission, self‐sacrifice, approval‐seeking, emotional inhibition and unrelenting standards, which can increase psychological distress (Cardoso et al. 2024). Understanding these sociocultural influences is essential for tailoring interventions to individuals' lived experiences.

Given the profound impact of EMS on psychological well‐being (Hashemipoor et al. 2019; Nicol et al. 2020; Tariq et al. 2021), therapeutic interventions such as Schema Therapy (ST) (Young et al. 2003) have been developed to address these maladaptive patterns (Renner et al. 2012; Hoffart Lunding and Hoffart 2014). ST focuses on identifying and modifying EMS by exploring associated childhood memories, emotions, thoughts and coping styles (Bamber and McMahon 2008; Hosseinifard and Kaviani 2015). Treatment strategies involve reducing the intensity of maladaptive schemas and replacing them with healthier cognitive and behavioural patterns (Young et al. 2003; Khaleghipour et al. 2017; Tenore et al. 2020, 2022; Yakın and Arntz 2023; Boog et al. 2024). A critical tool for assessing EMS is the YSQ (Young 2005; Young and Brown 2005). This self‐report measure evaluates the presence and intensity of maladaptive schemas using a 6‐point Likert scale. The YSQ is available in long (Young 2005) and short versions (Young and Brown 2005), with the long version preferred for clinical use, and the short version commonly utilized in research. Patients complete the questionnaire at home, allowing therapists to focus on interpretation during sessions. Items rated highly (scores of 5 or 6) indicate core schemas, which become the focus of therapeutic intervention. The YSQ provides a structured framework for identifying maladaptive patterns and guiding individualized treatment. Understanding EMS through comprehensive assessment tools such as the YSQ enhances the effectiveness of therapeutic interventions, particularly ST. Given the growing awareness of childhood trauma and its long‐term consequences, integrating EMS‐focused approaches into mental health treatment offers a vital framework for addressing complex psychological challenges.

### 1.2 Dysfunctional Beliefs in Survivors of Abuse: A Cognitive Behavioural Therapy (CBT) and ST Perspective

Individuals who have experienced abuse, whether in childhood or adulthood, often develop dysfunctional beliefs that deeply affect how they perceive themselves, others and the world around them. These beliefs, usually rooted in repeated traumatic experiences, become ingrained cognitive schemas that shape their view of reality, interfering with their ability to form healthy relationships and build a positive future. In CBT and ST, such beliefs are recognized as irrational convictions that, when addressed, help individuals better cope with daily challenges, heal from trauma and enhance their psychological well‐being (Beck 1979; Young 1990; Clark 1996; Kar 2011; Kliethermes et al. 2024). Self‐related beliefs are among the most dysfunctional for abuse survivors, regardless of when the abuse occurred. Traumatic experiences, especially those repeated over time, can lead to a deeply negative self‐image. Individuals who have suffered physical, sexual, psychological, or emotional abuse often internalize a message of inadequacy, believing they do not deserve love or respect. These beliefs can persist into adulthood, manifesting as fragile self‐esteem, difficulty in relationships and a constant tendency towards self‐devaluation (Berber Çelik and Odacı 2019; Ozdemir and Sahin 2020; Melamed et al. 2024). Survivors of abuse may believe statements such as ‘I do not deserve to be treated with respect’ or ‘I cannot trust myself’, beliefs that fuel a cycle of self‐sabotage and keep them trapped in harmful situations (Finkelhor and Browne 1985). Beliefs about others represent another crucial aspect of trauma. Survivors of abuse, whether in childhood or adulthood, tend to develop a distorted view of the world and interpersonal relationships. The trauma they endure may lead to the belief that others are dangerous, malicious or incapable of empathy (Janoff‐Bulman 1979; Mikulincer and Shaver 2016). As Beck (1979) highlighted, childhood trauma affects one's ability to trust others and form relationships based on mutual understanding and respect. Beliefs such as ‘all others are the same’ or ‘people are never sincere’ may lead individuals to avoid seeking social support, which is vital for recovery, and reinforce feelings of isolation (Ehlers and Clark 2000). ST (Young 1994; Farrell et al. 2009) builds on this concept, emphasizing how abuse survivors often develop maladaptive schemas related to mistrust and abandonment, making it difficult to form healthy emotional connections and fostering persistent distrust of others. Recent research shows that trust‐related schemas are crucial in understanding interpersonal difficulties among trauma survivors (Mikulincer and Shaver 2016; Lobbestael et al. 2007). Moreover, beliefs about the world and life in general are deeply influenced by experiences of abuse. Survivors of abuse, especially when trauma is prolonged over time, may develop a fatalistic and pessimistic view of life. The belief that the world is inherently dangerous or unjust can lead to the perception that change is impossible, fostering a sense of learned helplessness. Research by Rowan and Foy (1993) demonstrated how individuals who experienced abuse, particularly in childhood, may develop a view of the world as a ‘hostile place’, where every social interaction is potentially harmful. Survivors of abuse may therefore feel powerless, believing that no action or personal change can truly alter their condition (Seligman 1975; Matusiewicz et al. 2010). This can reinforce the tendency to remain trapped in abusive or violent situations, feeling unable to break the cycle (Seligman 1975). ST also focuses on schemas of defectiveness and inadequacy, which lead survivors to believe they can never achieve a fulfilling life or that they do not deserve happiness or love (Young 1994; Lobbestael et al. 2007). These schemas are reinforced by the distorted belief that the world is a place where suffering is inevitable and change is impossible.

### 1.3 Maladaptive Schemas, Adverse Experiences and Violence

ACEs are negative, stressful and traumatic events that occur during childhood and adolescence (Felitti et al. 1998). They are widely recognized as significant risk factors for various psychological and relational problems (Fabio et al. 2024). ACEs encompass a range of traumatic experiences, including physical abuse, sexual abuse, psychological abuse, physical or emotional neglect and exposure to violence (Anda et al. 2002; Bernstein et al. 2003; Chapman et al. 2004). These experiences can hinder an individual's physical and psychological development. The majority of research has found that all forms of abuse, neglect and witnessed violence increase the likelihood of further victimization and perpetration of violence (Whitfield et al. 2003; Garrido and Taussig 2013; Yan and Karatzias 2016). A recent review (Walker et al. 2017) revealed that in 80 studies, the average prevalence of revictimization was 47.9%, indicating that nearly half of the survivors experienced additional violence in adulthood, and between 40% and 60% of women who are survivors of intimate partner violence (IPV) experience a new assault perpetrated by their current or former partner, or even a future partner (Iverson et al. 2013; Tomkins et al. 2023). But what is the link between childhood and adulthood abuse?

According to Young et al. (2003), schemas develop during childhood and adolescence based on specific experiences and continue to be reinforced throughout adulthood as a result of unmet fundamental human needs. The theory of EMS (Young and Flanagan 1998) suggests that they are present in individuals who were raised in families characterized by instability, violence, lack of affection, excessive demands or social isolation, and who often experienced genuine trauma. This explanation connects the development of maladaptive schemas to childhood and adolescent experiences characterized by adverse events such as ACEs. It suggests that early maladaptive patterns of thinking and behaviour may play a role in the connection between childhood abuse and violence in adulthood (Celsi et al. 2021). In fact, schemas are triggered in adulthood by environmental events that are relevant to the schema, such as conflicts in the individual's interpersonal relationships (Young et al. 2003). In adolescents, a longitudinal study found an association between family violence and the perpetration of dating violence. This indicates that EMS can act as a mechanism through which childhood violence is transmitted intergenerationally (Calvete et al. 2018). Research has shown a link between IPV and EMS also in adult women (Atmaca and Gençöz 2016; Taşkale and Soygüt 2016). These studies suggest that EMS may be a cognitive, emotional and somatic factor linking childhood violence to intimate violence (Borges and Dell'Aglio 2020).

It remains crucial to understand the most common maladaptive schemas in abused individuals to explain the phenomenon of revictimization and why women persist in abusive relationships, especially in cases of domestic violence.

### 1.4 Study Objective

The objectives of this systematic review and meta‐analysis are to

1. identify maladaptive schemas in individuals who have experienced various types of abuse at different stages of life and
2. determine whether the beliefs that develop differ based on the type of abuse endured and how these beliefs may influence subsequent episodes of violence.

Although maladaptive schemas and adverse experiences are both well‐studied topics, no one has yet explored the specific schemas present in victims of violence or how they relate to each type of abuse through meta‐analytic data. This understanding will provide clearer insights into the motivations behind the behaviours of individuals who remain in abusive relationships.

## 2 Materials and Methods

This search protocol was based on the Preferred Reporting Items for Systematic Reviews and Meta‐Analysis (PRISMA) guidelines (Page et al. 2021), according to the PECOS (Population, Exposure, Comparison, Outcome, Study Design) guidelines.

### 2.1 Search Strategy

The research was conducted on the online electronic databases of PubMed, ERIC, Scopus, Web of Science and PsycINFO from May 2024 to January 2025. The databases were selected to contain the highest‐quality empirical studies. The protocol has been registered at the International Prospective Register of Systematic Reviews (PROSPERO; registration number CRD42024572196).

The research question relating to the maladaptive schemas in victims of violence was composed following the PECOS criteria (P—adolescent and adult between 14 to 60 years; E—psychological, physical or sexual violence; C—not victimized people; O—maladaptive schema; S—observational studies, such as cross‐sectional or case–control design) and was: (‘young schema questionnaire’ OR ‘YSQ’ OR ‘maladaptive schema’) AND ([‘abuse’ OR ‘victims’ OR ‘neglect’ OR ‘violence’ OR ‘maltreatment’]). The keywords have been chosen after a preliminary search of the literature thanks to which it was possible to identify the most used and relevant terms. There were no period restrictions on the search to increase the studies' yield, though the language was restricted to studies published in English or Italian. All schemas were considered, while the study design criteria included only studies with high statistical impact. Authors were also contacted via email where there was insufficient data, and references from included studies were manually scanned for further sources as per published recommendations (Higgins and Green 2011; Horsley et al. 2011; Beynon et al. 2013). All studies that assessed maladaptive schema using the YSQ were included in the search to ensure a comprehensive review, following the eligibility criteria.

### 2.2 Eligibility Criteria

The inclusion criteria were as follows:

- Type of participants: adolescents and adults aged 14 to 60, including both survivors and non‐survivors of all kinds of violence.
- Type of studies: Observational studies involve a case group of survivors and a control group of non‐survivors, or at least a single large group of victims.
- Type of instrument: YSQ.
- Study publication language: English or Italian.

The exclusion criteria were as follows:

- qualitative studies, case reports, case studies or case series (due to data that cannot be statistically processed)
- studies not published in English or Italian languages
- studies that evaluate maladaptive schemas without using the YSQ
- studies that only assess the domains and not the specific schemas (all schemas included)
- studies that included participants of different ages than the group of interest
- systematic reviews with or without meta‐analysis
- grey literature (books, conference abstracts, commentaries, dissertations, thesis, editorials, etc.).

### 2.3 Study Selection and Data Extraction

The studies were selected using a three‐stage process. All citations identified from the initial search (articles extracted in May 2024) were imported into Zotero Software. Duplicate citations were removed using the software. After that, two reviewers (L.F. and T.M.) independently scrutinized all the article titles remaining from the original search. Then, the same two reviewers independently analysed all the remaining article abstracts from the second removal. If there was any disagreement, the references were discussed until an agreement was reached, and an independent third reviewer (A.U.) was consulted. For unclear abstracts, the reference was included in the next stage (full‐text screening) to confirm the information in the full text. Full manuscripts were obtained for studies assessed for eligibility, and two reviewers (L.F. and T.M.) carried out an independent full‐text review of all English/Italian language articles. Any disagreements regarding inclusion or exclusion criteria were resolved by consensus or through consultation with an independent third reviewer (A.U.). Subsequently, three reviewers (L.F., T.M. and A.U.) carried out independent data extraction. In cases where extractable data was missing, authors were contacted by email. The summary table was constructed using the authors' names, country, study design, sample characteristics, outcomes and schema results.

### 2.4 Assessment of Study Quality

Quality assessment was conducted using an existing checklist (Moola et al. 2020). Quality was defined as the confidence that bias in the estimation of the effect of abuse on the formation of maladaptive schema outcomes was minimized through appropriate study design methods and analysis. Two independent authors (M.F. and G.A.) assessed the quality of the retrieved articles to identify any potential source of bias using predetermined and validated criteria from The Joanna Briggs Institute appraisal checklist for cross‐sectional and case–control studies (Moola et al. 2020). The appraisal criteria include comparability and appropriateness of cases and controls, description of subjects and setting, reliable and valid measurement of exposure, appropriateness of inclusion criteria, identification of confounding factors and whether strategies were implemented to deal with these factors, valid and reliable assessment of outcomes, exposure time, appropriateness of follow‐up and whether strategies were implemented to deal with incomplete follow‐up, as well as the appropriateness of statistical analysis used. To ensure the quality of a study, certain criteria must be met. For cross‐sectional studies, at least five out of eight criteria should be met, while case–control studies should meet at least six out of 10 criteria. Only studies that meet these standards will be considered high‐quality and included in the results.

### 2.5 Statistical Analysis

A series of meta‐analyses aimed to answer two research questions: What are the most significant schemas in survivors, and which schemas are associated with specific types of abuse? The types of abuse examined, include physical, sexual and psychological abuse during childhood, as well as IPV in adulthood, and we included all the Young schemas encountered in the literature. Statistical analyses were conducted using Comprehensive Meta‐Analysis (CMA, Version 4) and Jamovi software (Version 2.3.0.0). Given that the prevalence of specific schemas may be influenced by various life experiences within the populations studied and considering the diversity of the abuse phenomenon, random‐effects models were employed in this research (Borenstein et al. 2010). The analysis assumes the studies are a random sample from a larger universe of potential studies, and this analysis will be used to infer conclusions about that universe. The mean effect size was determined within a confidence interval that, based on similar studies, could fall anywhere within this range to address the first question. The effect size for the second question was evaluated based on the correlation coefficient between schemas and abuse. In the first study, we used the *z* value to test the null hypothesis that the mean effect size is zero using a criterion alpha of 0.05. The Cochrane's Q index (Hedges 1981) provides a test of the null hypothesis that all studies in the analysis share a common effect size. If all studies shared the same true effect size, the expected value of *Q* would be equal to the degrees of freedom, and we used a criterion alpha of 0.1. We then considered the statistic Higgins's *I* ^2^ (Higgins and Thompson 2002) to determine if the variance in observed effects represents the variance in true effects or sampling error. Lastly, we also considered the tau^2^, the variance of true effect sizes, the tau, the standard deviation of true effect sizes, and the prediction interval, which indicates that the true effect size is expected to fall within this range for 95% of all comparable populations. The rank correlation test and the regression test, using the standard error of the observed outcomes as predictors, are used to check for funnel plot asymmetry. Publication bias was assessed by inspecting a funnel plot and Egger's test (Borenstein et al. 2011). For the second study, we extracted and transformed Pearson's r correlation to Fisher's z and performed all analyses using this transformed value to normalize and stabilize the sampling variance (Borenstein et al. 2011). The results were then converted back to Pearson's r for interpretation. To produce an overall correlation, we conducted some meta‐analyses of effect sizes from all studies reporting correlations between schemas and abuse. Prediction intervals at a 95% confidence level were calculated to estimate the range of correlations. Forest plots were generated, and the amount of heterogeneity (tau^2^) was assessed using the restricted maximum‐likelihood estimator (Viechtbauer 2010). In addition to the estimate of tau^2^, Higgins's *I* ^2^ statistic (Higgins and Thompson 2002) and Cochrane's *Q* index (Hedges 1981) are reported. A tau^2^ > 0, Cochrane's *Q p* value less than 0.1, and an *I* ^2^ value greater than 40% were considered indicators of heterogeneity. In case any amount of heterogeneity is detected, a prediction interval for the true outcome is also provided. Studentized residuals and Cook's distances are used to examine whether studies may be outliers and/or influential in the context of the model. Studies with a studentized residual larger than the 100 × (1–0.05/(2 × *k*))th percentile of a standard normal distribution are considered potential outliers (i.e., using a Bonferroni correction with two‐sided alpha = 0.05 for k studies included in the meta‐analysis). Studies with a Cook's distance larger than the median plus six times the interquartile range of the Cook's distances are considered to be influential. The rank correlation test and the regression test, using the standard error of the observed outcomes as predictors, are used to check for funnel plot asymmetry. Publication bias was assessed by inspecting a funnel plot and Egger's test (Borenstein et al. 2011). An asymmetrical funnel plot may suggest publication bias. At the same time, a non‐statistically significant result for the *t* value of Egger's regression intercept would allow us to rule out the presence of publication bias. Continuous and categorical moderators were analysed using meta‐regression to evaluate their influence on effect sizes in each meta‐analysis. The moderators included were age, gender (women‐only and mixed samples) and country (non‐European and European). Age and country moderators were recoded as binary variables (1 and 2) to ensure comparable subgroups. The significance level was set at *p* < 0.05.

## 3 Results

### 3.1 Literature Identification, Study Characteristics and Quality

**FIGURE 1:**
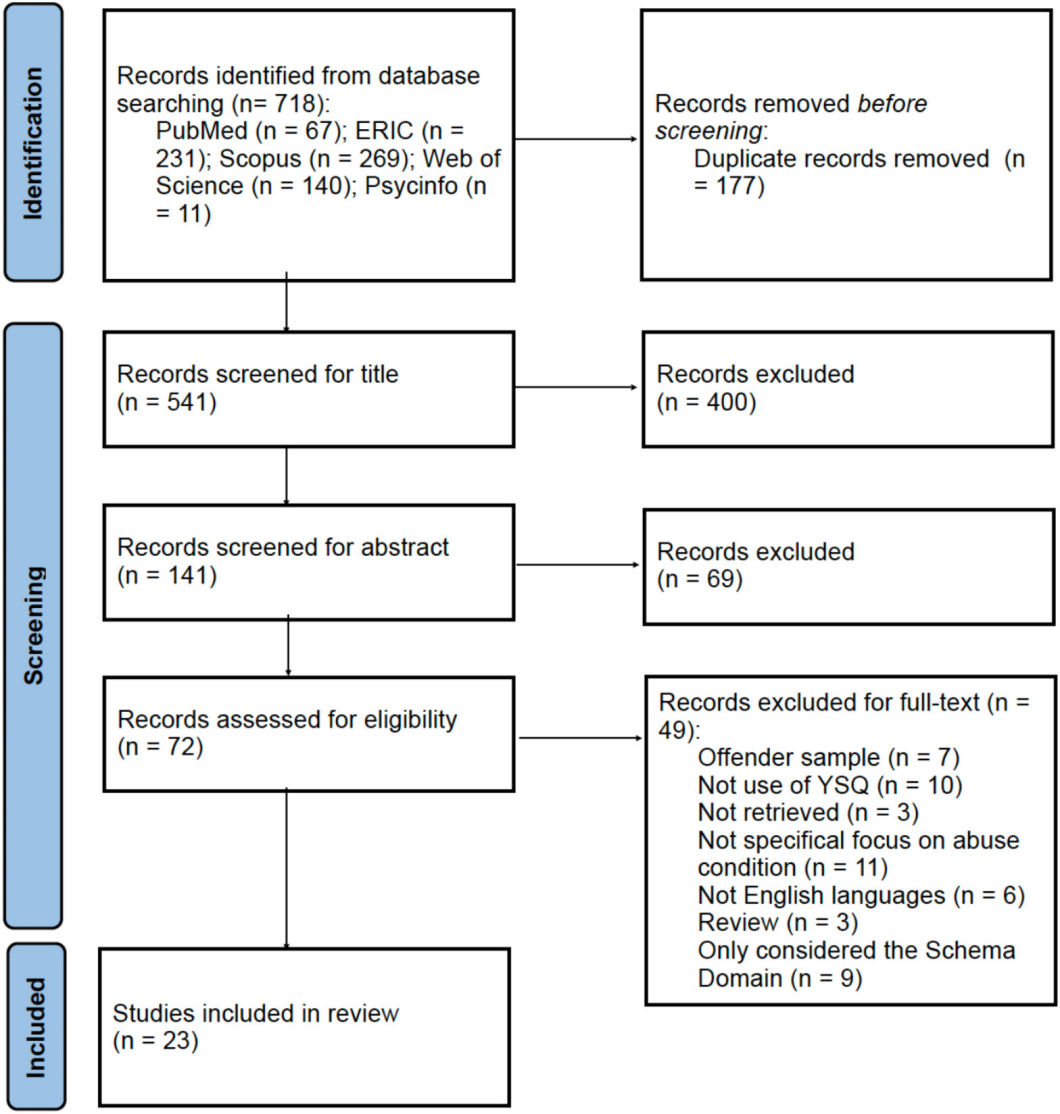
PRISMA flowchart (Page et al. 2021).

The search protocol found 718 publications from online databases. Of these, 177 were identified as duplicate publications and were removed. The remaining 514 studies were screened based on title and abstract criteria, leading to the exclusion of 469 studies. Out of the 72 studies selected for full‐text review, 49 were further excluded for various reasons. These reasons included: seven studies with an offender sample, 10 studies not using the YSQ, three studies that were not retrieved, 11 studies not focused on the abuse condition, six studies written in languages not known by the authors (Portuguese, Russian and Polish), three review studies and nine studies that only considered the domain of the YSQ and not the schemas. Afterward, 23 studies were assessed for quality (Celsi et al. 2021; Pietri and Bonnet 2017; Roemmele and Messman‐Moore 2011; O' Dougherty Wright and Crawford 2009; Mojallal et al. 2021; Boyda et al. 2018; Sójta et al. 2023; Hassija et al. 2017; Obeid et al. 2019; Harding et al. 2011; Khosravi et al. 2011; Estévez et al. 2015; Estévez et al. 2017; Crawford and Wright 2007; Messman‐Moore and Coates 2007; Paim and Falcke 2018; McCarthy and Lumley 2012; Taşkale and Soygüt 2016; Estévez et al. 2024; Fernando et al. 2024; Turner et al. 2005; Muris 2006; Lumley and Harkness 2007). See the flow diagram in Figure 1. Three of these 23 studies potentially eligible for inclusion were excluded due to low quality (Boyda et al. 2018; Khosravi et al. 2011; Paim and Falcke 2018), leaving 20 final articles. Of the 16 cross‐sectional studies, four met five criteria, four met six criteria, five met seven criteria and three met eight criteria. The four case–control studies met six, seven and eight out of 10 criteria, respectively. No other articles were excluded based on the quality standards.

The years of the study range from 2005 to 2024, 16 studies are cross‐sectional, and four studies are case–control. Studies from the USA are 35%, 15% are from Spain, 10% are from Canada, 25% are from other countries in Europe and 15% are from other extra‐Europe countries. The sample size ranges from 46 to 707, both males and females, cases and controls, its age ranges from 13 to 49, and all the abuse types are represented, either in childhood or IPV. Twelve articles focus only on childhood abuse (Roemmele and Messman‐Moore 2011; O' Dougherty Wright and Crawford 2009; Mojallal et al. 2021; Harding et al. 2011; Estévez et al. 2015; Estévez et al. 2017; Messman‐Moore and Coates 2007; McCarthy and Lumley 2012; Fernando et al. 2024; Turner et al. 2005; Muris 2006; Lumley and Harkness 2007), five articles focus only on IPV (Sójta et al. 2023; Hassija et al. 2017; Obeid et al. 2019; Taşkale and Soygüt 2016; Estévez et al. 2024) and three articles focus on both (Celsi et al. 2021; Pietri and Bonnet 2017; Crawford and Wright 2007). Table 1 summarizes the characteristics of the included studies, and qualitative results are present in Supporting Information.

**TABLE 1:** Characteristics of the included studies.

Authors	Study design	Sample	Abuse/ACE	EMS linked with the abuse
Celsi et al. 2021 Italy	Cross‐sectional	134 non‐cohabiting couples (67 F, 21.89 y/o; 67 M, 23.49 y/o)	Childhood abuse: psychological, neglect and witnessed violence IPV	Childhood abuse: abandonment and instability IPV: emotional deprivation
Pietri and Bonnet 2017 France	Case–control	80 F (40 cases, 40 controls) 34.5 y/o	Childhood abuse: witnessed violence IPV	Self‐sacrifice Mistrust and abuse Subjugation Emotional deprivation
Roemmele and Messman‐Moore 2011 USA	Cross‐sectional	653 F 18.77 y/o	Childhood abuse: sexual, physical and psychological	Sexual and physical abuse: mistrust and abuse, emotional deprivation, abandonment and instability Psychological abuse: emotional deprivation, defectiveness and shame, mistrust and abuse
O' Dougherty Wright and Crawford 2009 USA	Cross‐sectional	301 (158 F, 143 M) 20.37 y/o	Childhood abuse: sexual, physical and psychological and emotional neglect	Vulnerability to harm Self‐sacrifice Defectiveness and shame
Mojallal et al. 2021 USA	Cross‐sectional	415 (304 F, 111 M) 19.31 y/o	Childhood abuse: sexual, physical and neglect	Social isolation Failure Dependence and incompetence Emotional inhibition
Sójta et al. 2023 Poland	Case–control	96 F (48 cases, 32.91 y/o; 48 controls, 34.27 y/o)	IPV	Self‐sacrifice Mistrust and abuse Negativity and pessimism Abandonment and instability
Hassija et al. 2017 USA	Cross‐sectional	305 F 24.33 y/o	IPV	Subjugation Mistrust and abuse Self‐sacrifice
Obeid et al. 2019 Lebanon	Cross‐sectional	707 (361 F, 328 M) 25.20 y/o	IPV	Emotional deprivation Mistrust and abuse Defectiveness and shame
Harding et al. 2011 USA	Cross‐sectional	177 F (127 cases, 50 controls) 19.0 y/o	Childhood abuse: sexual, physical, psychological and emotional neglect	Sexual abuse: mistrust and abuse, emotional deprivation and self‐sacrifice Physical abuse and emotional neglect: emotional deprivation, mistrust and abuse, abandonment and instability Psychological abuse: emotional deprivation, mistrust and abuse and dependence
Estévez et al. 2015 Spain	Cross‐sectional	122 (108 F, 14 M) 34.68 y/o	Childhood abuse: psychological and emotional neglect	Emotional deprivation Failure Mistrust and abuse
Estévez et al. 2017 Spain	Cross‐sectional	75 F 34.49 y/o	Childhood abuse: physical, sexual, psychological, physical and emotional neglect	Sexual abuse: failure, mistrust and abuse and self‐sacrifice Physical abuse: vulnerability to harm Psychological abuse: failure, vulnerability to harm and self‐sacrifice Neglect: emotional deprivation and emotional inhibition
Crawford and Wright 2007 USA	Cross‐sectional	301 (158 F, 143 M) 20.37 y/o	Childhood abuse: physical, sexual, psychological, physical and emotional neglect IPV	Mistrust and abuse Self‐sacrifice Emotional inhibition
Messman‐Moore and Coates 2007 USA	Cross‐sectional	382 F 19.3 y/o	Childhood abuse: psychological	Abandonment and instability Mistrust and abuse Defectiveness and shame
McCarthy and Lumley 2012 Canada	Cross‐sectional	97 (80 F, 16 M) 18.76 y/o	Childhood abuse: psychological	Unrelenting standards Self‐sacrifice
Taşkale and Soygüt 2016 Turkey	Case–control	157 F (79 cases, 31.68 y/o; 78 controls, 41.50 y/o)	IPV	Unrelenting standards
Estévez et al. 2024 Spain	Case–control	403 F (61 cases, 48.43 y/o); 342 controls, 26.91 y/o	IPV	Subjugation Emotional deprivation Mistrust and abuse Social isolation
Fernando et al. 2024 Australia	Cross‐sectional	231 (178 F, 52 M) 23.7 y/o	Childhood abuse: sexual, physical and psychological abuse, emotional and physical neglect	Emotional deprivation Social isolation Defectiveness and shame Mistrust and abuse
Turner et al. 2005 UK	Cross‐sectional	46 F 17.7 y/o	Childhood abuse: psychological	Emotional deprivation Abandonment and instability Subjugation
Muris 2006 The Netherlands	Cross‐sectional	173 (86 F, 87 M) 13.32 y/o	Childhood abuse: psychological	Self‐sacrifice Unrelating standards Emotional inhibition
Lumley and Harkness 2007 Canada	Cross‐sectional	76 (52 F, 24 M) 15.8 y/o	Childhood abuse: sexual, physical and psychological	Sexual abuse: vulnerability to harm, dependence and failure Physical abuse: vulnerability to harm, failure and emotional deprivation Psychological abuse: subjugation, vulnerability to harm and dependence

### 3.2 Meta‐Analysis Results

A series of meta‐analyses were conducted to address the research questions. To identify which EMS are most prevalent among survivors, meta‐analyses were performed using the mean scores and standard deviations for each schema from the YSQ. Additionally, to determine which EMS are most associated with specific types of abuse, meta‐analyses were conducted using the correlations between the schemas and the abuse. Only articles that provided the necessary data for these statistical analyses were included. Authors of articles lacking data were contacted via email, and those who offered additional material were included in the analysis. Other studies were used solely for qualitative review. In addition to the first study, moderation analyses were conducted to understand the potential influence of age, gender and country.

#### 3.2.1 What Are the Schemas Commonly Found in Survivors?

The studies that provided the necessary data and were included in meta‐analyses are as follows: Harding et al. (2011), Hassija et al. (2017), McCarthy and Lumley (2012), Sójta et al. (2023), Taskale and Soygut (2016), Celsi et al. (2021), Obeid et al. (2019), Estévez et al. (2015, 2017, 2024), Fernando et al. (2024), Turner et al. (2005) and the schema tested included self‐sacrifice, unrelating standards, abuse, abandonment, dependence, vulnerability to harm, social isolation, emotional deprivation and inhibition, failure, subjugation, defectiveness and enmeshment (schemas for which there were data to conduct statistical analysis). Despite all the analysed schemas obtaining significant results, a higher mean score indicates that the schema is more prevalent. To enhance comprehension, we designated scores above 13 as significant. The most prevalent EMS in survivors, in descending order, are self‐sacrifice, unrelating standards, abuse, abandonment, dependence and vulnerability to harm. Each EMS has a different mean effect size, displayed in Table 2 in descending order. In all cases, we can reject the null hypothesis that the mean effect size is zero (*Z*, *p*) and the null hypothesis that the true effect size is the same in all studies (*Q*, df, *p*). The *I* ^2^ statistic ranges between 93% and 99%, which tells that 93%–99% of the variance in observed effects reflects variance in true effects rather than sampling error, and together with tau^2^ indicates heterogeneity. Suppose we assume that the true effects are normally distributed (in raw units). In that case, we can estimate different prediction intervals, and the true effect size in 95% of all comparable populations falls in those intervals. The great heterogeneity explains the wide prediction interval present in some EMS. The rank correlation and the regression test do not indicate any funnel plot asymmetry in all schemas (Begg and Mazumdar, *p*; Egger's regression, *p*). The Trim and Fill method reports adding one or two studies for emotional deprivation, emotional inhibition and failure schemas to contrast the publication bias. Still, such studies were not found in the literature (see the significant funnel plots in the Supporting Information).

**TABLE 2:** Random effects meta‐analysis results of the mean scores of EMS in survivors*.

EMS	Mean	Lower limit–upper limit	*Z* (*p*)	*Q* (df; *p*)	*I* ^2^	Standard error	Variance	Tau^2^ (tau)	Prediction interval	Begg and Mazumdar (*p*)	Egger's regression (*p*)	Trim and fill
*Self‐sacrifice*	18.22	16.81‐ 19.63	25.36 (< 0.001)	249.24 (8; < 0.001)	97%	0.71	0.51	4.36 (2.09)	13.02–23.45	0.02 (0.45)	0.26 (0.39)	0
*Unrelating standard*	15.83	12.44–19.22	9.15 (< 0.001)	705.49 (6; < 0.001)	99%	1.73	2.99	20.59 (4.53)	3.34–28.31	−0.28 (0.18)	0.87 (0.20)	0
*Abuse*	13.86	12.40–15.33	18.56 (< 0.001)	239.45 (8; < 0.001)	97%	0.74	0.55	4.72 (2.27)	8.43–19.30	0.08 (0.37)	0.87 (0.20)	0
*Abandonment*	13.85	12.11–15.59	15.57 (< 0.001)	563.25 (10; < 0.001)	98%	0.88	0.79	8.36 (2.89)	7.10–20.69	0.29 (0.10)	1.23 (0.12)	0
*Dependence*	13.19	10.50–15.89	9.60 (< 0.001)	1042.37 (7; < 0.001)	99%	1.37	1.89	14.77 (3.84)	3.20–23.18	0.32 (0.13)	0.84 (0.21)	0
*Vulnerability to harm*	13.03	10.90–15.17	11.94 (< 0.001)	327.99 (6; < 0.001)	98%	1.09	1.19	8.08 (2.83)	5.24–20.83	0.00 (0.50)	0.02 (0.49)	0
Social isolation	12.99	10.94–15.03	12.43 (< 0.001)	660.77 (9; < 0.00\)	99%	1.04	1.09	10.55 (3.25)	5.11–20.86	0.26 (0.14)	1.11 (0.14)	0
Emotional deprivation	12.85	11.07–14.69	13.65 (< 0.001)	517.43 (9; < 0.001)	98%	0.94	0.88	8.55 (2.92)	5.76–19.93	0.13 (0.29)	1.05 (0.16)	2
Emotional inhibition	12.79	11.61–13.96	21.35 (< 0.001)	96.99 (7; < 0.001)	93%	0.59	0.35	2.54 (1.59)	8.61–16.96	0.17 (0.26)	0.75 (0.24)	1
Failure	11.76	10.47–13.05	17.87 (< 0.001)	129.91 (7; < 0.001)	95%	0.65	0.43	3.11 (1.76)	7.15–16.36	0.00 (0.50)	0.77 (0.23)	2
Subjugation	11.16	9.92–12.40	17.66 (< 0.001)	151.02 (7; < 0.001)	95%	0.63	0.40	2.95 (1.71)	6.68–15.64	0.03 (0.45)	0.13 (0.44)	0
Defectiveness	11.13	10.09–12.17	20.93 (< 0.001)	111.29 (8; < 0.001)	93%	0.53	0.28	2.20 (1.48)	7.40–14.86	0.08 (0.37)	0.06 (0.47)	0
Enmeshment	10.15	7.46–12.85	7.38 (< 0.001)	445.13 (5; < 0.001)	99%	1.37	1.89	11.12 (3.33)	0.14–20.17	−0.13 (0.35)	1.26 (0.13)	0

* *Italics* EMS are those taken into consideration.

##### 3.2.1.1 Moderation Analysis: The Effect of Age, Gender and Culture on Survivors' Schemas

The moderation analysis indicated that age, gender and country did not have significant moderating effects on most of the EMS. However, on the self‐sacrifice schema, the age variable influences the final effect (*p* = 0.01). In fact, as age increases, the score for this schema also rises. In the unrelating standards schema, age and country have significant influences (*p* = 0.004; 0.002). As age increases, this schema score decreases, with countries outside Europe showing higher scores than those within Europe. Among the two variables, age has the greatest influence (−1.43, *p* = 0.15). On the dependence schema, the age and country variables influence the final effect (*p* = 0.002; 0.04). As age increases, the score for this schema also rises, with European countries showing higher scores than those outside Europe. Among the two variables, age has the greatest influence (1.76, *p* = 0.07). In the vulnerability to harm schema, age has a significant influence (*p* = 0.007); in fact, as age increases, the score also rises. Lastly, on the emotional deprivation schema, the age, gender and country variables influence the final effect (*p* = 0.003; 0.03; 0.01). As age increases, the score for this schema also rises, with the women samples and European countries showing higher scores than mixed samples and extra‐European countries. Among the three variables, age has the greatest influence (2.00, *p* = 0.04), then gender (−1.57, *p* = 0.11) and finally the country (−0.38, *p* = 0.70) (see the moderation analysis table and significant scatter plots in the Supporting Information).

#### 3.2.2 Psychological Abuse

The studies that provided the necessary data and were included in these meta‐analyses are as follows: Messman‐Moore and Coates (2007), Roemmele and Messman‐Moore (2011), Harding et al. (2011), McCarthy and Lumley (2012), Estévez et al. (2015), Estévez et al. (2017), Harding et al. (2011), Mojallal et al. (2021), Turner et al. (2005), Muris (2006), Lumley and Harkness (2007) and Celsi et al. (2021). The schemas tested included abuse, emotional deprivation, dependence, failure, emotional inhibition, social isolation, vulnerability to harm, defectiveness, abandonment, subjugation, self‐sacrifice, enmeshment and unrelated standards (schemas for which there were data to conduct statistical analysis). However, only those with a correlation greater than 0.30 were considered relevant and are indicated in *italics* in Table 3. The EMS most associated with psychological abuse, in descending order, are emotional deprivation, abuse, social isolation, failure, abandonment, emotional inhibition, vulnerability to harm, defectiveness, dependence and subjugation. These had a correlation coefficient between 0.45 and 0.30 (95% CI, 0.18–0.52; *p* < 0.002), with the majority of estimates being positive (100%). Therefore, the average outcome differed significantly from zero (*t* (*p*)). According to the *Q* test in vulnerability to harm, social isolation, failure and defectiveness schemas, there was no significant amount of heterogeneity in the true outcomes; in abandonment, emotional deprivation, dependence and subjugation schemas, the *Q* test for heterogeneity was not significant, but some heterogeneity may still be present in the true outcomes; and in abuse and emotional inhibition schemas, the true outcomes appear to be heterogeneous (*Q* [df; *p*], tau^2^, *I* ^2^). The 95% prediction interval for the true outcomes ranges between 0.008 to 0.76. Hence, even though some studies may have some heterogeneity, the true outcomes of the studies are generally in the same direction as the estimated average outcome. An examination of the studentized residuals revealed that none of the studies had a value larger than ±2.63–2.77 for all the schemas; hence, there was no indication of outliers in the context of these models. The rank correlation and the regression test do not indicate any funnel plot asymmetry in all significant schemas (Begg and Mazumdar, *p*; Egger's regression, *p*). The Trim and Fill method reports adding one study for social isolation and subjugation schemas to contrast the publication bias. Still, such studies were not found in the literature (see the significant forest plots and funnel plots in the Supporting Information).

**TABLE 3:** The correlation coefficient of schemas in survivors of psychological abuse during childhood*.

EMS	Correlation coefficient	Lower limit–upper limit	*t* (*p*)	*Q* (df, *p*)	Tau^2^ (tau)	*I* ^2^	Prediction interval	Begg and Mazumdar (*p*)	Egger's regression (*p*)	Trim and fill
*Emotional deprivation*	0.45	0.40–0.50	20.3 (< 0.001)	14.78 (8; 0.06)	0 (0.001)	0%	0.39–0.49	−0.14 (0.60)	−0.19 (0.85)	0
*Abuse*	0.40	0.29–0.51	8.38 (< 0.001)	22.15 (8; 0.005)	0.01 (0.11)	68.79%	0.11–0.68	0.00 (1.00)	0.22 (0.83)	0
*Social isolation*	0.39	0.31–0.48	10.5 (< 0.001)	9.13 (7; 0.24)	0.003 (0.06)	33.20%	0.22–0.56	−0.25 (0.38)	−2.24 (0.06)	1
*Failure*	0.36	0.27–0.46	8.96 (< 0.001)	11.10 (7; 0.13)	0.005 (0.07)	41.20%	0.16–0.55	0.03 (0.90)	−0.79 (0.45)	0
*Abandonment*	0.36	0.28–0.44	10.7 (< 0.001)	14.40 (8; 0.07)	0.004 (0.06)	44.04%	0.19–0.52	−0.05 (0.91)	−0.34 (0.74)	0
*Emotional inhibition*	0.35	0.18–0.52	5.08 (0.002)	21.52 (6; 0.001)	0.02 (0.15)	75.81%	0.06–0.76	−0.14 (0.77)	−0.97 (0.37)	0
*Vulnerability to harm*	0.35	0.26–0.44	9.83 (< 0.001)	3.91 (6; 0.68)	0 (0)	0%	0.26–0.44	−0.09 (0.76)	0.11 (0.91)	0
*Defectiveness*	0.33	0.26–0.39	12 (< 0.001)	9.64 (8; 0.29)	0.001 (0.03)	16.19%	0.22–0.42	−0.25 (0.34)	−0.47 (0.65)	0
*Dependence*	0.32	0.21–0.44	6.73 (< 0.001)	11.45 (6; 0.07)	0.007 (0.08)	49.85%	0.08–0.56	−0.33 (0.38)	−0.78 (0.46)	0
*Subjugation*	0.30	0.23–0.37	10.8 (< 0.001)	10.50 (6; 0.10)	0 (0.002)	0.04%	0.23–0.37	0.04 (1.00)	0.32 (0.76)	1
Self‐sacrifice	0.23	0.12–0.33	5.64 (0.002)	2.37 (5; 0.79)	0 (0)	0%	0.12–0.33	−0.33 (0.46)	−0.66 (0.54)	0
Enmeshment	0.17	0.04–0.29	3.41 (0.01)	6.14 (5; 0.29)	0.004 (0.06)	28.92%	0.04–0.37	−0.06 (1.00)	−1.45 (0.22)	3
Unrelating standards	0.17	0.09–0.24	5.13 (0.002)	9.04 (6; 0.17)	0 (0.01)	4.5%	0.07–0.25	0.14 (0.77)	0.61 (0.56)	0

* *Italics* EMS are those considered relevant according to the correlation coefficient.

#### 3.2.3 Physical Abuse

The studies that provided the necessary data and were included in these meta‐analyses are as follows: Roemmele and Messman‐Moore (2011), Estévez et al. (2017), Mojallal et al. (2021), Celsi et al. (2021), Fernando et al. (2024), Lumley and Harkness (2007) and Harding et al. (2011). The schema tested encompasses emotional deprivation, social isolation, vulnerability to harm, dependence, failure, abuse, emotional inhibition, defectiveness, subjugation, abandonment, unrelenting standards, enmeshment and self‐sacrifice (schemas for which there were data to conduct statistical analysis). However, only those with a correlation greater than 0.30 were considered relevant and are indicated in *italics* in Table 4. The EMS most associated with physical abuse are emotional deprivation, social isolation and vulnerability to harm. These had a correlation coefficient of 0.42 (95% CI, 0.11–0.72; *p* = 0.01), 0.31 (95% CI, 0.11–0.50; *p* = 0.01) and 0.30 (95% CI, 0.06–0.54), respectively, with the majority of estimates being positive (100%). Therefore, the average outcome differed significantly from zero (*t* (*p*)). According to the *Q* test, the true outcomes appear to be heterogeneous for the emotional deprivation and social isolation schema; instead, there was no significant amount of heterogeneity in the true outcomes for vulnerability to harm (*Q* (df; *p*), tau^2^, *I* ^2^). The 95% prediction interval for the true outcomes is given by 0.11 to 1.19 for the first schema, 0.11 to 0.72 for the second and 0.06 to 0.54 for the third. An examination of the studentized residuals revealed that one study had a value larger than ±2.57–2.63 and may be a potential outlier in the context of this model for emotional deprivation and social isolation schemas, but not for the vulnerability to harm. Deleting the potential outlier did not improve the index due to the small number of studies, so we cannot remove it. The rank correlation and the regression test do not indicate any funnel plot asymmetry in all significant schemas (Begg and Mazumdar, *p*; Egger's regression, *p*). The trim and fill method reports adding one or two studies to contrast the publication bias. Still, such studies were not found in the literature (see the significant forest plots and funnel plots in the Supporting Information).

**TABLE 4:** The correlation coefficient of the schema for victims of physical abuse during childhood*.

EMS	Correlation coefficient	Lower limit–upper limit	*t* (*p*)	*Q* (df, *p*)	Tau^2^ (tau)	*I* ^2^	Prediction interval	Begg and Mazumdar (*p*)	Egger's regression (*p*)	Trim and fill
*Emotional deprivation*	0.42	0.11–0.72	3.54 (0.01)	99.55 (5; < 0.001)	0.07 (0.27)	94.2%	0.11–1.19	−0.33 (0.46)	−0.81 (0.45)	2
*Social isolation*	0.31	0.11–0.50	4.34 (0.01)	19.66 (4; < 0.001)	0.01 (0.13)	75.43%	0.11–0.72	−0.60 (0.23)	−1.99 (0.14)	1
*Vulnerability to harm*	0.30	0.06–0.54	5.43 (0.03)	0.77 (2; 0.6)	0 (0)	0%	0.06–0.54	0.33 (1.00)	0.73 (0.59)	2
Abuse	0.28	0.18–0.38	7.75 (0.001)	5.07 (4; 0.28)	0.001 (0.03)	22.41%	0.13–0.42	−0.40 (0.48)	−0.48 (0.65)	0
Failure	0.28	−0.02 to 0.59	3.00 (0.05)	19.78 (3; < 0.001)	0.02 (0.16)	80.36%	−0.32 to 0.89	−0.33 (0.75)	−0.77 (0.52)	0
Dependence	0.27	0.08–0.47	4.45 (0.02)	6.56 (3; 0.08)	0.007 (0.08)	52.91%	0.08–0.61	−0.33 (0.75)	−1.77 (0.21)	2
Emotional inhibition	0.25	−0.03 to 0.53	2.86 (0.06)	13.76 (3; 0.003)	0.02 (0.14)	76.75%	−0.30 to 0.80	−0.66 (0.33)	−3.44 (0.07)	1
Abandonment	0.22	0.15–0.29	8.48 (< 0.00)	2.24 (5; 0.81)	0 (0)	0%	0.15–0.29	−0.46 (0.27)	−0.72 (0.51)	2
Defectiveness	0.22	0.12–0.33	5.74 (0.005)	5.16 (4; 0.27)	0.002 (0.04)	30.71%	0.05–0.39	0.20 (0.81)	−0.33 (0.76)	1
Subjugation	0.16	0.06–0.27	5.12 (0.01)	1.17 (3; 0.76)	0 (0)	0%	0.01–0.30	0.33 (0.75)	0.62 (0.59)	1
Unrelating standards	0.15	0.03–0.26	3.97 (0.02)	1.54 (3; 0.68)	0 (0)	0%	0.03–0.26	−0.33 (0.75)	−0.29 (0.79)	1
Enmeshment	0.13	−0.11 to 0.37	2.39 (0.14)	0.29 (2; 0.86)	0 (0)	0%	−0.11 to 0.37	−0.33 (1.00)	−0.54 (0.68)	2
Self‐sacrifice	0.10	0.00–0.20	3.06 (0.05)	1.26 (3; 0.73)	0 (0)	0%	0.00–0.20	0.33 (0.75)	0.84 (0.48)	2

* *Italics* EMS are those considered relevant according to the correlation coefficient.

#### 3.2.4 Sexual Abuse

The studies that provided the necessary data and were included in these meta‐analyses are Roemmele and Messman‐Moore (2011), Estévez et al. (2017), Mojallal et al. (2021), Celsi et al. (2021), Fernando et al. (2024), Lumley and Harkness (2007) and Harding et al. (2011). The schemas being tested are abuse, failure, social isolation, dependence, emotional deprivation, abandonment, self‐sacrifice, defectiveness, emotional inhibition, subjugation, vulnerability to harm, enmeshment and unrelenting standards (schemas for which there were data to conduct statistical analysis). Regrettably, none of the schemas met the established criteria or the required index fit; consequently, these results cannot be considered significant, as seen in the Supporting Information.

#### 3.2.5 IPV

The studies that provided the necessary data and were included in these meta‐analyses are McCarthy and Lumley (2012), Hassija et al. (2017), Obeid et al. (2019), Estévez et al. (2024) and Celsi et al. (2021) with the schema of subjugation, emotional deprivation, abuse, social isolation, dependence, defectiveness, self‐sacrifice, emotional inhibition, failure and abandonment (schemas for which there were data to conduct statistical analysis). However, only those with a correlation greater than 0.30 were considered relevant and are indicated in i*talics* in Table 5. The EMS most associated with IPV are subjugation, emotional deprivation, abuse and social isolation. These had a correlation coefficient between 0.34 and 0.30 (95% CI, 0.08–0.57; *p* < 0.01), with the majority of estimates being positive (100%). Therefore, the average outcome differed significantly from zero (*t* [*p*]). According to the *Q* test in abuse, social isolation and subjugation schemas, there was no significant amount of heterogeneity in the true outcomes; instead, in the emotional deprivation schema, the true outcomes appear to be heterogeneous (*Q* [df; *p*], tau^2^, *I* ^2^). The 95% prediction interval for the true outcomes ranges between 0.14 and 0.82. An examination of the studentized residuals revealed that none of the studies had a value larger than ±2.39–2.49; hence, there was no indication of outliers in the context of these models. The rank correlation and the regression test do not indicate any funnel plot asymmetry in all significant schemas (Begg and Mazumdar, *p*; Egger's regression, *p*). The Trim and Fill method reports the need to add one or two studies for the subjugation and the social isolation schemas to contrast the publication bias, but such studies were not found in the literature. (See the forest and funnel plot in the Supporting Information).

**TABLE 5:** The correlation coefficient of the schema in victims of IPV*.

EMS	Correlation coefficient	Lower limit–upper limit	*t* (*p*)	*Q* (df, *p*)	Tau^2^ (tau)	*I* ^2^	Prediction interval	Begg and Mazumdar (*p*)	Egger's regression (*p*)	Trim and fill
*Subjugation*	0.34	0.22–0.46	9.20 (0.003)	5.37 (3; 0.14)	0.002 (0.048)	42.84%	0.14–0.52	−0.33 (0.75)	−1.26 (0.33)	1
*Emotional deprivation*	0.33	0.08–0.57	4.26 (0.02)	16.84 (3; < 0.001)	0.01 (0.13)	85.98%	−0.17 to 0.82	0.00 (1.00)	−0.70 (0.55)	0
*Abuse*	0.30	0.17–0.43	10.1 (0.01)	0.128 (2; 0.93)	0 (0.01)	0%	0.17–0.43	0.33 (1.00)	0.005 (0.99)	0
*Social isolation*	0.30	0.17–0.43	9.81 (0.01)	1.98 (3; 0.37)	0 (0.001)	0.05%	0.16–0.42	−1.00 (0.33)	−1.41 (0.39)	2
Abandonment	0.25	0.15–0.36	6.79 (0.002)	8.87 (4; 0.06)	0.003 (0.06)	53.54%	0.05–0.44	−0.20 (0.81)	−0.56 (0.61)	3
Defectiveness	0.25	−0.24 to 0.73	2.18 (0.16)	24.17 (2; < 0.001)	0.03 (0.18)	91.64%	−0.68 to 1.17	−0.33 (1.00)	−1.58 (0.35)	0
Dependence	0.23	−0.19 to 0.66	2.35 (0.14)	21.41 (2; < 0.001)	0.02 (0.15)	89.07%	−0.57 to 1.03	−0.33 (1.00)	−1.09 (0.47)	0
Emotional inhibition	0.21	−0.26 to 0.67	1.91 (0.19)	19.77 (2; < 0.001)	0.03 (0.17)	90.67%	−0.67 to 1.08	−0.33 (1.00)	−1.92 (0.30)	2
Failure	0.14	−0.07 to 0.35	2.84 (0.10)	0.004 (2; 0.06)	4.58 (2; 0.10)	56.19%	−0.20 to 0.48	−0.33 (1.00)	0.12 (0.92)	0
Self‐sacrifice	0.06	−0.02 to 0.15	2.45 (0.09)	0.13 (3; 0.98)	0 (0)	0%	−0.02 to 0.15	0.00 (1.00)	0.02 (0.98)	1

* *Italics* EMS are those considered relevant according to the correlation coefficient.

## 4 Discussions

### 4.1 EMS in Survivors

This review analysed a total of 20 studies, focusing on specific research questions, including the identification of the most prevalent EMS among survivors of child abuse and IPV. The findings indicate that the most common EMS in this population are self‐sacrifice, unrelenting standards, mistrust and abuse, abandonment, dependence and vulnerability to harm. The self‐sacrifice and unrelenting standards schemas are particularly prevalent. The self‐sacrifice schema, which belongs to the other‐directedness domain, involves prioritizing other's needs over one's own. Survivors often develop the belief that their own needs are less important, which may stem from their abusive experiences. This mindset fosters a continued attachment to abusive caregivers or partners (Farazmand et al. 2015). Individuals with this schema are especially vulnerable to feelings of guilt and may blame themselves for their abuse, believing they could have prevented it by behaving differently (Glenn 1995; Naismith et al. 2022; Gonzalez 2017). This guilt can lead them to remain in abusive relationships, fearing that leaving would cause harm to the abuser (Pugliese et al. 2023a, 2023b). The unrelenting standards schema, part of the Over‐vigilance and Inhibition domain, is characterized by excessively high standards or perfectionism, often developed in response to critical or demanding caregivers. Survivors of abuse may have been subjected to unrealistic expectations and learned that their worth depended on meeting these standards. Failure to do so could result in violence or mistreatment (Askari 2018; Taskale and Soygut 2017). As a psychological defence, these individuals may develop intense self‐criticism, believing that perfection is the only way to avoid negative consequences (Naismith et al. 2022; Nagy et al. 2023; Sharhabani‐Arzy et al. 2005). They may also internalize the belief that they were ‘not good enough’ and therefore deserved the abuse they endured (Lynch 2013; Clark et al. 2010).

The mistrust/abuse and abandonment schemas are also closely related. A history of violence often leads survivors to develop a deeply negative view of relationships, believing betrayal and suffering are inevitable. This conviction may prevent them from seeking healthier relationships, as they assume all people will mistreat them (Zapcic et al. 2023; Klein et al. 2023). The abandonment schema, on the other hand, fosters the expectation that others will eventually leave, making survivors feel undesirable and unworthy of love (Louis and Reyes 2023; Upenieks and Ford‐Robertson 2022; Evgin and Sümen 2021; Xie et al. 2021). This can cause individuals to stay in dysfunctional relationships to avoid abandonment at any cost.

The dependence schema leads individuals to believe they cannot cope with life on their own. Survivors of abuse often develop a sense of helplessness and become increasingly reliant on others for support (Wright et al. 2021). If the abuser is a caregiver, the survivor may feel incapable of leaving the relationship, believing they would not survive without them (Crapolicchio et al. 2021).

The vulnerability to harm schema involves a pervasive fear of imminent danger. Abuse survivors may see the world as inherently threatening and believe they cannot protect themselves (Senkans et al. 2020; Atmaca and Gençöz 2016). This type of schema can lead to a maintenance cycle within relationships: women perceive themselves as fragile and vulnerable (so needing external support) and look for partners perceived as strong and capable of protecting. Sometimes, though, these partners can be abusive and violent, and this can keep the maintenance cycle alive.

In the context of this study, moderation analyses were conducted for sex, age and culture to understand the variations in the prevalence of specific EMS based on these variables. According to the results, the self‐sacrifice schema is affected by age. Specifically, it increases with age, particularly among individuals aged 35–40. A possible explanation for this phenomenon could be that women at this age are generally born in a social and familiar context characterized by different expectations and demands compared to the younger generations (Habibnejad et al. 2023), which encourages prioritizing family over personal or professional aspirations. Moreover, the self‐sacrifice schema could develop and intensify itself with the advancing age in response to the increasing environmental demands, which can be many and weighty (Güler and Yüksel 2021). Unrelenting standards are more prevalent in younger individuals but decline with age, possibly due to societal pressures for younger generations to excel (Habibnejad et al. 2023; ISTAT 2024). This schema is also more common in non‐European countries, particularly in the USA and Canada, where individual success and productivity are highly emphasized (Supyan 2022). Dependence increases with age and is more widespread in European cultures, where family interdependence is strong (Torres et al. 2008). In contrast, cultures emphasizing individualism, such as those in North America, show lower prevalence rates (Björklund et al. 2006). Vulnerability to harm also increases with age, suggesting that life experiences may reinforce perceptions of fragility (Betz et al. 2020; Schulte et al. 2021). Emotional deprivation is more prevalent in older women and European cultures, possibly due to reduced social support and societal expectations of self‐sufficiency (Körük 2017; von Wendorff et al. 2025; Tan et al. 2025; Freak‐Poli et al. 2025).

### 4.2 EMS in Psychological Violence

Survivors of psychological abuse commonly exhibit emotional deprivation, abuse, social isolation, failure, abandonment, emotional inhibition, vulnerability to harm, defectiveness, dependence and subjugation schemas. Emotional deprivation leads survivors to believe they do not deserve love or care, reinforcing a cycle in which they seek out emotionally unavailable partners, confirming their core self‐belief. The maintenance of this cycle does not promote healthy relationships (Eken 2018). Abuse schema fosters a belief that others will exploit them, inducing people to assume a passive and remissive position within relationships, making survivors more likely to remain in abusive relationships perpetuating the idea that the cycle will not be interrupted and abusive and violent partners will benefit from them (Eken 2018). Social isolation develops when survivors have the belief ‘I am different from others’. This belief leads them to tolerate abuse to avoid loneliness because being in an abusive relationships is better than not having it at all. It could also happen that, due to the fear of being socially isolated, the survivor excessively adapts to every situation (including abusive relationships), sacrificing their individuality. Furthermore, survivors may feel that others do not understand them due to their differences, leading them to believe that seeking help is futile (Barazandeh et al. 2016). The failure schema is represented by the belief of ‘being inferior or inadequate compared to others’. This belief leads to low self‐worth and passivity. Revictimization occurs as a result of disinvestment in relationships, where the individual makes no changes and continues to test whether others will make him feel inadequate. In abusive relationships, survivors often reinforce their beliefs of failure by staying in painful situations they feel unable to escape from (Calvete et al. 2007). The abandonment schema is supported by the belief that ‘others are unreliable, unstable, and unpredictable and can abandon you at any moment’. This schema results in behaviours that either avoid intimacy or create controlling relationships, leading the partner to distance and confirming the abandoning scenario (Barazandeh et al. 2016). Another schema that can stem from psychological abuse is the emotional inhibition schema. Survivors may suppress their spontaneous behaviours and emotions to avoid losing control or facing criticism from others, as controlling parenting has inhibited their ability to express emotions such as anger or explore new environments. In adulthood, this inhibition prevents individuals from escaping the situation due to their inability to perceive the injustice of the abuse. Unaware of the injustice of the abuse, trauma relaboration happens through rationalization by justifying the underlying motivation (Molendijk 2023; Dassylva et al. 2025). Vulnerability to harm leads survivors to feel a constant sense of danger, stemming from the belief that ‘I am fragile and unable to cope with difficulties, danger, or unexpected events’. This mindset often develops due to overly protective parents who stifle autonomy and exploration (Young et al. 2003). Results shed light on the enmeshment schema and the poor sense of identity consequent to having experienced psychological abuse in childhood; people who have a scarce sense of identity often experience a sense of emptiness and disorientation, believing that it is not possible to live without having someone else close to, until creating symbiotic bonds where survivors lose the sense of self. Parents of survivors high in this schema are often entangled in symbiotic relationships with their children, leading them not to create a robust sense of self and the perception of being able to manage on one's own (Gélinas et al. 2025). Experiencing psychological abuse can structure the dependence schema, which leads survivors to not feel able in several areas of their lives; this perception of incapacity can lead the person to delegate specific responsibilities to significant others and to avoid situations that imply the use of personal abilities. The dependence schema moves from being dependent on parents to partners, repeating the schema and the behaviours that characterize it (Wright et al. 2021). Finally, according to the results, the subjugation schema is relevant to these survivors. Survivors high in this schema often repress their needs and their emotions, displaying compliant behaviours to avoid expressing anger and risking negative reactions such as being abandoned. This conduct survivors to adapt to abusive behaviour to reduce conflicts or escalating violence, leading them to believe that their needs and emotions are unworthy of attention or that expressing these emotions may lead to painful consequences (Pietri and Bonnet 2017).

### 4.3 EMS in Physical Violence

Survivors of physical abuse often exhibit emotional deprivation, social isolation and vulnerability to harm. They may struggle to rely on others for emotional support, feel disconnected from others and society and perceive the world as an unsafe and threatening place. Having a history of parenting characterized by physical abuse can lead the individual to experience the unpredictability of the relationship, which no longer becomes a safe space to return to in times of difficulty but rather an unstable and unpredictable place where there is no support and care (Zufferey 2022; Roman and Ryan 2021).

### 4.4 EMS in Sexual Violence

Concerning sexual abuse, no analysed schemas show a correlation that satisfies the stabilized criteria, so the correlation is not significant. These results can be explained by the limited amount of studies present in the literature and the unique nature of the abuse, which can be more difficult to detect compared to other forms of violence. In fact, detecting sexual abuse can be more challenging due to the reluctance to recognize and talk about this sensitive topic.

### 4.5 EMS in IPV

Schemas mainly significant in IPV are subjugation, emotional deprivation, abuse and social isolation. It is noteworthy that people high in these schemas often are not aware of what they are going through, specifically of being survivors or considering violence as part of being in a relationship. In detail, subjugation comes from that context where, since childhood, there has not been the chance to freely express themselves, developing a tendency to adapt passively to others' needs despite their well‐being. In a violent and abusive relationship, this passivity becomes fertile ground for control by the abusive partner (Dutton 2011). Similarly, emotional deprivation emerges in those contexts where it lacks emotional support, reinforcing the idea of being inadequate and unable to ask for help. People high in the emotional deprivation schema do not recognize that they deserve healthy relationships because the partner emotionally manipulates their self‐perception (Carney and Barner 2012). This schema pushes survivors to tolerate violent behaviours, accepting them as part of the relationships and without assuming a defense position (Storm‐Mathisen 2024). The abuse schema, which develops when survivors have been exposed to traumatic experiences of abuse, structures the whole perception of the identity. People high in this schema think they deserve violence, perceiving abuse as unavoidable and often as a sign of love. Therefore, survivors do not only accept abuse as expected, but they become incapable of reacting or seeking help, trapped in a violent cycle perceived as impossible to interrupt (Walker 2009). Furthermore, social isolation is often a form of control used by the abusive partner who tries to create a separation between the survivor and friends, family and external support. Survivors exhibiting high levels of this schema experienced, over time and since childhood, social isolation, developing a wish for approval at any cost. When this schema is present in violent relationships, the isolation reduces the chance of seeking help and support, fostering a perception of loneliness and vulnerability in which the survivor may feel trapped (Taccini et al. 2024). Our findings are consistent with the cognitive model of affective dependency proposed by Pugliese et al. (2023a, 2023b), which identifies this condition as a risk factor for IPV. The model outlines specific goals and anti‐goals that contribute to victims' difficulties in leaving abusive partners, leading to different dependency profiles: *s*aver, unworthy, traumatic and mixed. These goals may include feeling valued, saving the partner or ensuring emotional security; the corresponding anti‐goals involve fears of losing dignity, causing the partner suffering or being left alone and unprotected. Victims typically go through three conflictual phases: an absent phase, in which there is no awareness of relational distress and the costs are visible only to outsiders; an alternating phase, marked by internal vacillation between leaving and staying; and an akrasic phase, where the individual recognizes the toxic nature of the relationship but feels unable to end it. According to Askeland et al. (2011), such patterns may stem from an unconscious drive to resolve childhood trauma, which leads individuals to be more attracted to problematic rather than functional partners. Moreover, recent research by Silvestri et al. (2025) has shown that early exposure to violence can lead to an overestimation of the partner's trustworthiness, particularly when past attachment figures were dominant. Perizzolo Pointet et al. (2021) noted that this dynamic may reflect a protective adaptation aimed at survival, expressed through submissive behaviour.

### 4.6 The Link Between Childhood Abuse and Later Victimization

Although in our study it was not possible to statistically establish a link between childhood abuse and subsequent experiences of revictimization, we believe that some of the findings may offer hypotheses for further investigation. For example, certain schemas from the first domain, such as abuse, abandonment and emotional deprivation, which are commonly observed in cases of childhood abuse, could, over time, manifest as compliant surrender coping mode, expressed through self‐sacrifice, submission and dependence schemas, which are prevalent in survivors of IPV. This seems to be further supported by the results of the meta‐regressions, which examine the influence of age on these secondary schemas. Moreover, this hypothesis appears to be consistent with the distinction made by Young et al. (2003) between unconditional and conditional schemas. The former are those developed during childhood in response to the relationship with primary attachment figures, while the latter, which include the submission and self‐sacrifice schemas, develop later based on relationships outside the family of origin and serve a coping function in relation to the other schemas. Some studies have confirmed that all unconditional EMS, except for emotional inhibition, are more strongly associated with childhood maltreatment than conditional EMS (Cecero et al. 2004; McCarthy and Lumley 2012). Finally, the study by Messman‐Moore and Coates (2007) demonstrated a link between childhood psychological abuse, mistrust/abuse, abandonment and defectiveness schemas, and subsequent interpersonal conflicts, while the study by Crawford and Wright (2007) shows a connection between childhood psychological abuse, mistrust/abuse, self‐sacrifice and emotional inhibition schemas and IPV. In this way, early adverse experiences intertwine with coping strategies and maladaptive schemas, reinforcing the cyclical nature of violence.

### 4.7 Limitations

This study has some limitations. First, the objectives of the studies we included differed from ours, which meant we could not obtain consistent data in all cases. For instance, some studies incorporated EMS within mediation models involving other variables (e.g., parenting styles, emotions of guilt and shame, risky sexual behaviours, emotional distress), while others aimed to assess the impact of abuse and maladaptive schemas on the trajectories of psychopathological development. As a result, the necessary data for our meta‐analysis were not always available, or when they were, they often represented marginal data for our purposes. This discrepancy in research objectives and data availability meant that we could not perform all the analyses we had initially planned, and in some cases, we had to work with a smaller number of studies than anticipated. We contacted all the first or corresponding authors, but not everyone replied, which is especially evident in cases of sexual abuse. Additionally, not all studies included a control group, which limited the analyses. Furthermore, most studies included only women, which limits the generalizability of the results. Although an implicit goal was to establish a connection between childhood abuse and subsequent violence in adulthood, there was insufficient data to conduct the necessary statistical analyses. As a result, we could only develop qualitative clinical hypotheses regarding this issue. Finally, we lack information on the samples' personal characteristics and life history, including demographic details, other adverse experiences and coping styles. Only through future studies that establish these objectives will we achieve complete answers.

## 5 Conclusions

Despite the limitations, this study offers valuable insights and food for thought to improve clinical practice. We now have a systematic collection of common EMS found in survivors, along with the types of abuse associated with these specific EMS. Its understanding will provide clearer insights into the motivations behind the behaviours of individuals who remain in abusive relationships. The main findings indicate that abuse creates specific EMS that can persist throughout a person's life. These EMS influence how the individual perceives the world, leading them to constantly find themselves in similar situations. As a result, they continue to reinforce and maintain their maladaptive state. Understanding the formation of EMS is essential because it shapes specific developmental paths. For example, an individual whose primary EMS is self‐sacrifice tends to prioritize the needs and desires of others over their own. This inclination stems from a desire to maintain closeness and avoid feelings of guilt, even if it means staying in an abusive relationship with a partner who fails to recognize the needs of others. Consequently, this partner may also be more likely to choose partners who are willing to sacrifice their well‐being, possibly due to an entitlement schema. This schema, along with those of submission and dependence, may also represent a link between childhood abuse and adult abuse. As these schemas tend to increase with age, they not only serve as risk factors but may also reflect a compliant surrender coping mode in response to the maladaptive schemas from the first domain. This information makes it easier to identify patients' dysfunctional beliefs about themselves, the world and others, which are essential in cognitive‐behavioural therapy. Once life experiences, associated maladaptive schemas and dysfunctional beliefs have been identified, more effective individualized psychotherapeutic management can be achieved. Following the ST approach, the Imagery Rescripting technique could be utilized to treat these patients.

## Author Contributions

Conceptualization: A.U., A.A. and S.I.; methodology and formal analysis: A.U.; writing – original draft preparation: A.U., M.F., G.A., A.C.F., L.F. and T.M.; writing – review and editing: A.U., M.F., A.A., S.I. and A.G.; supervision: A.G. All authors contributed to manuscript writing and approved the manuscript for submission.

## Ethics Statement

The authors have nothing to report.

## Conflicts of Interest

The authors declare no conflicts of interest.

## Supporting information


**Data S1.** Qualitative Results
**Data S2**. Funnel Plot of Self‐Sacrifice
**Data S3**. Funnel Plot of Unrelenting Standards
**Data S4**. Funnel PlotAbuse
**Data S5**. Funnel Plot Abandonment
**Data S6**. Funnel Plot Dependence
**Data S7**. Funnel Plot Vulnerability to Harm
**Data S8**. Results of the moderation analysis in victims’ schemas
**Data S9**. Scatter Plot of the moderation effect of age on Self‐Sacrifice
**Data S10**. Scatter Plot of the moderation effect of age on Unrelenting Standards
**Data S11**. Scatter Plot of the moderation effect of country on Unrelenting Standards
**Data S12**. Scatter Plot of the moderation effect of age on Dependence
**Data S13**. Scatter Plot of the moderation effect of country on Dependence
**Data S14**. Scatter Plot of the moderation effect of age on Vulnerability to Harm
**Data S15**. Scatter Plot of the moderation effect of age on Emotional Deprivation
**Data S16**. Scatter Plot of the moderation effect of gender on Emotional Deprivation
**Data S17**. Scatter Plot of the moderation effect of country on Emotional Deprivation
**Data S18**. Forest Plot of Emotional Deprivation in psychological abuse victims
**Data S19**. Funnel Plot of Emotional Deprivation in psychological abuse victims
**Data S20**. Forest Plot of Abuse in psychological abuse victims
**Data S21**. Funnel Plot of Abuse in psychological abuse victims
**Data S22**. Forest Plot of Social Isolation in psychological abuse victims
**Data S23**. Funnel Plot of Social Isolation in psychological abuse victims
**Data S24**. Forest Plot of Failure in psychological abuse victims.
**Data S25**. Funnel Plot of Failure in psychological abuse victims
**Data S26**. Forest Plot of Abandonment in psychological abuse victims
**Data S27**. Funnel Plot of Abandonment in psychological abuse victims
**Data S28**. Forest Plot of Emotional Inhibition in psychological abuse victims
**Data S29**. Funnel Plot of Emotional Inhibition in psychological abuse victims
**Data S30**. Forest Plot of Vulnerability to Harm in psychological abuse victims
**Data S31**. Funnel Plot of Vulnerability to Harm in psychological abuse victims
**Data S32**. Forest Plot of Defectiveness in psychological abuse victims
**Data S33**. Funnel Plot of Defectiveness in psychological abuse victims
**Data S34**. Forest Plot of Dependence in psychological abuse victims
**Data S35**. Funnel Plot of Dependence in psychological abuse victims
**Data S36**. Forest Plot of Subjugation in psychological abuse victims
**Data S37**. Funnel Plot of Subjugation in psychological abuse victims
**Data S38**. Forest Plot of Emotional Deprivation in physical abuse victims
**Data S39**. Funnel Plot of Emotional Deprivation in physical abuse victims
**Data S40**. Forest Plot of Social Isolation in physical abuse victims
**Data S41**. Funnel Plot of Social Isolation in physical abuse victims
**Data S42**. Forest Plot of Vulnerability to Harm in physical abuse victims
**Data S43**. Funnel Plot of Vulnerability to Harm in physical abuse victims
**Data S44**. The correlation coefficient of the schema for victims of sexual abuse
**Data S45**. Forest Plot of subjugation in IPV victims
**Data S46**. Funnel Plot of subjugation in IPV victims
**Data S47**. Forest plot of emotional deprivation in IPV victims
**Data S48**. Funnel plot of emotional deprivation in IPV victims
**Data S49**. Forest plot of abuse in IPV victims
**Data S50**. Funnel plot of abuse in IPV victims
**Data S51**. Forest plot of social isolation in IPV victims
**Data S52**. Funnel plot of social isolation in IPV victims

## Data Availability

The study data are available in the article and its Supporting Information.

## References

[cpp70114-bib-0001] Anda, R. F. , C. L. Whitfield , V. J. Felitti , et al. 2002. “Adverse Childhood Experiences, Alcoholic Parents, and Later Risk of Alcoholism and Depression.” Psychiatric Services 53, no. 8: 1001–1009. 10.1176/appi.ps.53.8.1001. 12161676

[cpp70114-bib-0002] Askari, I. 2018. “Early Maladaptive Schemas and Cognitive‐Behavioral Aspect of Anger: Schema Model Perspective.” Journal of Rational‐Emotive & Cognitive‐Behavior Therapy 37, no. 3: 262–283. 10.1007/s10942-018-0311-9.

[cpp70114-bib-0003] Askeland, I. R. , A. Evang , and T. Heir . 2011. “Association of Violence Against Partner and Former Victim Experiences: A Sample of Clients Voluntarily Attending Therapy.” Journal of Interpersonal Violence 26: 1095–1110. 10.1177/0886260510368152. 20587478

[cpp70114-bib-0004] Atmaca, S. , and T. Gençöz . 2016. “Exploring Revictimization Process Among Turkish Women: The Role of Early Maladaptive Schemas on the Link Between Child Abuse and Partner Violence.” Child Abuse & Neglect 52: 85–93. 10.1016/j.chiabu.2016.01.004. 26826949

[cpp70114-bib-0005] Azadfar, Z. , G. Rossi , E. Dierckx , et al. 2025. “Early Maladaptive Schemas as Mediators Between Childhood Maltreatment and Adult Psychopathology in Psychiatric Inpatients.” Child Abuse & Neglect 160: 107238. 10.1016/j.chiabu.2024.107238. 39754989

[cpp70114-bib-0006] Bakhtiari Moghaddam, S. , and F. Jomehri . 2016. “Cross‐Cultural Comparison of Early Maladaptive Schemas, Resilience and Quality of Life in Students.” Review of European Studies 8, no. 2: 236. 10.5539/res.v8n2p236.

[cpp70114-bib-0007] Bamber, M. , and R. McMahon . 2008. “Danger—Early Maladaptive Schemas at Work!: The Role of Early Maladaptive Schemas in Career Choice and the Development of Occupational Stress in Health Workers.” Clinical Psychology & Psychotherapy 15, no. 2: 96–112. 10.1002/cpp.564. 19115432

[cpp70114-bib-0008] Barazandeh, H. , D. W. Kissane , N. Saeedi , and M. Gordon . 2016. “A Systematic Review of the Relationship Between Early Maladaptive Schemas and Borderline Personality Disorder/Traits.” Personality and Individual Differences 94: 130–139. 10.1016/j.paid.2016.01.021.

[cpp70114-bib-0009] Beck, A. T. 1979. Cognitive Therapy of Depression. Guilford Press.

[cpp70114-bib-0010] Berber Çelik, Ç. , and H. Odacı . 2019. “Does Child Abuse Have an Impact on Self‐Esteem, Depression, Anxiety and Stress Conditions of Individuals?” International Journal of Social Psychiatry 66, no. 2: 171–178. 10.1177/0020764019894618. 31856622

[cpp70114-bib-0011] Bernstein, D. P. , J. A. Stein , M. D. Newcomb , et al. 2003. “Development and Validation of a Brief Screening Version of the Childhood Trauma Questionnaire.” Child Abuse & Neglect 27, no. 2: 169–190. 10.1016/s0145-2134(02)00541-0. 12615092

[cpp70114-bib-0012] Betz, L. T. , N. Penzel , M. Rosen , and J. Kambeitz . 2020. “Relationships Between Childhood Trauma and Perceived Stress in the General Population: A Network Perspective.” Psychological Medicine 51, no. 15: 2696–2706. 10.1017/s003329172000135x. 32404227

[cpp70114-bib-0013] Beynon, R. , M. M. Leeflang , S. McDonald , et al. 2013. “Search Strategies to Identify Diagnostic Accuracy Studies in MEDLINE and EMBASE.” Cochrane Database of Systematic Reviews 2013, no. 9: MR000022. 10.1002/14651858.mr000022.pub3. 24022476 PMC7390022

[cpp70114-bib-0014] Björklund, A. , D. K. Ginther , and M. Sundström . 2006. “Family Structure and Child Outcomes in the USA and Sweden.” Journal of Population Economics 20, no. 1: 183–201. 10.1007/s00148-006-0094-7.

[cpp70114-bib-0015] Boog, M. , M. Visser , L. Clarijs , I. Franken , and A. Arntz . 2024. “One‐Year Follow‐Up: Schema Therapy for Patients With Borderline Personality Disorder and Comorbid Alcohol Use Disorder.” Clinical Psychology & Psychotherapy 31, no. 4: e3040. 10.1002/cpp.3040. 39140112

[cpp70114-bib-0016] Borenstein, M. , L. V. Hedges , J. P. Higgins , and H. R. Rothstein . 2010. “A Basic Introduction to Fixed‐Effect and Random‐Effects Models for Meta‐Analysis.” Research Synthesis Methods 1, no. 2: 97–111. 10.1002/jrsm.12. 26061376

[cpp70114-bib-0017] Borenstein, M. , L. V. Hedges , J. P. Higgins , and H. R. Rothstein . 2011. Introduction to Meta‐Analysis. John Wiley & Sons.

[cpp70114-bib-0018] Borges, J. L. , and D. D. Dell'Aglio . 2020. “Early Maladaptive Schemas as Mediators Between Child Maltreatment and Dating Violence in Adolescence. [Esquemas Iniciais Desadaptativos Como Mediadores Entre os Maus Tratos na Infância e a Violência no Namoro na Adolescência].” Ciência & Saúde Coletiva 25, no. 8: 3119–3130. 10.1590/1413-81232020258.24992018. 32785547

[cpp70114-bib-0019] Bosmans, G. , C. Braet , and L. Van Vlierberghe . 2010. “Attachment and Symptoms of Psychopathology: Early Maladaptive Schemas as a Cognitive Link?” Clinical Psychology & Psychotherapy 17, no. 5: 374–385. 10.1002/cpp.667. 20013761

[cpp70114-bib-0020] Boyda, D. , D. McFeeters , K. Dhingra , and L. Rhoden . 2018. “Childhood Maltreatment and Psychotic Experiences: Exploring the Specificity of Early Maladaptive Schemas.” Journal of Clinical Psychology 74, no. 12: 2287–2301. 10.1002/jclp.22690. 30101974

[cpp70114-bib-0021] Calvete, E. , S. Corral , and A. Estévez . 2007. “Cognitive and Coping Mechanisms in the Interplay Between Intimate Partner Violence and Depression.” Anxiety, Stress, and Coping 20, no. 4: 369–382. 10.1080/10615800701628850. 17999237

[cpp70114-bib-0022] Calvete, E. , L. Fernández‐González , I. Orue , and T. D. Little . 2018. “Exposure to Family Violence and Dating Violence Perpetration in Adolescents: Potential Cognitive and Emotional Mechanisms.” Psychology of Violence 8, no. 1: 67–75. 10.1037/vio0000076.

[cpp70114-bib-0023] Cardoso, B. L. , A. F. Lima , F. R. Costa , C. Loose , X. Liu , and M. A. Fabris . 2024. “Sociocultural Implications in the Development of Early Maladaptive Schemas in Adolescents Belonging to Sexual and Gender Minorities.” International Journal of Environmental Research and Public Health 21, no. 8: 971. 10.3390/ijerph21080971. 39200582 PMC11353358

[cpp70114-bib-0024] Carney, M. M. , and J. R. Barner . 2012. “Prevalence of Partner Abuse: Rates of Emotional Abuse and Control.” Partner Abuse 3, no. 3: 286–335. 10.1891/1946-6560.3.3.286.

[cpp70114-bib-0025] Celsi, L. , F. G. Paleari , and F. D. Fincham . 2021. “Adverse Childhood Experiences and Early Maladaptive Schemas as Predictors of Cyber Dating Abuse: An Actor‐Partner Interdependence Mediation Model Approach.” Frontiers in Psychology 12: 623646. 10.3389/fpsyg.2021.623646. 33815208 PMC8012817

[cpp70114-bib-0026] Chapman, D. P. , C. L. Whitfield , V. J. Felitti , S. R. Dube , V. J. Edwards , and R. F. Anda . 2004. “Adverse Childhood Experiences and the Risk of Depressive Disorders in Adulthood.” Journal of Affective Disorders 82, no. 2: 217–225. 10.1016/j.jad.2003.12.013. 15488250

[cpp70114-bib-0027] Clark, D. M. 1996. “Panic Disorder: From Theory to Therapy.” In Frontiers of Cognitive Therapy, edited by P. M. Salkovskis , 318–344. Guilford Press.

[cpp70114-bib-0028] Clark, J. J. , G. Sprang , B. Freer , and A. Whitt‐Woosley . 2010. “‘Better Than Nothing’ Is Not Good Enough: Challenges to Introducing Evidence‐Based Approaches for Traumatized Populations.” Journal of Evaluation in Clinical Practice 18, no. 2: 352–359. 10.1111/j.1365-2753.2010.01567.x. 21143348

[cpp70114-bib-0029] Cockram, D. M. , P. D. Drummond , and C. W. Lee . 2010. “Role and Treatment of Early Maladaptive Schemas in Vietnam Veterans With PTSD.” Clinical Psychology & Psychotherapy 17, no. 3: 165–182. 10.1002/cpp.690. 20486158

[cpp70114-bib-0030] Crapolicchio, E. , C. Regalia , G. A. Bernardo , and V. Cinquegrana . 2021. “The Role of Relational Dependence, Forgiveness and Hope on the Intention to Return With an Abusive Partner.” Journal of Social and Personal Relationships 38, no. 9: 2474–2493. 10.1177/02654075211011546.

[cpp70114-bib-0031] Crawford, E. , and M. O. Wright . 2007. “The Impact of Childhood Psychological Maltreatment on Interpersonal Schemas and Subsequent Experiences of Relationship Aggression.” Journal of Emotional Abuse 7, no. 2: 93–116. 10.1300/j135v07n02_06.

[cpp70114-bib-0032] Csukly, G. , R. Telek , D. Filipovits , B. Takács , Z. Unoka , and L. Simon . 2011. “What Is the Relationship Between the Recognition of Emotions and Core Beliefs: Associations Between the Recognition of Emotions in Facial Expressions and the Maladaptive Schemas in Depressed Patients.” Journal of Behavior Therapy and Experimental Psychiatry 42, no. 1: 129–137. 10.1016/j.jbtep.2010.08.003. 20828674

[cpp70114-bib-0033] Dassylva, O. , L. M. Amedee , A. Paradis , and M. Hebert . 2025. “Coping Patterns Among Sexually Abused Children: A Latent Profile Analysis.” Children and Youth Services Review 169: 108083. 10.1016/j.childyouth.2024.108083.

[cpp70114-bib-0034] Delattre, V. , D. Servant , S. Rusinek , et al. 2004. “Les Schémas Précoces Dysfonctionnels: Étude Chez des Patients Adultes Souffrant D'un Trouble Anxieux [the Early Maladaptive Schemas: A Study in Adult Patients With Anxiety Disorders].” L'encephale 30, no. 3: 255–258. 10.1016/s0013-7006(04)95437-1. 15235523

[cpp70114-bib-0035] Dutton, D. G. 2011. Rethinking Domestic Violence. Ubc Press.

[cpp70114-bib-0036] Ehlers, A. , and D. M. Clark . 2000. “A Cognitive Model of Posttraumatic Stress Disorder.” Behaviour Research and Therapy 38, no. 4: 319–345. 10.1016/s0005-7967(99)00123-0. 10761279

[cpp70114-bib-0037] Eken, E. 2018. “The Role of Early Maladaptive Schemas on Romantic Relationships: A Review Study.” PEOPLE : International Journal of Social Sciences 3, no. 3: 108–123. 10.20319/pijss.2017.33.108123.

[cpp70114-bib-0038] Estévez, A. , P. Jauregui , J. Momeñe , and L. Macía . 2024. “Association Between Gambling Motives, Violence and Early Maladaptive Schemas in Women With Gambling Disorder.” Journal of Gambling Studies 40, no. 3: 1701–1718. 10.1007/s10899-024-10285-8. 38427267 PMC11390860

[cpp70114-bib-0039] Estévez, A. , P. Jauregui , N. Ozerinjauregi , and D. Herrero‐Fernández . 2017. “The Role of Early Maladaptive Schemas in the Appearance of Psychological Symptomatology in Adult Women Victims of Child Abuse.” Journal of Child Sexual Abuse 26, no. 8: 889–909. 10.1080/10538712.2017.1365318. 28972452

[cpp70114-bib-0040] Estévez, A. , N. Ozerinjauregi , P. Jauregui , and U. Orbegozo . 2015. “Mediating Role of Parenting Styles Between Emotional Abuse and Neglect, and the Occurrence of EMSs Among Sexual Abuse Victims.” Journal of Child Custody 13, no. 1: 52–71. 10.1080/15379418.2016.1133256.

[cpp70114-bib-0041] Evgin, D. , and A. Sümen . 2021. “Childhood Abuse, Neglect, Codependency, and Affecting Factors in Nursing and Child Development Students.” Perspectives in Psychiatric Care 58, no. 4: 1357–1371. 10.1111/ppc.12938. 34448498

[cpp70114-bib-0042] Fabio, R. A. , L. Natolo , T. Caprì , C. Mento , and G. Picciotto . 2024. “Exploring the Impact of Adverse Childhood Experiences on Health and Cognitive Functions in Older Adults.” Journal of Health Psychology. 10.1177/13591053241277369. 39295237

[cpp70114-bib-0043] Farazmand, S. , P. Mohammadkhani , A. Pourshahbaz , and B. Dolatshahi . 2015. “Mediating Role of Maladaptive Schemas Between Childhood Emotional Maltreatment and Psychological Distress Among College Students.” Practice in Clinical Psychology 3, no. 3: 203–211. http://jpcp.uswr.ac.ir/article‐1‐265‐en.html.

[cpp70114-bib-0044] Farrell, J. M. , I. A. Shaw , and M. A. Webber . 2009. “A Schema‐Focused Approach to Group Psychotherapy for Outpatients With Borderline Personality Disorder: A Randomized Controlled Trial.” Journal of Behavior Therapy and Experimental Psychiatry 40, no. 2: 317–328. 10.1016/j.jbtep.2009.01.002. 19176222

[cpp70114-bib-0045] Felitti, V. J. , R. F. Anda , D. Nordenberg , et al. 1998. “Relationship of Childhood Abuse and Household Dysfunction to Many of the Leading Causes of Death in Adults.” American Journal of Preventive Medicine 14, no. 4: 245–258. 10.1016/s0749-3797(98)00017-8. 9635069

[cpp70114-bib-0046] Fernando, S. K. , E. Quinlan , and J. Paparo . 2024. “Childhood Emotional Maltreatment and Romantic Relationship Satisfaction: The Mediating Role of Early Maladaptive Schemas.” Clinical Psychologist 28, no. 3: 317–330. 10.1080/13284207.2024.2415953.

[cpp70114-bib-0047] Finkelhor, D. , and A. Browne . 1985. “The Traumatic Impact of Child Sexual Abuse: A Conceptualization.” American Journal of Orthopsychiatry 55, no. 4: 530–541. 10.1111/j.1939-0025.1985.tb02703.x. 4073225

[cpp70114-bib-0048] Freak‐Poli, R. , H. L. Htun , A. B. Teshale , and C. Kung . 2025. “Understanding Loneliness After Widowhood: The Role of Social Isolation, Social Support, Self‐Efficacy, and Health‐Related Factors.” Archives of Gerontology and Geriatrics 129: 105692. 10.1016/j.archger.2024.105692. 39608049

[cpp70114-bib-0049] Garrido, E. F. , and H. N. Taussig . 2013. “Do Parenting Practices and Prosocial Peers Moderate the Association Between Intimate Partner Violence Exposure and Teen Dating Violence?” Psychology of Violence 3, no. 4: 354–366. 10.1037/a0034036. 25635230 PMC4307850

[cpp70114-bib-0050] Gélinas, J. , A. Claing , C. Dugal , et al. 2025. “Intergenerational Transmission of Childhood Interpersonal Trauma in Adults Entering Therapy for Intimate Partner Violence: The Role of Identity Diffusion.” Child Abuse & Neglect 161: 107258. 10.1016/j.chiabu.2025.107258. 39862647

[cpp70114-bib-0051] Glenn, L. S. 1995. “Guilt and Self‐Sacrifice: The Plight of the Better‐Off Sibling.” Journal of Contemporary Psychotherapy 25, no. 1: 61–75. 10.1007/bf02308669.

[cpp70114-bib-0052] Gonzalez, C. 2017. “Recovering Process From Child Sexual Abuse During Adulthood From an Integrative Approach to Solution‐Focused Therapy: A Case Study.” Journal of Child Sexual Abuse 26, no. 7: 785–805. 10.1080/10538712.2017.1354954. 28873043

[cpp70114-bib-0053] Güler, K. , and S. Yüksel . 2021. “The Relationship Between Gender Roles in Women and Early Maladaptive Schemas.” American Journal of Humanities and Social Sciences Research (AJHSSR) 5, no. 11: 7–19.

[cpp70114-bib-0054] Habibnejad, F. , F. Elyasi , R. Nikbakht , and Z. Shahhosseini . 2023. “The Factors Related to Gender Role Attitudes in Men and Women: A Narrative Review.” Current Psychosomatic Research 2, no. 1: 3–18. 10.32598/cpr.2.1.146.1.

[cpp70114-bib-0055] Harding, H. G. , E. E. Burns , and J. L. Jackson . 2011. “Identification of Child Sexual Abuse Survivor Subgroups Based on Early Maladaptive Schemas: Implications for Understanding Differences in Posttraumatic Stress Disorder Symptom Severity.” Cognitive Therapy and Research 36, no. 5: 560–575. 10.1007/s10608-011-9385-8.

[cpp70114-bib-0056] Hashemipoor, F. , F. Jafari , and R. Zabihi . 2019. “Maladaptive Schemas and Psychological Well‐Being in Premenopausal and Postmenopausal Women.” Menopausal Review 18, no. 1: 33–38. 10.5114/pm.2019.84155. PMC6528035 31114456

[cpp70114-bib-0057] Hassija, C. M. , D. Robinson , Y. Silva , and M. R. Lewin . 2017. “Dysfunctional Parenting and Intimate Partner Violence Perpetration and Victimization Among College Women: The Mediating Role of Schemas.” Journal of Family Violence 33, no. 1: 65–73. 10.1007/s10896-017-9942-3.

[cpp70114-bib-0058] Hedges, L. V. 1981. “Distribution Theory for Glass's Estimator of Effect Size and Related Estimators.” Journal of Educational Statistics 6, no. 2: 107. 10.2307/1164588.

[cpp70114-bib-0059] Higgins, J. P. , and S. G. Thompson . 2002. “Quantifying Heterogeneity in a Meta‐Analysis.” Statistics in Medicine 21, no. 11: 1539–1558. 10.1002/sim.1186. 12111919

[cpp70114-bib-0060] Higgins, J. P. T. and S. Green . 2011. “Cochrane Handbook for Systematic Reviews of Interventions Version 5.1.0.” The Cochrane Collaboration.

[cpp70114-bib-0061] Hoffart Lunding, S. , and A. Hoffart . 2014. “Perceived Parental Bonding, Early Maladaptive Schemas and Outcome in Schema Therapy of Cluster C Personality Problems.” Clinical Psychology & Psychotherapy 23, no. 2: 107–117. 10.1002/cpp.1938. 25425509

[cpp70114-bib-0062] Horsley, T. , O. Dingwall , and M. Sampson . 2011. “Checking Reference Lists to Find Additional Studies for Systematic Reviews.” Cochrane Database of Systematic Reviews 2011: 8. 10.1002/14651858.mr000026.pub2. PMC7388740 21833989

[cpp70114-bib-0063] Hosseinifard, S. M. , and N. Kaviani . 2015. “Comparing the Early Maladaptive Schemas, Attachment and Coping Styles in Opium and Stimulant Drugs Dependent Men in Kerman, Iran.” Addiction and Health 7, no. 1–2: 30–36. 26322208 PMC4530191

[cpp70114-bib-0064] Irkörücü, A. 2016. “Gender Difference in Early Maladaptive Schemas.” Ufuk Üniversitesi Sosyal Bilimler Enstitüsü Dergisi 5, no. 9: 103–119.

[cpp70114-bib-0065] Istituto Nazionale di Statistica (ISTAT) . 2024. “Rapporto Sulla Competitività dei Settori Produttivi ‐ Edizione 2024.” https://www.istat.it/statistiche‐per‐temi/focus/congiuntura/competitivita/.

[cpp70114-bib-0066] Iverson, K. M. , S. D. Litwack , S. L. Pineles , M. K. Suvak , R. A. Vaughn , and P. A. Resick . 2013. “Predictors of Intimate Partner Violence Revictimization: The Relative Impact of Distinct PTSD Symptoms, Dissociation, and Coping Strategies.” Journal of Traumatic Stress 26, no. 1: 102–110. 10.1002/jts.21781. 23417878

[cpp70114-bib-0067] Janoff‐Bulman, R. 1979. “The Aftermath of Victimization: Rebuilding Shattered Assumptions.” In Victims of Crime, 13–37. Sage Publications.

[cpp70114-bib-0068] Kar, N. 2011. “Cognitive Behavioral Therapy for the Treatment of Post‐Traumatic Stress Disorder: A Review.” Neuropsychiatric Disease and Treatment 7, no. 1: 167–181. 10.2147/ndt.s10389. 21552319 PMC3083990

[cpp70114-bib-0070] Khaleghipour, S. , P. Gojani , M. Masjedi , and E. Behzadi . 2017. “Effects of the Schema Therapy and Mindfulness on the Maladaptive Schemas Hold by the Psoriasis Patients With the Psychopathology Symptoms.” Advanced Biomedical Research 6, no. 1: 4. 10.4103/2277-9175.190988. 28217649 PMC5309440

[cpp70114-bib-0071] Khosravi, Z. , A. Attari , and S. Rezaei . 2011. “Intimate Partner Violence in Relation to Early Maladaptive Schemas in a Group of Outpatient Iranian Women.” Procedia ‐ Social and Behavioral Sciences 30: 1374–1377. 10.1016/j.sbspro.2011.10.266.

[cpp70114-bib-0072] Klein, W. , S. Li , and S. Wood . 2023. “A Qualitative Analysis of Gaslighting in Romantic Relationships.” Personal Relationships 30, no. 4: 1316–1340. 10.1111/pere.12510.

[cpp70114-bib-0073] Kliethermes, M. D. , K. Drewry , and R. A. Wamser . 2024. “Trauma‐Focused Cognitive‐Behavioral Therapy.” In Evidence‐Based Treatments for Trauma‐Related Disorders in Children and Adolescents, edited by M. A. Landolt , M. Cloitre , and U. Schnyder . Springer. 10.1007/978-3-031-77215-3_8.

[cpp70114-bib-0074] Körük, S. 2017. “Early Maladaptive Schemas and Attachment Styles Predicting Tendencies in Intimate Relationship.” European Journal of Education Studies 3, no. 9: 393–411. 10.5281/zenodo.889032.

[cpp70114-bib-0076] Lobbestael, J. , M. Van Vreeswijk , and A. Arntz . 2007. “Shedding Light on Schema Modes: A Clarification of the Mode Concept and Its Current Research Status.” Netherlands Journal of Psychology 63, no. 3: 69–78. 10.1007/bf03061068.

[cpp70114-bib-0077] Louis, J. M. , and M. E. Reyes . 2023. “Parental Intimate Partner Violence and Demographic Variables Predict Self‐Esteem Among Adolescents in India During COVID‐19.” Journal of Asian and African Studies 60, no. 1: 255–264. 10.1177/00219096231168063.

[cpp70114-bib-0078] Louis, J. P. , G. Lockwood , and K. M. Louis . 2024. “A Model of Core Emotional Needs and Toxic Experiences: Their Links With Schema Domains, Well‐Being, and Ill‐Being.” Behavioral Science 14, no. 6: 443. 10.3390/bs14060443. PMC11200969 38920775

[cpp70114-bib-0079] Lumley, M. N. , and K. L. Harkness . 2007. “Specificity in the Relations Among Childhood Adversity, Early Maladaptive Schemas, and Symptom Profiles in Adolescent Depression.” Cognitive Therapy and Research 31, no. 5: 639–657. 10.1007/s10608-006-9100-3.

[cpp70114-bib-0080] Lynch, S. M. 2013. “Not Good Enough and on a Tether: Exploring How Violent Relationships Impact Women's Sense of Self.” Psychodynamic Psychiatry 41, no. 2: 219–246. 10.1521/pdps.2013.41.2.219. 23713619

[cpp70114-bib-0081] Mander, J. V. , G. A. Jacob , L. Götz , I. Sammet , S. Zipfel , and M. Teufel . 2014. “Associations Between Grawe's General Mechanisms of Change and Young's Early Maladaptive Schemas in Psychotherapy Research: A Comparative Study of Change Processes.” Psychotherapy Research 25, no. 2: 249–262. 10.1080/10503307.2014.889330. 24564413

[cpp70114-bib-0082] Mason, O. , H. Platts , and M. Tyson . 2005. “Early Maladaptive Schemas and Adult Attachment in a UK Clinical Population.” Psychology and Psychotherapy: Theory, Research and Practice 78, no. 4: 549–564. 10.1348/147608305x41371. 16354444

[cpp70114-bib-0083] Matusiewicz, A. K. , C. J. Hopwood , A. N. Banducci , and C. W. Lejuez . 2010. “The Effectiveness of Cognitive Behavioral Therapy for Personality Disorders.” Psychiatric Clinics of North America 33, no. 3: 657–685. 10.1016/j.psc.2010.04.007. 20599139 PMC3138327

[cpp70114-bib-0084] McCarthy, M. C. , and M. N. Lumley . 2012. “Sources of Emotional Maltreatment and the Differential Development of Unconditional and Conditional Schemas.” Cognitive Behaviour Therapy 41, no. 4: 288–297. 10.1080/16506073.2012.676669. 22471813

[cpp70114-bib-0085] Melamed, D. M. , J. Botting , K. Lofthouse , L. Pass , and R. Meiser‐Stedman . 2024. “The Relationship Between Negative Self‐Concept, Trauma, and Maltreatment in Children and Adolescents: A Meta‐Analysis.” Clinical Child and Family Psychology Review 27, no. 1: 220–234. 10.1007/s10567-024-00472-9. 38386241 PMC10920440

[cpp70114-bib-0086] Messman‐Moore, T. L. , and A. A. Coates . 2007. “The Impact of Childhood Psychological Abuse on Adult Interpersonal Conflict.” Journal of Emotional Abuse 7, no. 2: 75–92. 10.1300/j135v07n02_05.

[cpp70114-bib-0087] Mikulincer, M. , and P. R. Shaver . 2016. Attachment in Adulthood: Structure, Dynamics, and Change. 2nd ed. Guilford Publications.

[cpp70114-bib-0088] Mizara, A. , L. Papadopoulos , and S. McBride . 2012. “Core Beliefs and Psychological Distress in Patients With Psoriasis and Atopic Eczema Attending Secondary Care: The Role of Schemas in Chronic Skin Disease.” British Journal of Dermatology 166, no. 5: 986–993. 10.1111/j.1365-2133.2011.10799.x. 22211355

[cpp70114-bib-0089] Mojallal, M. , R. M. Simons , and J. S. Simons . 2021. “Childhood Maltreatment and Adulthood Proneness to Shame and Guilt: The Mediating Role of Maladaptive Schemas.” Motivation and Emotion 45, no. 2: 197–210. 10.1007/s11031-021-09866-6.

[cpp70114-bib-0090] Molendijk, T. 2023. “Moral Coping or Simply Uncomplicated Soldiering? How Soldiers Avoid Moral Injury Through Simplification, Justification, Rationalization, and Compartmentalization.” Armed Forces & Society 50, no. 4: 977–999. 10.1177/0095327x231165910.

[cpp70114-bib-0091] Moola, S. , Z. Munn , C. Tufanaru , et al. 2020. “Chapter 7: Systematic Reviews of Etiology and Risk.” In JBI Manual for Evidence Synthesis, edited by E. Aromataris , C. Lockwood , K. Porritt , B. Pilla , and Z. Jordan . JBI; 2024. https://synthesismanual.jbi.global.10.46658/JBIMES‐24‐0610.46658/JBIMES‐24‐06.

[cpp70114-bib-0092] Muris, P. 2006. “Maladaptive Schemas in Non‐Clinical Adolescents: Relations to Perceived Parental Rearing Behaviours, Big Five Personality Factors and Psychopathological Symptoms.” Clinical Psychology & Psychotherapy 13: 405–413. 10.1002/cpp.506.

[cpp70114-bib-0093] Nagy, L. M. , K. E. Polk , and E. Muckerheide . 2023. “Self‐Criticism in Anger, Aggression, and Violence.” In Handbook of Anger, Aggression, and Violence, edited by C. R. Martin , V. R. Preedy , and V. B. Patel , 695–707. Springer. 10.1007/978-3-031-31547-3_167.

[cpp70114-bib-0094] Naismith, I. , K. Ripoll‐Nuñez , and G. B. Henao . 2022. “Depression, Anxiety, and Posttraumatic Stress Disorder Following Intimate Partner Violence: The Role of Self‐Criticism, Guilt, and Gender Beliefs.” Violence Against Women 30, no. 3–4: 791–811. 10.1177/10778012221142917. 36482687

[cpp70114-bib-0095] Nicol, A. , A. S. Mak , K. Murray , I. Walker , and D. Buckmaster . 2020. “The Relationships Between Early Maladaptive Schemas and Youth Mental Health: A Systematic Review.” Cognitive Therapy and Research 44, no. 4: 715–751. 10.1007/s10608-020-10092-6.

[cpp70114-bib-0096] Wright, M. O. D. , E. Crawford , and D. Del Castillo . 2009. “Childhood Emotional Maltreatment and Later Psychological Distress Among College Students: The Mediating Role of Maladaptive Schemas.” Child Abuse & Neglect 33, no. 1: 59–68. 10.1016/j.chiabu.2008.12.007. 19167067

[cpp70114-bib-0097] Obeid, S. , H. Sacre , C. Haddad , et al. 2019. “Factors Associated With Fear of Intimacy Among a Representative Sample of the Lebanese Population: The Role of Depression, Social Phobia, Self‐Esteem, Intimate Partner Violence, Attachment, and Maladaptive Schemas.” Perspectives in Psychiatric Care 56, no. 3: 486–494. 10.1111/ppc.12438. 31549436

[cpp70114-bib-0098] Ozdemir, N. , and S. Sahin . 2020. “The Impact of Childhood Traumatic Experiences on Self‐Esteem and Interpersonal Relationships.” Psychiatry and Behavioral Sciences 10, no. 4: 185. 10.5455/pbs.20200502025907.

[cpp70114-bib-0099] Page, M. J. , J. E. McKenzie , P. M. Bossuyt , et al. 2021. “The PRISMA 2020 Statement: An Updated Guideline for Reporting Systematic Reviews.” BMJ (Clinical Research ed.) 372: 71. 10.1136/bmj.n71. PMC8005924 33782057

[cpp70114-bib-0100] Paim, K. C. , and D. Falcke . 2018. “The Experiences in the Family of Origin and the Early Maladaptive Schemas as Predictors of Marital Violence in Men and Women.” Análise Psicológica 36, no. 3: 279–293. 10.14417/ap.1242.

[cpp70114-bib-0101] Perizzolo Pointet, V. C. , D. A. Moser , M. Vital , and A. Todorov . 2021. “Violence Exposure Is Associated With Atypical Appraisal of Threat Among Women: An EEG Study.” Frontiers in Psychology 11: 576852. 10.3389/fpsyg.2020.576852. 33510667 PMC7835125

[cpp70114-bib-0102] Petrocelli, J. V. , B. A. Glaser , G. B. Calhoun , and L. F. Campbell . 2001. “Early Maladaptive Schemas of Personality Disorder Subtypes.” Journal of Personality Disorders 15, no. 6: 546–559. 10.1521/pedi.15.6.546.19189. 11778396

[cpp70114-bib-0103] Pietri, M. , and A. Bonnet . 2017. “Analysis of Early Representations and Personality Among Victims of Domestic Violence.” European Review of Applied Psychology 67, no. 4: 199–206. 10.1016/j.erap.2017.04.001.

[cpp70114-bib-0104] Pilkington, P. D. , A. Bishop , and R. Younan . 2020. “Adverse Childhood Experiences and Early Maladaptive Schemas in Adulthood: A Systematic Review and Meta‐Analysis.” Clinical Psychology & Psychotherapy 28, no. 3: 569–584. 10.1002/cpp.2533. 33270299

[cpp70114-bib-0105] Prince, R. K. 2009. “Early Maladaptive Schemas: The Role of Gender.” Theses Digitization Project. 3714.

[cpp70114-bib-0106] Pugliese, E. , O. Mosca , A. M. Saliani , et al. 2023b. “Pathological Affective Dependence (PAD) as an Antecedent of Intimate Partner Violence (IPV): A Pilot Study of PAD's Cognitive Model on a Sample of IPV Victims.” Psychology 14: 305–333. 10.4236/psych.2023.142018.

[cpp70114-bib-0107] Pugliese, E. , A. M. Saliani , O. Mosca , F. Maricchiolo , and F. Mancini . 2023a. “When the War Is in Your Room: A Cognitive Model of Pathological Affective Dependence (PAD) and Intimate Partner Violence (IPV).” Sustainability 15, no. 2: 1624. 10.3390/su15021624.

[cpp70114-bib-0108] Renner, F. , J. Lobbestael , F. Peeters , A. Arntz , and M. Huibers . 2012. “Early Maladaptive Schemas in Depressed Patients: Stability and Relation With Depressive Symptoms Over the Course of Treatment.” Journal of Affective Disorders 136, no. 3: 581–590. 10.1016/j.jad.2011.10.027. 22119093

[cpp70114-bib-0109] Roemmele, M. , and T. L. Messman‐Moore . 2011. “Child Abuse, Early Maladaptive Schemas, and Risky Sexual Behavior in College Women.” Journal of Child Sexual Abuse 20, no. 3: 264–283. 10.1080/10538712.2011.575445. 21660814

[cpp70114-bib-0110] Roman, N. V. , and J. Ryan . 2021. Family Violence: Prevalence, Risk Factors and Perspective. Nova Science Publishers.

[cpp70114-bib-0111] Rowan, A. B. , and D. W. Foy . 1993. “Post‐Traumatic Stress Disorder in Child Sexual Abuse Survivors: A Literature Review.” Journal of Traumatic Stress 6, no. 1: 3–20. 10.1007/bf02093359.

[cpp70114-bib-0112] Schulte, E. M. , C. Bach , R. I. Berkowitz , J. D. Latner , and R. L. Pearl . 2021. “Adverse Childhood Experiences and Weight Stigma: Co‐Occurrence and Associations With Psychological Well‐Being.” Stigma and Health 6, no. 4: 408–418. 10.1037/sah0000341. 34926807 PMC8675894

[cpp70114-bib-0113] Seligman, M. E. P. 1975. Helplessness: On Depression, Development, and Death. W H Freeman/Times Books/Henry Holt & Co.

[cpp70114-bib-0114] Senkans, S. , T. E. McEwan , and J. R. Ogloff . 2020. “Conceptualising Intimate Partner Violence Perpetrators' Cognition as Aggressive Relational Schemas.” Aggression and Violent Behavior 55: 101456. 10.1016/j.avb.2020.101456.

[cpp70114-bib-0115] Sharhabani‐Arzy, R. , M. Amir , and A. Swisa . 2005. “Self‐Criticism, Dependency and Posttraumatic Stress Disorder Among a Female Group of Help‐Seeking Victims of Domestic Violence in Israel.” Personality and Individual Differences 38, no. 5: 1231–1240. 10.1016/j.paid.2004.08.006.

[cpp70114-bib-0116] Silvestri, V. , S. Gobbo , E. Pugliese , F. Mancini , and F. Visco‐Comandini . 2025. “The Perception of Trustworthiness and Emotional Identification in Women Experiencing Intimate Partner Violence: A Behavioral Pilot Study.” Brain Sciences 15: 429. 10.3390/brainsci15050429. 40426600 PMC12110009

[cpp70114-bib-0117] Sójta, K. , A. Margulska , W. Jóźwiak‐Majchrzak , A. Grażka , K. Grzelczak , and D. Strzelecki . 2023. “Cognitive–Affective Risk Factors of Female Intimate Partner Violence Victimization: The Role of Early Maladaptive Schemas and Strategic Emotional Intelligence.” Brain Sciences 13, no. 7: 1118. 10.3390/brainsci13071118. 37509048 PMC10377412

[cpp70114-bib-0118] Storm‐Mathisen, F. 2024. “‘Violence Is Completely Normal’: Managing Violence Through Narrative Normalization.” British Journal of Criminology 65, no. 1: 37–53. 10.1093/bjc/azae030.

[cpp70114-bib-0119] Supyan, V. 2022. “American Model of Capitalism: Advantages and Challenges of 21st Century.” World Economy and International Relations 66, no. 9: 90–97. 10.20542/0131-2227-2022-66-9-90-97.

[cpp70114-bib-0120] Taccini, F. , A. A. Rossi , and S. Mannarini . 2024. “Unveiling the Role of Emotion Regulation in the Relationship Between Intimate Partner Violence Increases and Post‐Traumatic Stress Disorder: A Mediation Analysis.” Behavioral Science 14, no. 9: 799. 10.3390/bs14090799. PMC11429198 39336014

[cpp70114-bib-0121] Tan, R. H. , S. S. Chang , W. L. Teh , N. Chandwani , M. Subramaniam , and J. Liu . 2025. “Social Support and Emotion Dysregulation: A Serial Pathway From Child Maltreatment to Depressive Symptoms in Adults With Affective Disorders.” European Journal of Psychiatry 39, no. 2: 100286. 10.1016/j.ejpsy.2024.100286.

[cpp70114-bib-0122] Tariq, A. , C. Reid , and S. W. Chan . 2021. “A Meta‐Analysis of the Relationship Between Early Maladaptive Schemas and Depression in Adolescence and Young Adulthood.” Psychological Medicine 51, no. 8: 1233–1248. 10.1017/s0033291721001458. 34109934

[cpp70114-bib-0123] Taşkale, N. , and G. Soygüt . 2016. “Risk Factors for Women's Intimate Partner Violence Victimization: An Examination From the Perspective of the Schema Therapy Model.” Journal of Family Violence 32, no. 1: 3–12. 10.1007/s10896-016-9855-6.

[cpp70114-bib-0124] Taylor, C. D. , P. Bee , and G. Haddock . 2016. “Does Schema Therapy Change Schemas and Symptoms? A Systematic Review Across Mental Health Disorders.” Psychology and Psychotherapy: Theory, Research and Practice 90, no. 3: 456–479. 10.1111/papt.12112. PMC5573974 28035734

[cpp70114-bib-0125] Tenore, K. , B. Basile , T. Cosentino , et al. 2020. “Imagery Rescripting on Guilt‐Inducing Memories in OCD: A Single Case Series Study.” Frontiers in Psychiatry 11: 543806. 10.3389/fpsyt.2020.543806. 33192658 PMC7554624

[cpp70114-bib-0126] Tenore, K. , A. Mancini , O. I. Luppino , and F. Mancini . 2022. “Group Imagery Rescripting on Childhood Memories Delivered via Telehealth: A Preliminary Study.” Frontiers in Psychiatry 13: 862289. 10.3389/fpsyt.2022.862289. 35815039 PMC9263974

[cpp70114-bib-0127] Tomkins, J. , A. D. Simpson , and D. L. Polaschek 2023. “High‐Risk Victims of Intimate Partner Violence: An Examination of Abuse Characteristics, Psychosocial Vulnerabilities and Reported Revictimization.” CrimRxiv. 10.21428/cb6ab371.1845686a.

[cpp70114-bib-0128] Torres, A. , R. Mendes , and T. Lapa . 2008. “Families in Europe.” Portuguese Journal of Social Science 7, no. 1: 49–84. 10.1386/pjss.6.2.97_1.

[cpp70114-bib-0129] Turner, H. M. , K. S. Rose , and M. J. Cooper . 2005. “Schema and Parental Bonding in Overweight and Nonoverweight Female Adolescents.” International Journal of Obesity 29: 381–387. 10.1038/sj.ijo.0802915. 15768044

[cpp70114-bib-0130] Upenieks, L. , and J. Ford‐Robertson . 2022. “Childhood Abuse, Goal‐Striving Stress and Self‐Esteem: An Explanatory Role for Perceptions of Divine Control?” Journal of Religion and Health 62, no. 2: 906–931. 10.1007/s10943-022-01682-7. 36205838

[cpp70114-bib-0131] Viechtbauer, W. 2010. “Conducting Meta‐Analyses in R With the Metafor Package.” Journal of Statistical Software 36, no. 3: 1–48. 10.18637/jss.v036.i03.

[cpp70114-bib-0132] von Wendorff, C. , D. Bürgin , M. Meier , et al. 2025. “Psychological Resilience and Childhood Maltreatment: The Role of Self‐Efficacy, Personality Functioning and Social Support in Young Adult Residential Care Leavers.” Child Abuse & Neglect 163: 107317. 10.1016/j.chiabu.2025.107317. 39977962

[cpp70114-bib-0133] Walker, H. E. , J. S. Freud , R. A. Ellis , S. M. Fraine , and L. C. Wilson . 2017. “The Prevalence of Sexual Revictimization: A Meta‐Analytic Review.” Trauma Violence Abuse 20, no. 1: 67–80. 10.1177/1524838017692364. 29333937

[cpp70114-bib-0134] Walker, L. E. 2009. The Battered Woman Syndrome. 3rd ed. Springer Publishing Company.

[cpp70114-bib-0135] Whitfield, C. L. , R. F. Anda , S. R. Dube , and V. J. Felitti . 2003. “Violent Childhood Experiences and the Risk of Intimate Partner Violence in Adults.” Journal of Interpersonal Violence 18, no. 2: 166–185. 10.1177/0886260502238733.

[cpp70114-bib-0136] Wright, N. M. , J. Dmitrieva , and A. P. DePrince . 2021. “Dependence in Adult Relationships: Latent Classes of Relational Dependence and Associated Outcomes in Women Exposed to Intimate Partner Abuse.” Psychological Trauma Theory Research Practice and Policy 13, no. 3: 359–367. 10.1037/tra0000661. 32816513

[cpp70114-bib-0137] Xie, G. , J. Chang , M. Yuan , et al. 2021. “Childhood Abuse and Borderline Personality Disorder Features in Chinese Undergraduates: The Role of Self‐Esteem and Resilience.” BMC Psychiatry 21: 326. 10.1186/s12888-021-03332-w. 34210279 PMC8252225

[cpp70114-bib-0138] Yakın, D. , and A. Arntz . 2023. “Understanding the Reparative Effects of Schema Modes: An in‐Depth Analysis of the Healthy Adult Mode.” Frontiers in Psychiatry 14: 1204177. 10.3389/fpsyt.2023.1204177. 37941965 PMC10628052

[cpp70114-bib-0139] Yan, E. , and T. Karatzias . 2016. “Childhood Abuse and Current Intimate Partner Violence: A Population Study in Hong Kong.” Journal of Interpersonal Violence 35, no. 1–2: 233–251. 10.1177/0886260516682521. 27940606

[cpp70114-bib-0140] Young, J. E. 1990. Cognitive Therapy for Personality Disorders: A Schema‐Focused Approach. Professional Resource Exchange, Inc.

[cpp70114-bib-0141] Young, J. E. 1999. Cognitive Therapy for Personality Disorders: A Schema‐Focused Approach. 3rd ed. Professional Resource Press/Professional Resource Exchange.

[cpp70114-bib-0142] Young, J. E. 2005. Young Schema Questionnaire‐Long Form 3 (YSQ‐L3). Schema Therapy Institute.

[cpp70114-bib-0143] Young, J. E. , and G. Brown . 2005. “Young Schema Questionnaire‐Short Form; Version 3 (YSQ‐SB, YSQ).” APA PsycTests. 10.1037/t67023-000.

[cpp70114-bib-0144] Young, J. E. , and C. Flanagan . 1998. “Schema‐Focused Therapy for Narcissistic Patients.” In Disorders of Narcissism: Diagnostic, Clinical, and Empirical Implications, edited by E. F. Ronningstam and E. F. Ronningstam , 239–262. American Psychiatric Association.

[cpp70114-bib-0145] Young, J. E. , J. S. Klosko , and M. E. Weishaar . 2003. Schema Therapy: A Practitioner's Guide. Guilford Press.

[cpp70114-bib-0146] Zapcic, I. , M. Fabbri , and S. Karandikar . 2023. “‘How Can I Love You if You Don't Let Me Do This?’ Evaluating the Effects of the Red Pill Seduction Community Experienced by Intimate Partners.” Journal of Aggression, Maltreatment & Trauma 33, no. 3: 273–290. 10.1080/10926771.2023.2186302.

[cpp70114-bib-0147] Zirakbash, A. , F. Naderi , and M. Enayati . 2015. “One of Early Maladaptive Schemas' Causal Relationship Through Metacognitive Beliefs With Borderline and Antisocial Personality Patterns.” Journal of Education Health Promotion 4, no. 1: 62. 10.4103/2277-9531.162382. 26430689 PMC4579759

[cpp70114-bib-0148] Zufferey, C. 2022. “The Safety of Home: Violence Against Women.” In The Complexities of Home in Social Work, 1st ed., 98–110. Routledge. 10.4324/9781003032489-9.

