# Supplementary material for: When Adverse Experiences Influence the Interpretation of Ourselves, Others and the World: A Systematic Review and Meta‐Analysis of Maladaptive Schemas in Victims of Violence
Source: Clin Psychol Psychother. 2025 Jul 15;32(4):e70114. doi: 10.1002/cpp.70114 (PMC12264405; doi:10.1002/cpp.70114)
Supplement: Supplementary file 1 — Data S1. Qualitative Results Data S2. Funnel Plot of Self‐Sacrifice Data S3. Funnel Plot of Unrelenting Standards Data S4. Funnel PlotAbuse Data S5. Funnel Plot Abandonment Data S6. Funnel Plot Dependence Data S7. Funnel Plot Vulnerability to Harm Data S8. Results of the moderation analysis in victims’ schemas Data S9. Scatter Plot of the moderation effect of age on Self‐Sacrifice Data S10. Scatter Plot of the moderation effect of age on Unrelenting Standards Data S11. Scatter Plot of the moderation effect of country on Unrelenting Standards Data S12. Scatter Plot of the moderation effect of age on Dependence Data S13. Scatter Plot of the moderation effect of country on Dependence Data S14. Scatter Plot of the moderation effect of age on Vulnerability to Harm Data S15. Scatter Plot of the moderation effect of age on Emotional Deprivation Data S16. Scatter Plot of the moderation effect of gender on Emotional Deprivation Data S17. Scatter Plot of the moderation effect of country on Emotional Deprivation Data S18. Forest Plot of Emotional Deprivation in psychological abuse victims Data S19. Funnel Plot of Emotional Deprivation in psychological abuse victims Data S20. Forest Plot of Abuse in psychological abuse victims Data S21. Funnel Plot of Abuse in psychological abuse victims Data S22. Forest Plot of Social Isolation in psychological abuse victims Data S23. Funnel Plot of Social Isolation in psychological abuse victims Data S24. Forest Plot of Failure in psychological abuse victims. Data S25. Funnel Plot of Failure in psychological abuse victims Data S26. Forest Plot of Abandonment in psychological abuse victims Data S27. Funnel Plot of Abandonment in psychological abuse victims Data S28. Forest Plot of Emotional Inhibition in psychological abuse victims Data S29. Funnel Plot of Emotional Inhibition in psychological abuse victims Data S30. Forest Plot of Vulnerability to Harm in psychological abuse victims Data S31. Funnel Plot of Vulnerability to Harm in psychologi [file CPP-32-e70114-s001.docx]

**When adverse experiences influence the interpretation of ourselves, others, and the world: a systematic review and meta-analysis of maladaptive schemas in victims of violence**

*Allison Uvelli^^[[1]](#footnote-1)^,^[[2]](#footnote-2)^*^, Marta Floridi^1,2^, Giuseppe Agrusti^2^, Anna Chiara Franquillo^2^, Lucia Fiumalbi^2^, Tommaso Micheloni^2^, Andreina Arcuri^2^, Stefania Iazzetta^2^, Andrea Gragnani^2,^[[3]](#footnote-3)^^*

Journal: Clinical Psychology and Psychotherapy

**Supplementary Materials**

**S1. Qualitative Results**

The data from the studies primarily focus on two macro-categories of abuse: Childhood Abuse and Intimate Partner Violence (IPV). Childhood Abuse is reported in 75% of the studies (Celsi et al., 2021; Pietri & Bonnet, 2017; Roemmele & Messman-Moore, 2011; O’Dougherty et al., 2009; Mojallal et al., 2021; Harding et al., 2012; Estevez et al., 2015; Estevez et al., 2016; Messman-Moore & Coates, 2007; McCarthy & Lumley, 2012; Crawford & O’Dougherty Wright, 2007; Fernando et al., 2024; Turner et al., 2005; Muris, 2006; Lumley & Harkness, 2007), while IPV is discussed in 40% of them (Celsi et al., 2021; Pietri & Bonnet, 2017; Sojta et al., 2023; Hassija et al., 2017; Obeid et al., 2019; Crawford & O’Dougherty Wright, 2007; Taskale & Soygut, 2016; Estevez et al., 2024).

Within the category of Childhood Abuse, various subcategories are examined. Psychological abuse appears in 65% of the studies (Roemmele & Messman-Moore, 2011; O’Dougherty et al., 2009; Harding et al., 2012; Estevez et al., 2016; Messman-Moore & Coates, 2007; Crawford & O’Dougherty Wright, 2007; McCarthy & Lumley, 2012; Fernando et al., 2024; Turner et al., 2005; Muris, 2006; Lumley & Harkness, 2007), making it the most frequently reported type of childhood abuse. Physical abuse and sexual abuse are each reported in 40% of the studies (Roemmele & Messman-Moore, 2011; O’Dougherty et al., 2009; Mojallal et al., 2021; Harding et al., 2012; Estevez et al., 2016; Crawford & O’Dougherty Wright, 2007; Fernando et al., 2024; Lumley & Harkness, 2007), highlighting their prevalence as significant forms of maltreatment during childhood. Additionally, emotional neglect is examined in 35% of the studies (Celsi et al., 2021; O’Dougherty et al., 2009; Harding et al., 2012; Estevez et al., 2015; Estevez et al., 2016; Crawford & O’Dougherty Wright, 2007; Fernando et al., 2024), underscoring its impact on the development of maladaptive behaviors later in life.

The data reveal that several maladaptive schemas emerge across the studies, with varying frequencies (Fig. 2). Mistrust and abuse is the most frequently reported schema, appearing in 60% of the studies (Crawford & O’Dougherty Wright, 2007; Pietri & Bonnet, 2017; Sojta et al., 2023; Hassija et al., 2017; Obeid et al., 2019; Roemmele & Messman-Moore, 2011; Harding et al., 2012; Messman-Moore & Coates, 2007; Estevez et al., 2015, 2016, 2024; Fernando et al., 2024) and is characterized by a pervasive belief that others are untrustworthy, harmful, or exploitative. Emotional deprivation is present in 55% (Celsi et al., 2021; Pietri & Bonnet, 2017; Roemmele & Messman-Moore, 2011; Obeid et al., 2019; Harding et al., 2012; Estevez et al., 2015, 2016, 2024; Fernando et al., 2024, Turner et al., 2005; Lumley & Harkness, 2007) and self-sacrifice in 45% of the studies (Pietri & Bonnet, 2017; O’Dougherty et al., 2009; Sojta et al., 2023; Hassija et al., 2017; Harding et al., 2012; Estevez et al., 2016; Crawford & O’Dougherty-Wright, 2007; McCarthy & Lumley, 2012; Muris, 2006), involving the belief that one’s emotional needs will not be met and a tendency to prioritize the needs of others over one’s own. Abandonment, which refers to the fear of being left alone or unsupported, is reported in 30% of the studies (Roemmele & Messman-Moore, 2011; Sojta et al., 2023; Harding et al., 2012; Messman-Moore & Coates, 2007; Celsi et al., 2021; Turner et al., 2005). Defectiveness and shame (Roemmele & Messman-Moore, 2011; O’Dougherty et al., 2009; Obeid et al., 2019; Messman-Moore & Coates, 2007; Fernando et al., 2024), which refers to the negative self-beliefs and the sense of inadequacy, and subjugation (Pietri & Bonnet, 2017; Hassija et al., 2017; Estevez et al., 2024; Turner et al., 2005; Lumley & Harkness, 2007), which indicate a tendency to submit to others, are reported in 25% of the studies. Failure (Mojallal et al., 2021; Estevez et al., 2015, 2016; Lumley & Harkness, 2007), and emotional inhibition (Mojallal et al., 2021; Estevez et al., 2016; Crawford & O’Dougherty Wright, 2007; Muris, 2006), are found in 20% of the studies. These types of schemas indicate a belief in their own incompetence, and difficulty expressing emotions. Social isolation (Mojallal et al., 2021; Estevez et al., 2024; Fernando et al., 2024), vulnerability to harm (O’Dougherty et al., 2009; Estevez et al., 2016; Lumley & Harkness, 2007), unrelating standards (McCarthy & Lumley, 2012; Taskale & Soygut, 2016; Muris, 2006), and dependence (Mojallal et al., 2021; Sojta et al., 2023; Lumley & Harkness, 2007) are found in and 15% of the studies. These types of schemas respectively denote challenges in social relationships, the belief that one is vulnerable to physical or emotional harm, the belief that one must meet excessively high standards to avoid negative consequences, and indicate a reliance on others. Lastly, negativity and pessimism appear in 10% of the studies (Mojallal et al., 2021; Sojta et al., 2023), indicating a negative outlook on life.


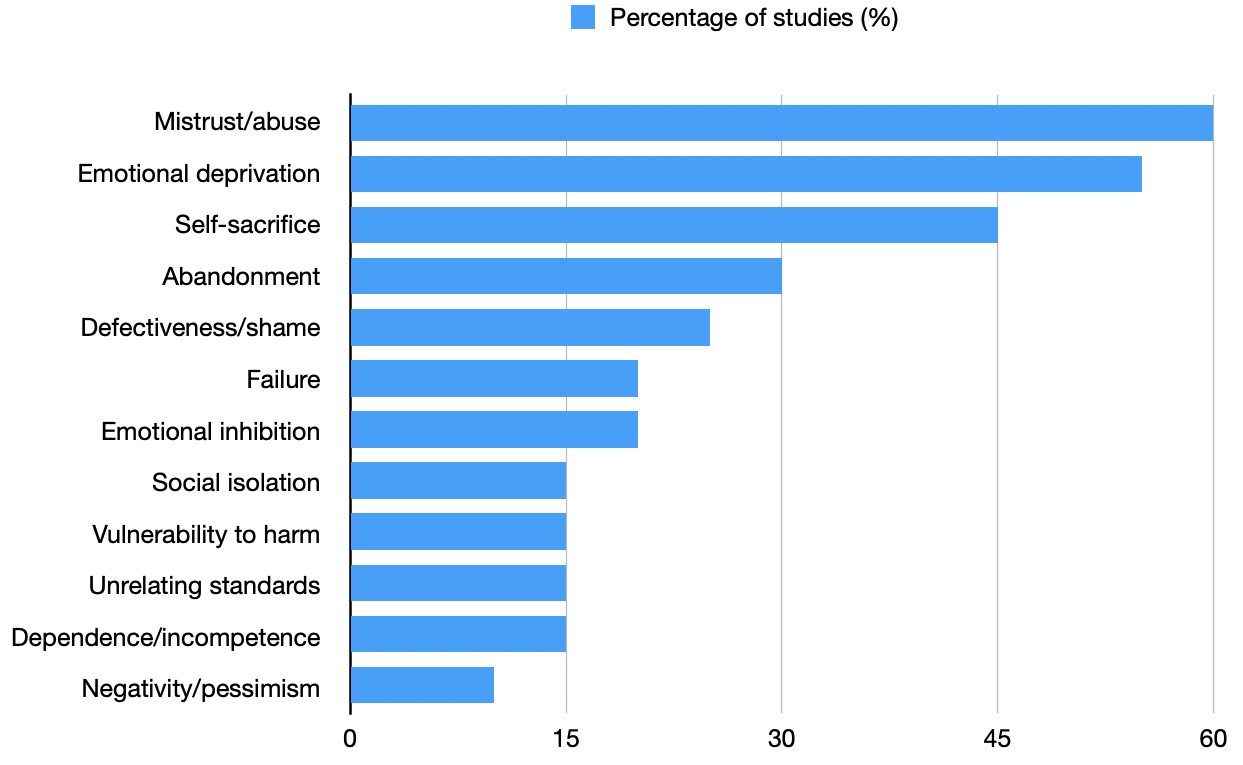


**Figure 2.** Prevalence of maladaptive schemas in studies.

The analysis of the included studies reveals distinct patterns in the prevalence of different types of abuse and their associated Early Maladaptive Schemas (EMS). Among the 20 studies reviewed, psychological abuse was the most frequently examined, appearing in 50% of the studies (Harding et al., 2012; Estevez et al., 2015, 2016; Messman-Moore & Coates, 2007; McCarthy & Lumley, 2012; Turner et al., 2005; Muris, 2006; Lumley & Harkness, 2007), followed by sexual abuse (45%) (Roemmele & Messman-Moore, 2011; O’Dougherty et al., 2009; Harding et al., 2012; Estevez et al., 2016; Fernando et al., 2024; Lumley & Harkness, 2007) and physical abuse (40%) (Roemmele & Messman-Moore, 2011; O’Dougherty et al., 2009; Estevez et al., 2016; Lumley & Harkness, 2007). Emotional neglect and intimate partner violence (IPV) were each present in 35% of the studies (O’Dougherty et al., 2009; Harding et al., 2012; Estevez et al., 2016; Fernando et al., 2024; Sojta et al., 2023; Hassija et al., 2017; Obeid et al., 2019; Estevez et al., 2024).

Regarding EMS, it is observed that emotional deprivation was the most frequently reported schema, appearing in 70% of the studies (Celsi et al., 2021; O’Dougherty et al., 2009; Harding et al., 2012; Estevez et al., 2015, 2016; Fernando et al., 2024; Turner et al., 2005; Estevez et al., 2024), followed by mistrust and abuse, which was identified in 65% of the studies (Roemmele & Messman-Moore, 2011; Pietri & Bonnet, 2017; Hassija et al., 2017; Obeid et al., 2019; Estevez et al., 2016; Sojta et al., 2023; Estevez et al., 2024). Self-sacrifice was reported in 50% of the studies (Pietri & Bonnet, 2017; O’Dougherty et al., 2009; Harding et al., 2012; Estevez et al., 2016; Hassija et al., 2017), while vulnerability to harm and subjugation each appeared in 40% of the studies (Lumley & Harkness, 2007; Estevez et al., 2016; Hassija et al., 2017; Estevez et al., 2024; Turner et al., 2005).

Distinct relationships were also observed between specific abuse and early maladaptive schemas (EMS). Results showed that sexual abuse was strongly associated with distrust and abuse (100% of relevant studies), self-sacrifice (66.7%) and emotional deprivation (50%). In comparison, physical abuse was predominantly related to vulnerability to harm and distrust (both 75%). As for psychological abuse and emotional neglect, it is observed that both are correlated with emotional deprivation (80%), distrust and abuse (70%), and self-sacrifice (60%). Looking at studies on intimate partner violence (IPV), the most associated patterns are distrust and abuse (85.7%) and self-sacrifice (71.4%). Notably, patterns of distrust and abuse appeared consistently across all types of abuse (73.7% of total studies), followed by emotional deprivation (63.2%) and self-sacrifice (57.9%), suggesting that these are core maladaptive beliefs developed in various forms of abuse. Ultimately, the results demonstrate how various adverse childhood experiences often associate with distinctive cognitive schemas that subsequently influence individuals' interpretations of themselves, others, and the world, with some schemas appearing more pervasive than others.

**S2. Funnel Plot of Self-Sacrifice
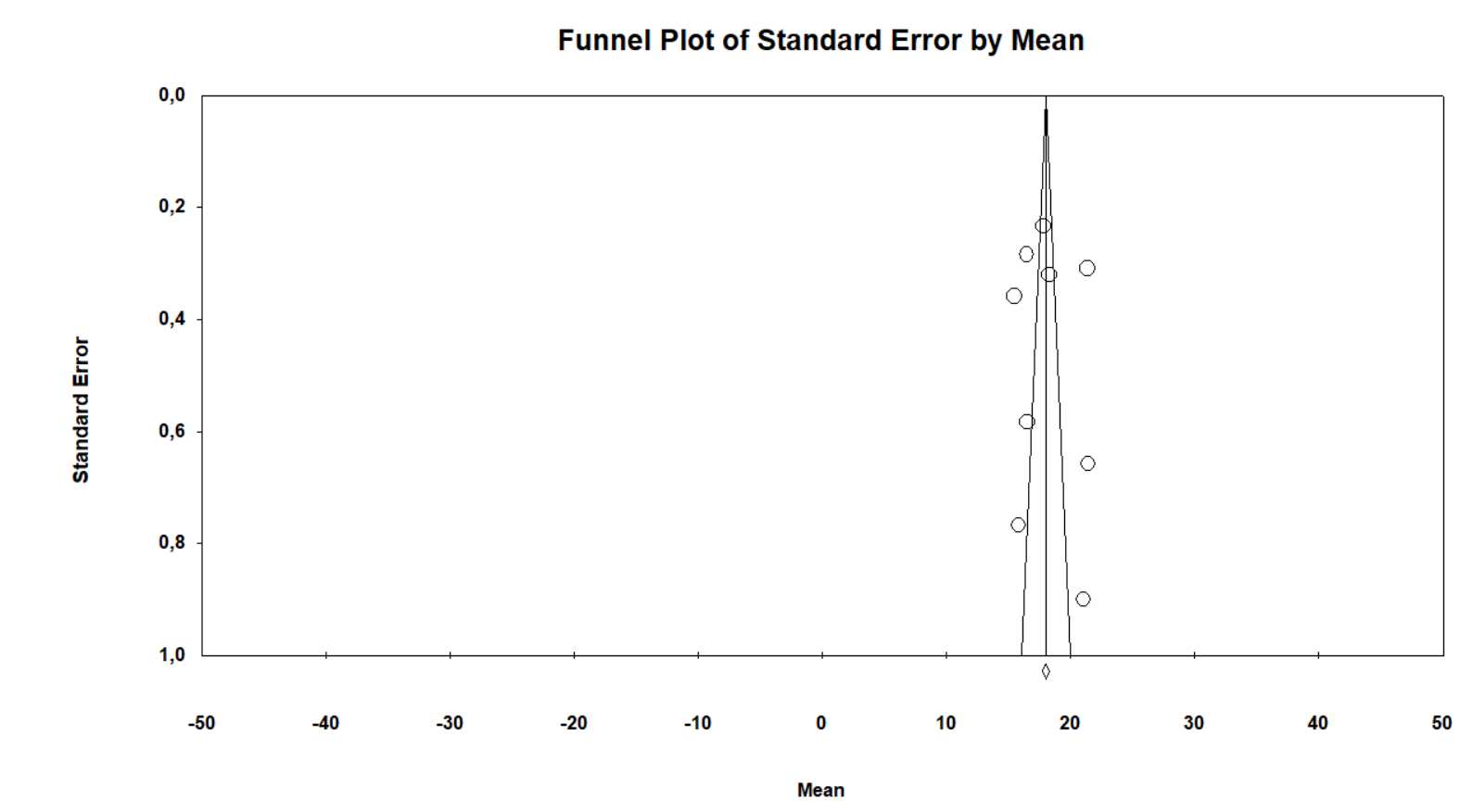
**

**S3. Funnel Plot of Unrelenting Standards**

**
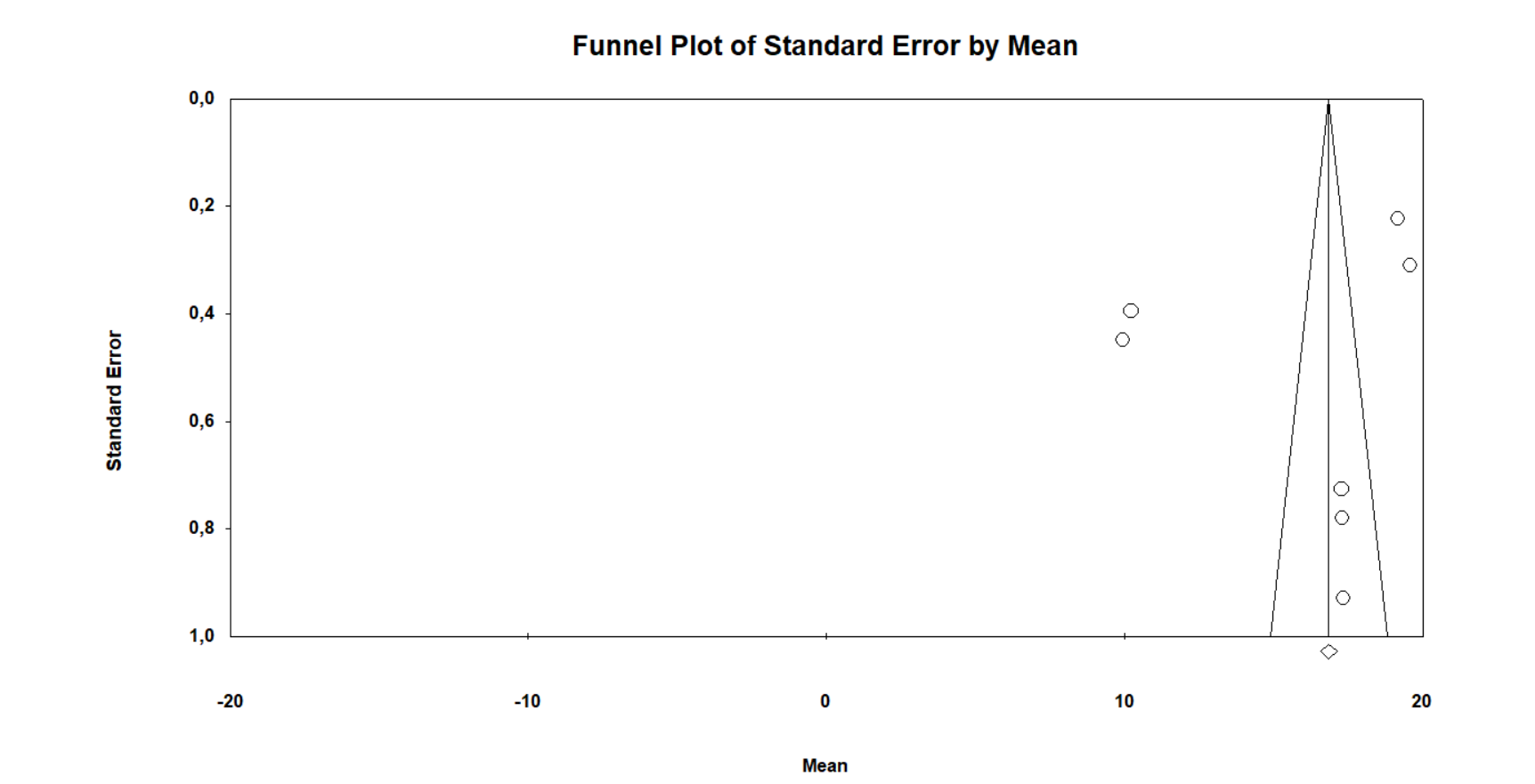
**

**S4. Funnel Plot
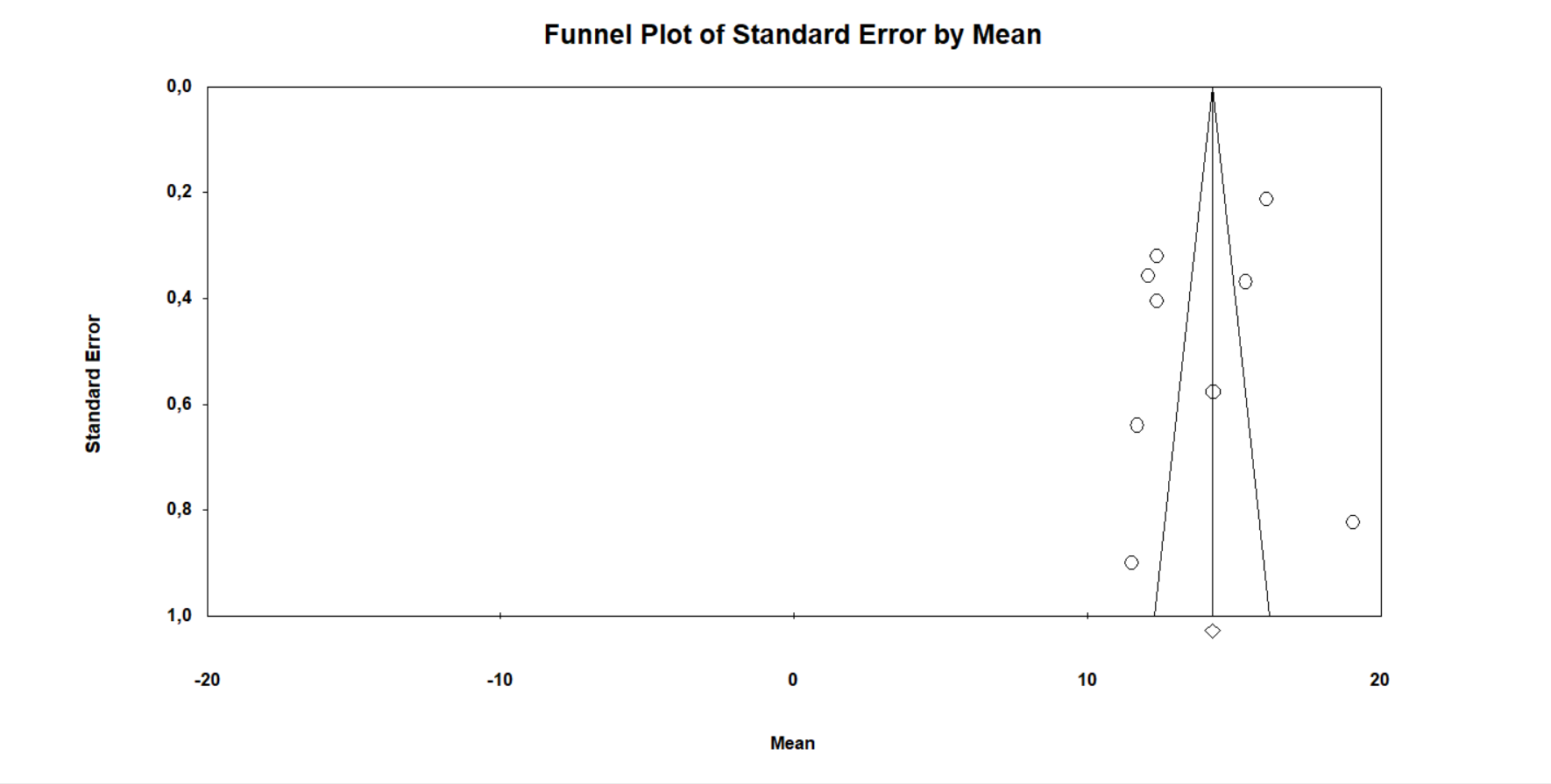
 Abuse**

**S5. Funnel Plot Abandonment**

**
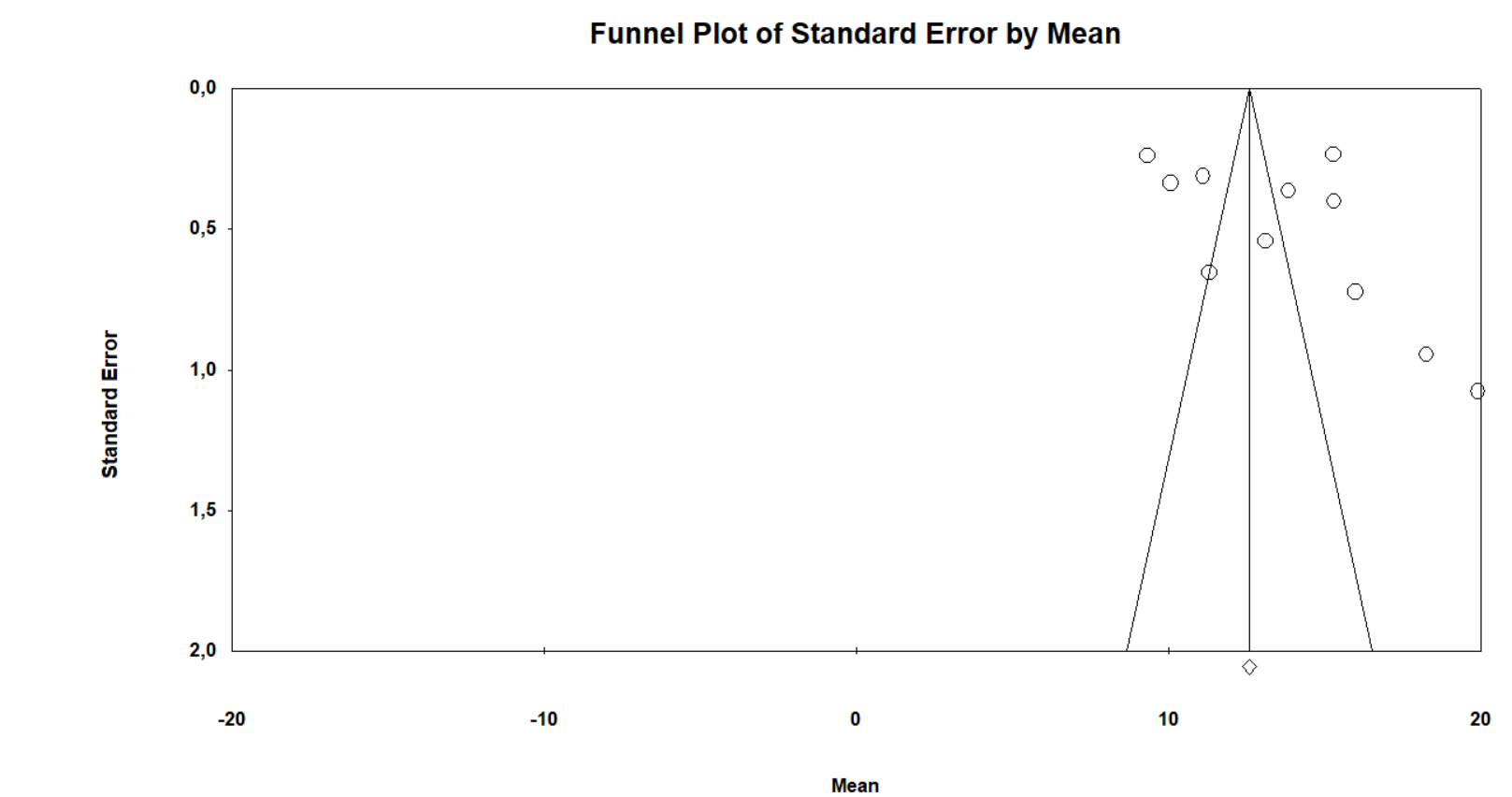
**

**S6. Funnel Plot Dependence**

**
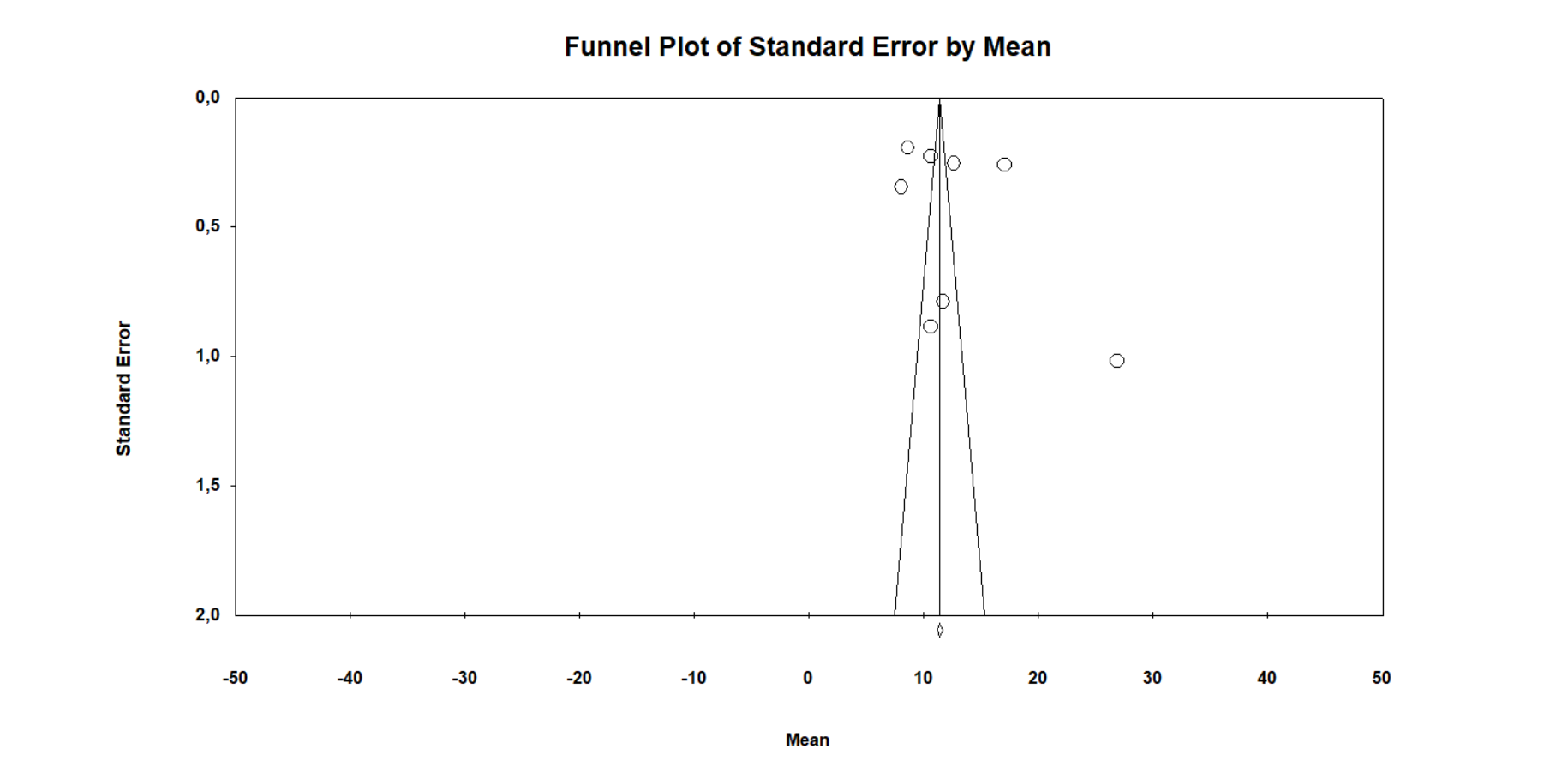
**

**S7. Funnel Plot Vulnerability to Harm
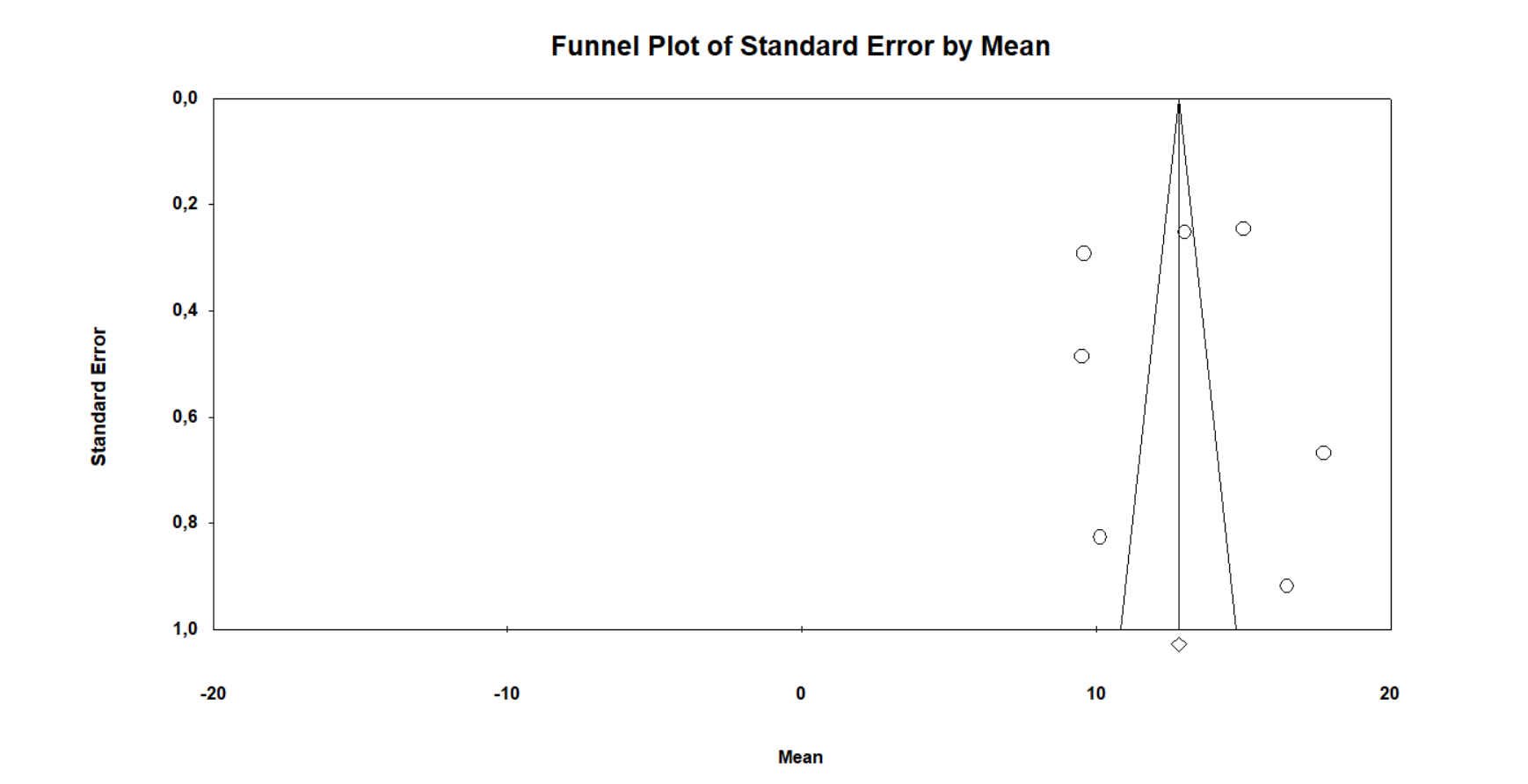
**

**S8. Results of the moderation analysis in victims’ schemas**

| **EMS** | **Moderator (age, gender, country)** | **se** | **z** | **p** |
| --- | --- | --- | --- | --- |
| *Self-sacrifice* | - 0.20 - -1.34 - 1.70 | - 0.08 - 1.96 - 1.46 | - 2.38 - -0.68 - 1.16 | - *0.01* - 0.49 - 0.24 |
| *Unrelating standards* | - -0.37 - 3.37 - -5.21 | - 0.13 - 4.16 - 1.71 | - -2.85 - 0.81 - -3.04 | - *0.004* - 0.41 - *0.002* |
| Abuse | - 0.09 - 0.96 - -0.18 | - 0.11 - 1.26 - 1.47 | - 0.80 - 0.76 - -0.12 | - 0.42 - 0.44 - 0.90 |
| Abandonment | - 0.04 - -2.61 - 3.30 | - 0.13 - 1.90 - 1.90 | - 0.34 - -1.37 - 1.73 | - 0.73 - 0.17 - 0.08 |
| *Dependence* | - 0.47 - -0.97 - 5.54 | - 0.15 - 2.82 - 2.71 | - 3.02 - -0.34 - 2.04 | - *0.002* - 0.73 - *0.04* |
| *Vulnerability to harm* | - 0.35 - -0.90 - 2.94 | - 0.13 - 2.31 - 2.57 | - 2.68 - -0.39 - 1.14 | - *0.007* - 0.69 - 0.25 |
| Social isolation | - 0.20 - -0.02 - 1.96 | - 0.15 - 2.05 - 2.24 | - 1.30 - -0.01 - 0.87 | - 0.19 - 0.98 - 0.38 |
| *Emotional deprivation* | - 0.34 - -3.93 - 4.27 | - 0.11 - 1.82 - 1.82 | - 2.95 - -2.16 - 2.35 | - *0.003* - *0.03* - *0.01* |
| Emotional inhibition | - 0.06 - 0.50 - -0.19 | - 0.08 - 1.22 - 1.15 | - 0.78 - 0.41 - -0.17 | - 0.43 - 0.67 - 0.86 |
| Failure | - 0.16 - -2.25 - 2.64 | - 0.08 - 1.73 - 1.51 | - 1.87 - -1.30 - 1.74 | - 0.06 - 0.19 - 0.08 |
| Subjugation | - 0.06 - 0.12 - 0.74 | - 0.09 - 1.43 - 1.40 | - 0.69 - 0.09 - 0.53 | - 0.48 - 0.92 - 0.59 |
| Defectiveness | - 0.13 - -1.31 - 1.69 | - 0.07 - 1.23 - 1.19 | - 1.76 - -1.06 - 1.42 | - 0.07 - 0.29 - 0.15 |
| Enmeshment | - 0.10 - 1.86 - -0.82 | - 0.20 - 2.89 - 2.95 | - 0.54 - 0.65 - 0.28 | - 0.59 - 0.51 - 0.78 |

**S9. Scatter Plot of the moderation effect of age on Self-Sacrifice
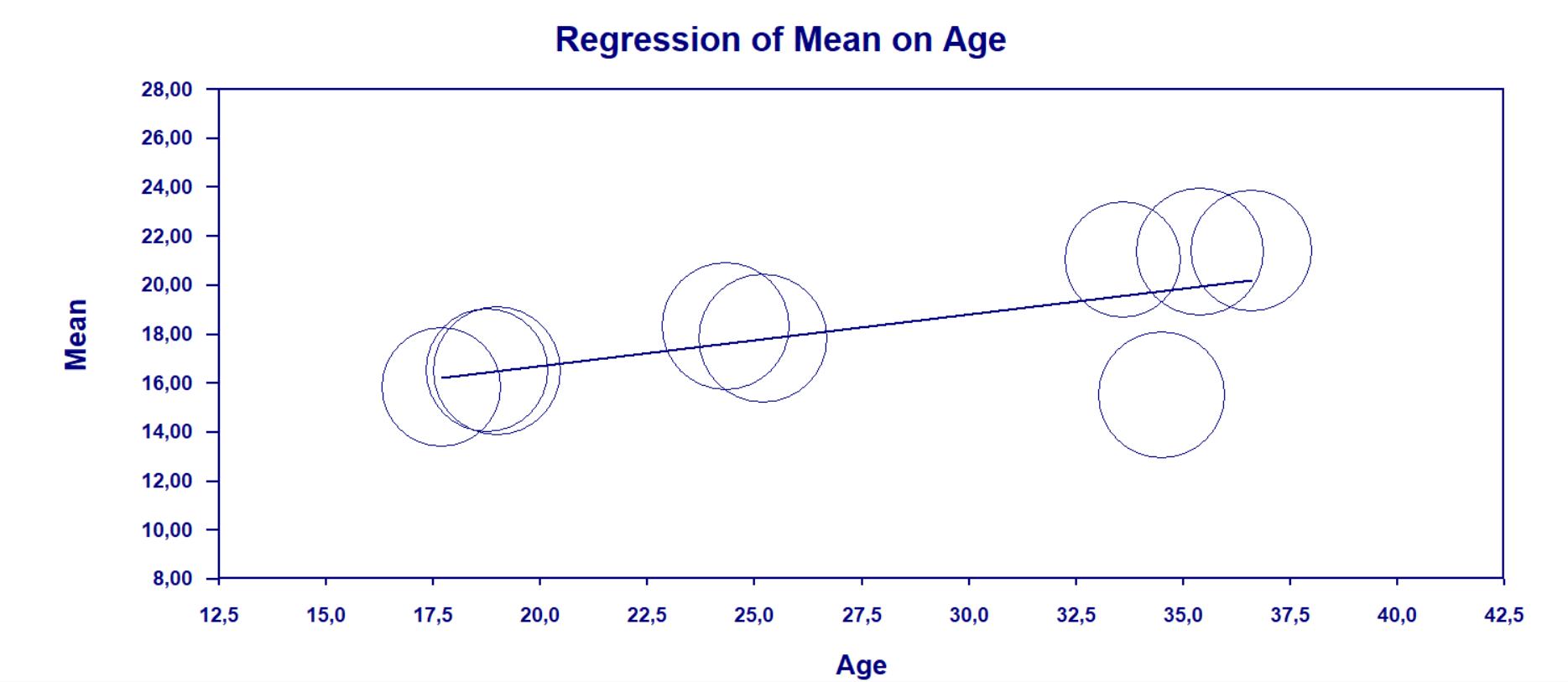
**

**S10. Scatter Plot of the moderation effect of age on Unrelenting Standards
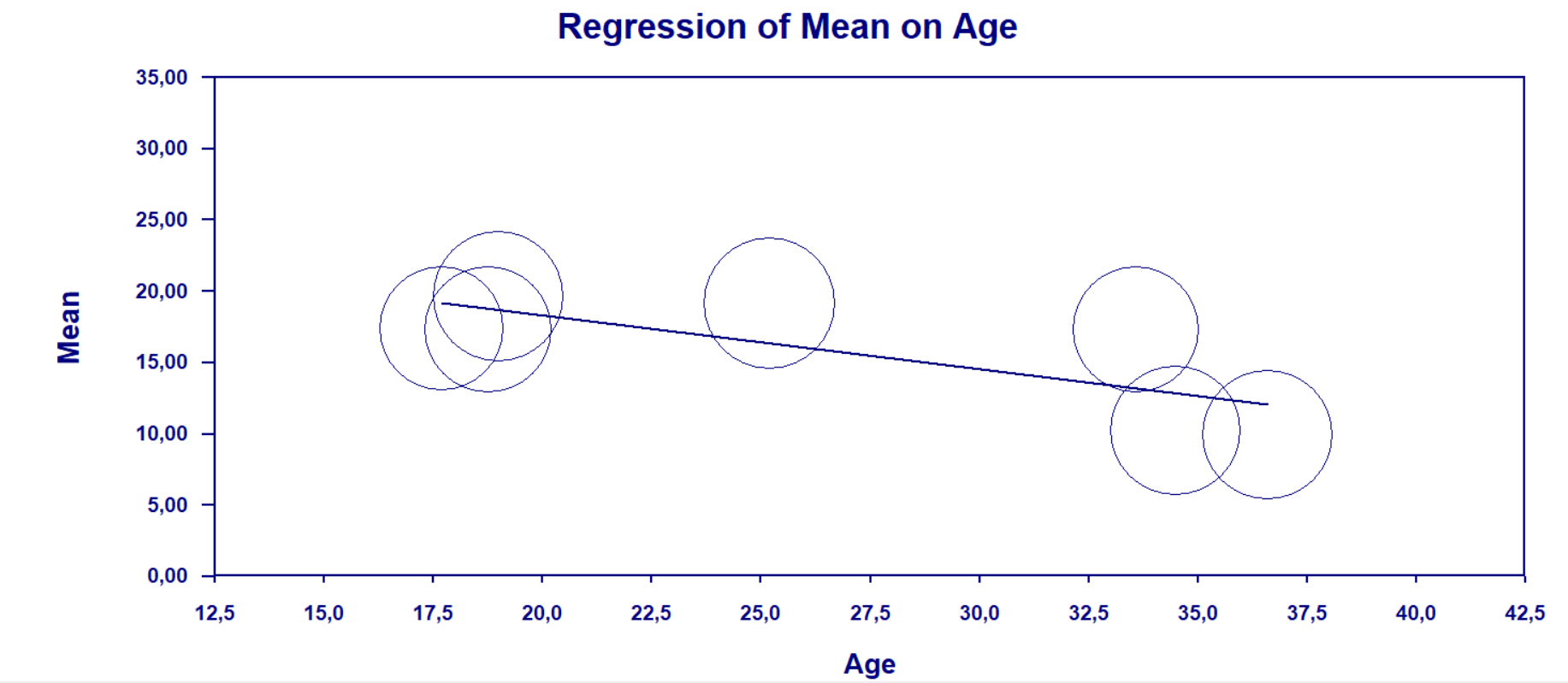
**

**S11. Scatter Plot of the moderation effect of country on Unrelenting Standards
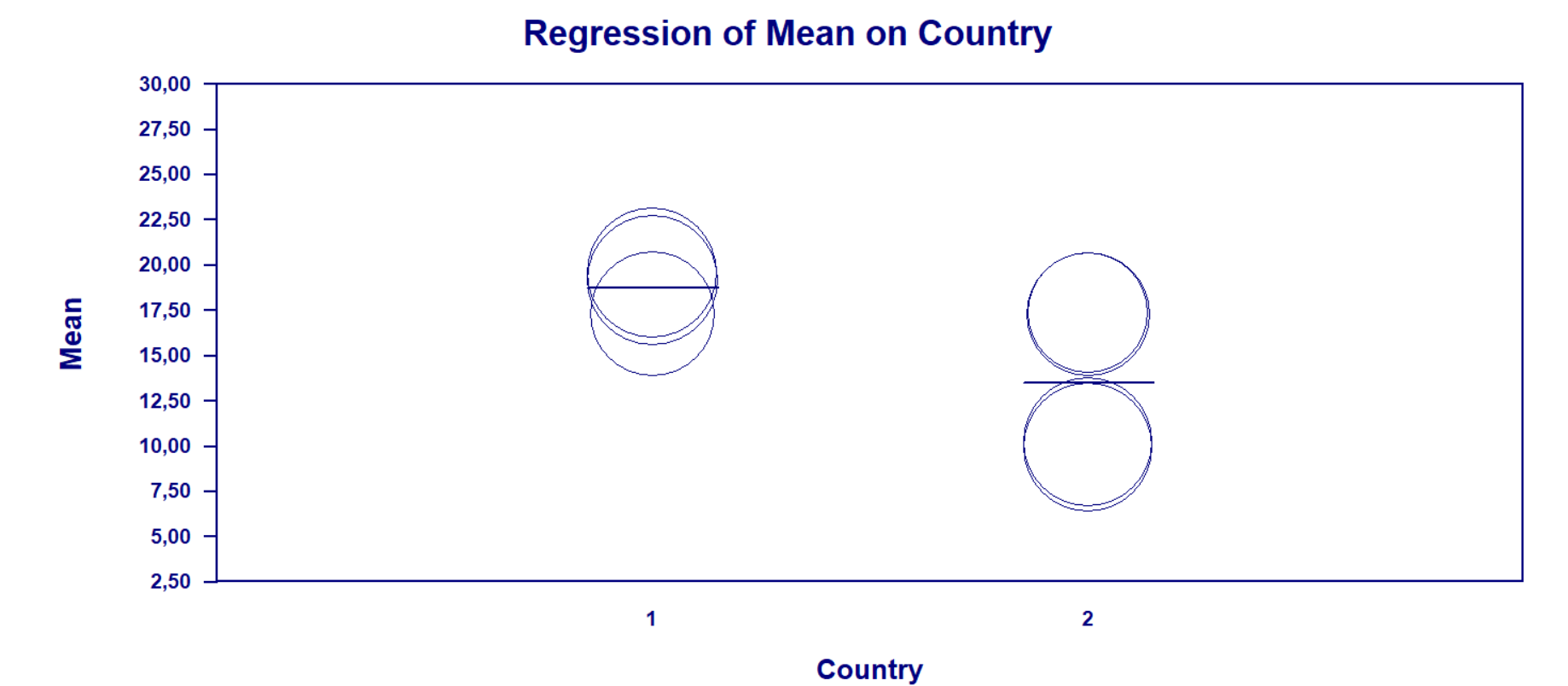
**

**S12. Scatter Plot of the moderation effect of age on Dependence
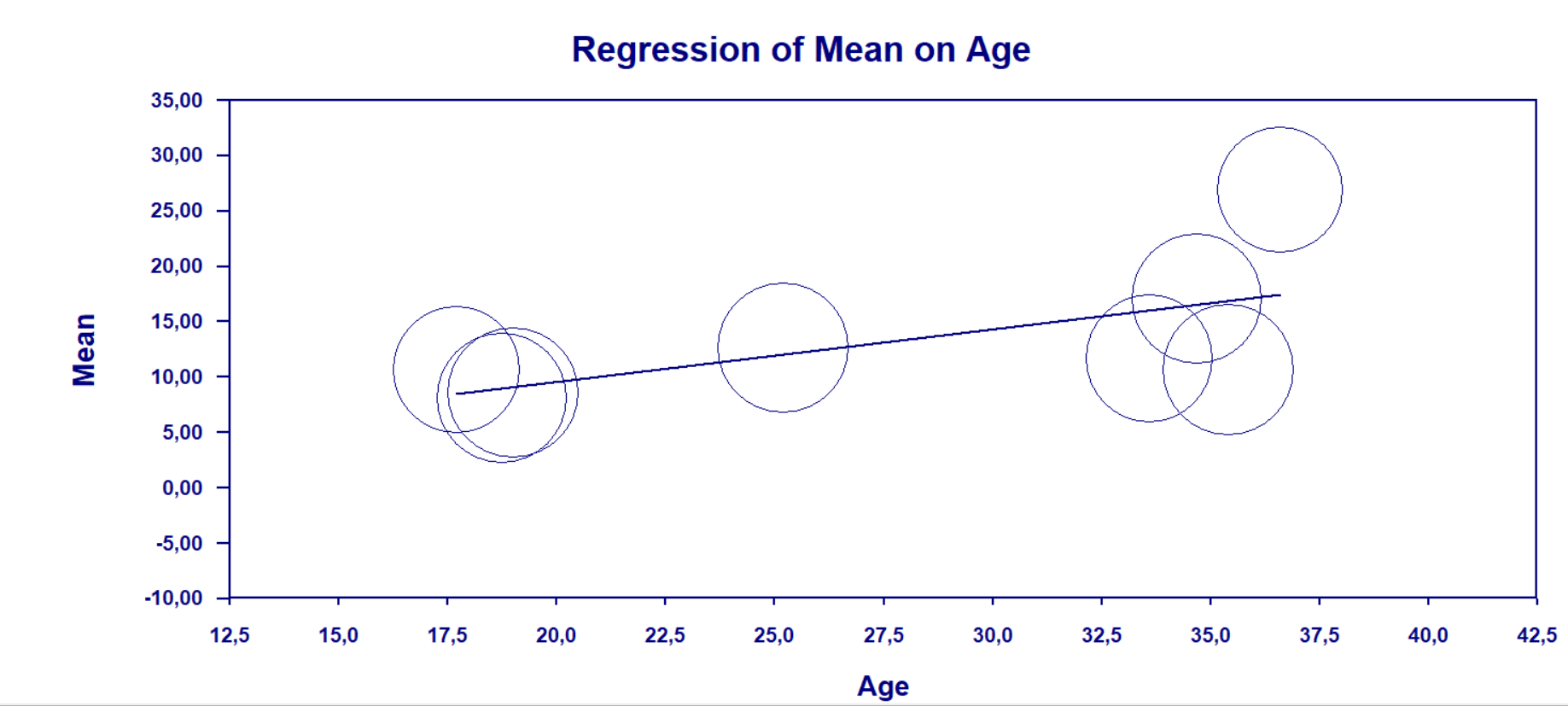
**

**S13. Scatter Plot of the moderation effect of country on Dependence**

**
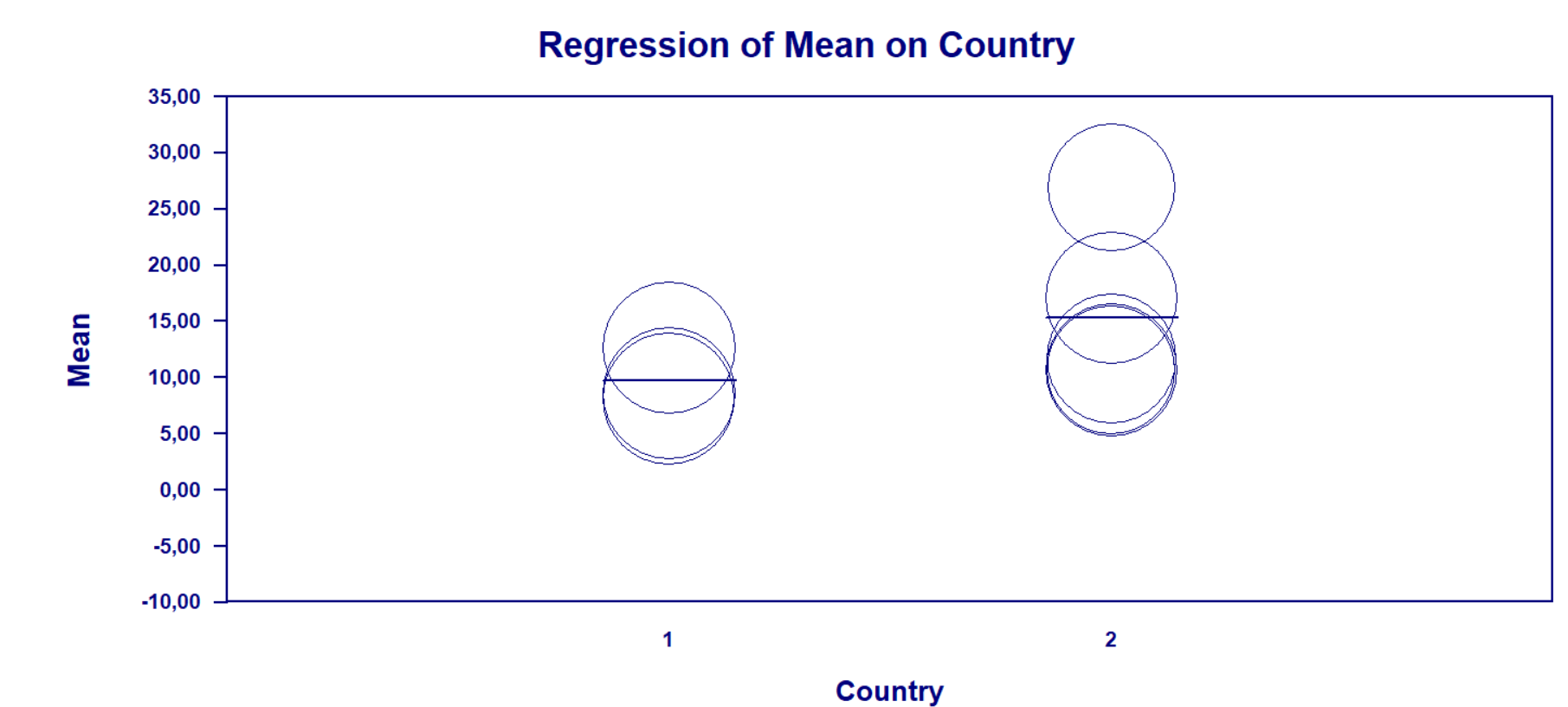
**

**S14. Scatter Plot of the moderation effect of age on Vulnerability to Harm
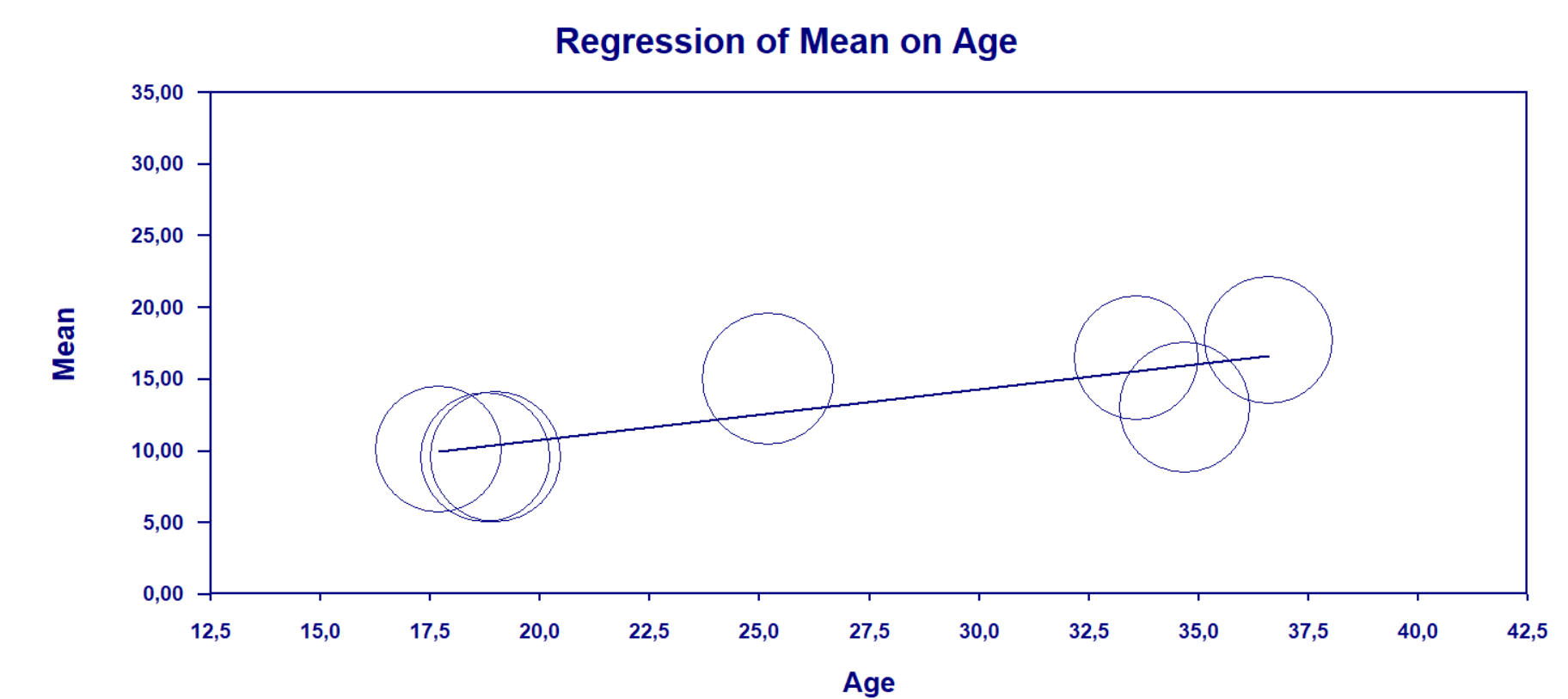
**

**S15. Scatter Plot of the moderation effect of age on Emotional Deprivation
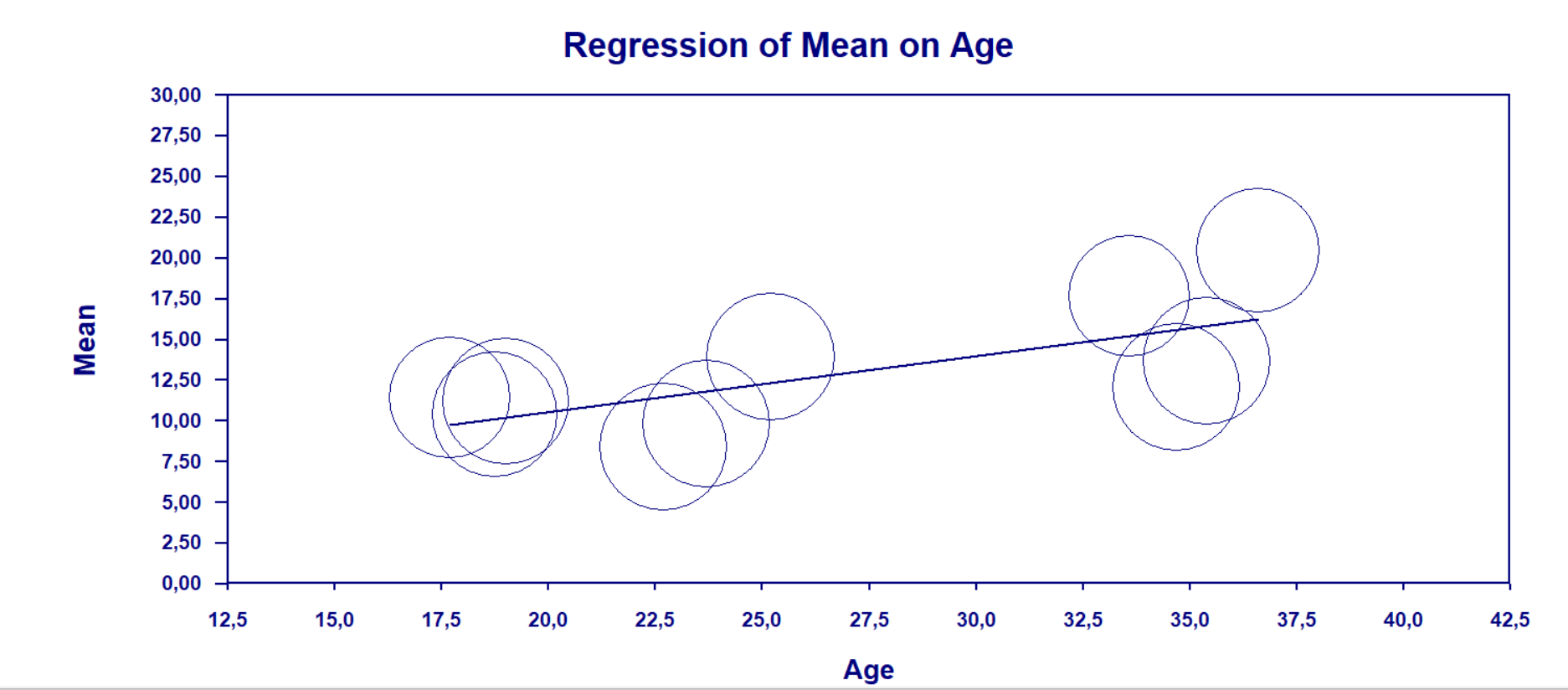
**

**S16. Scatter Plot of the moderation effect of gender on Emotional Deprivation
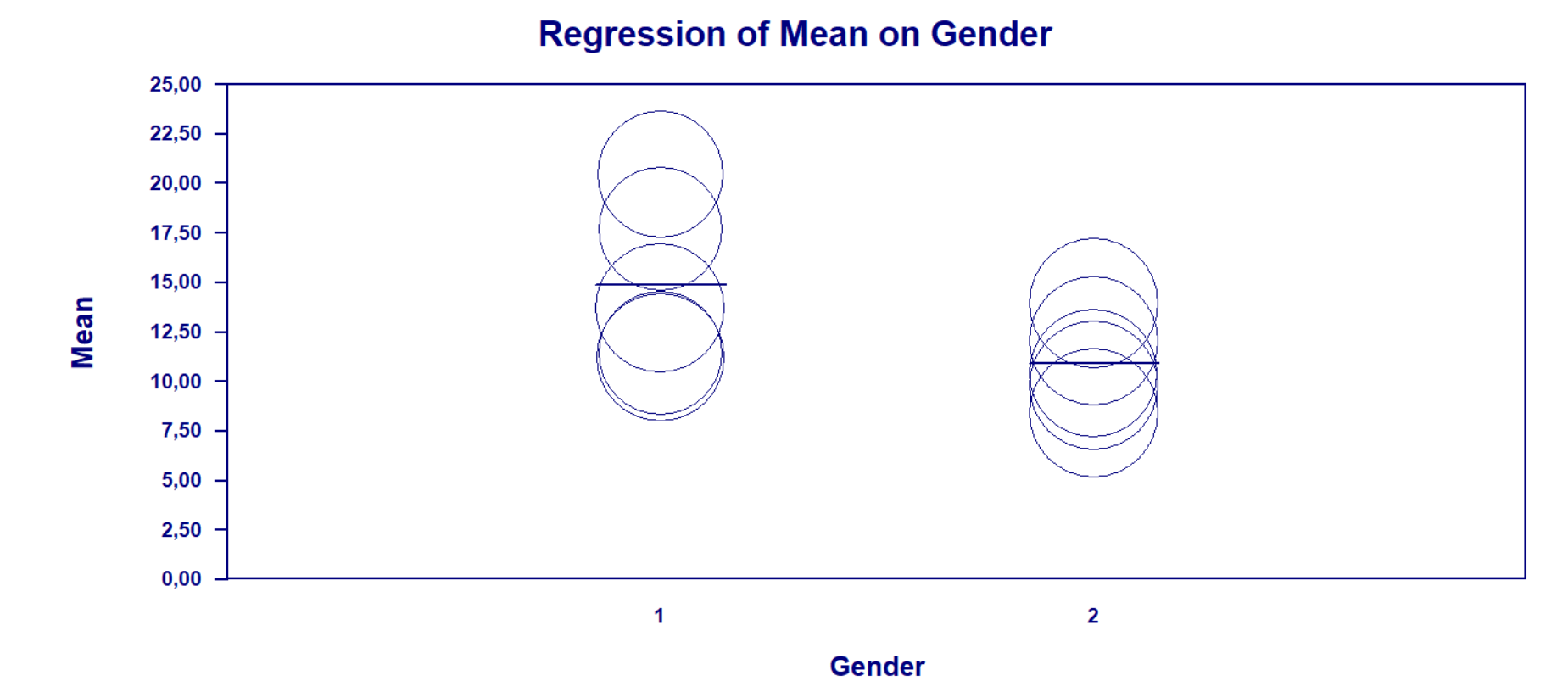
**

**S17. Scatter Plot of the moderation effect of country on Emotional Deprivation**

**
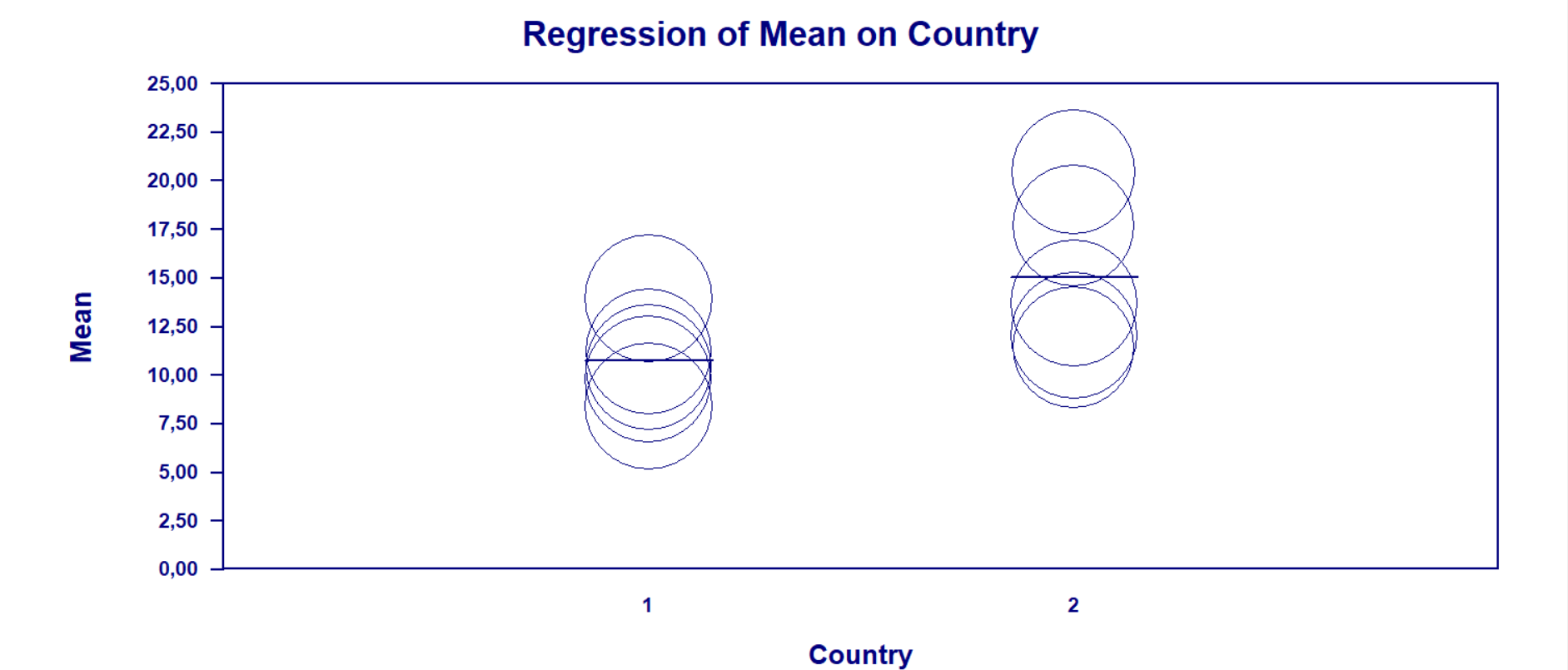
**

**S18. Forest Plot of Emotional Deprivation in psychological abuse victims
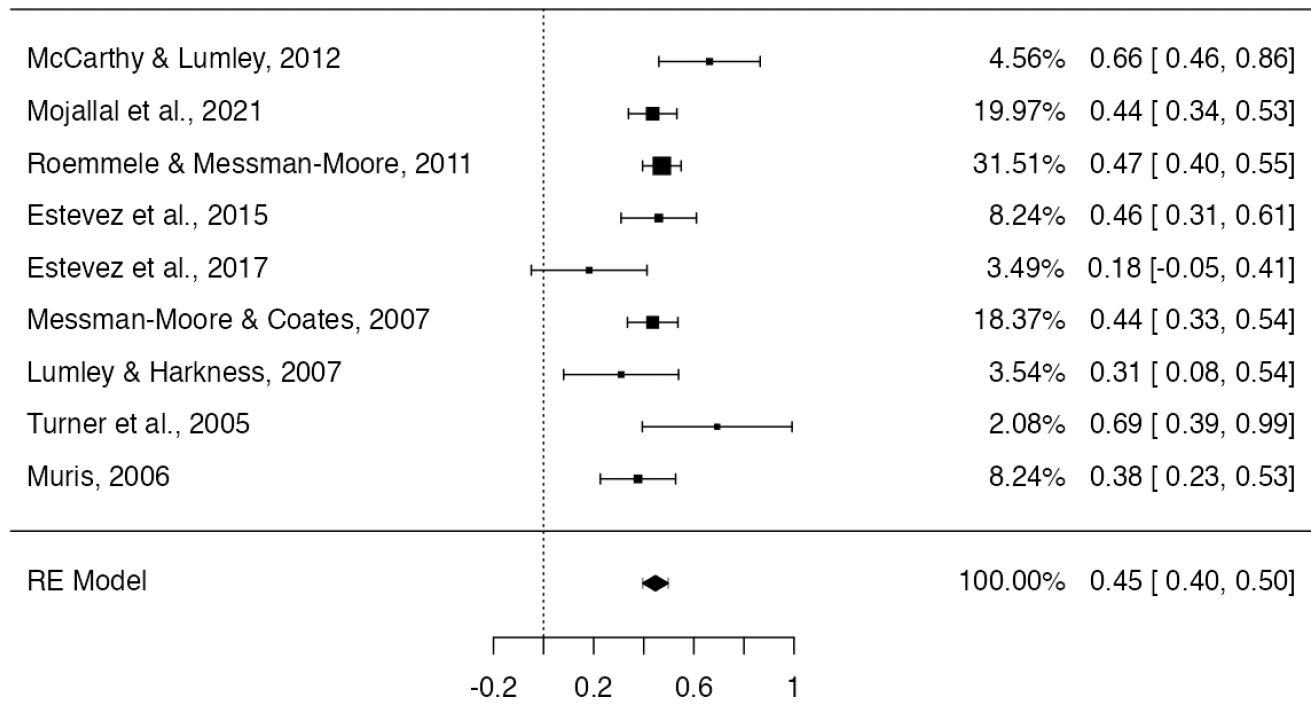
**

**S19. Funnel Plot of Emotional Deprivation in psychological abuse victims
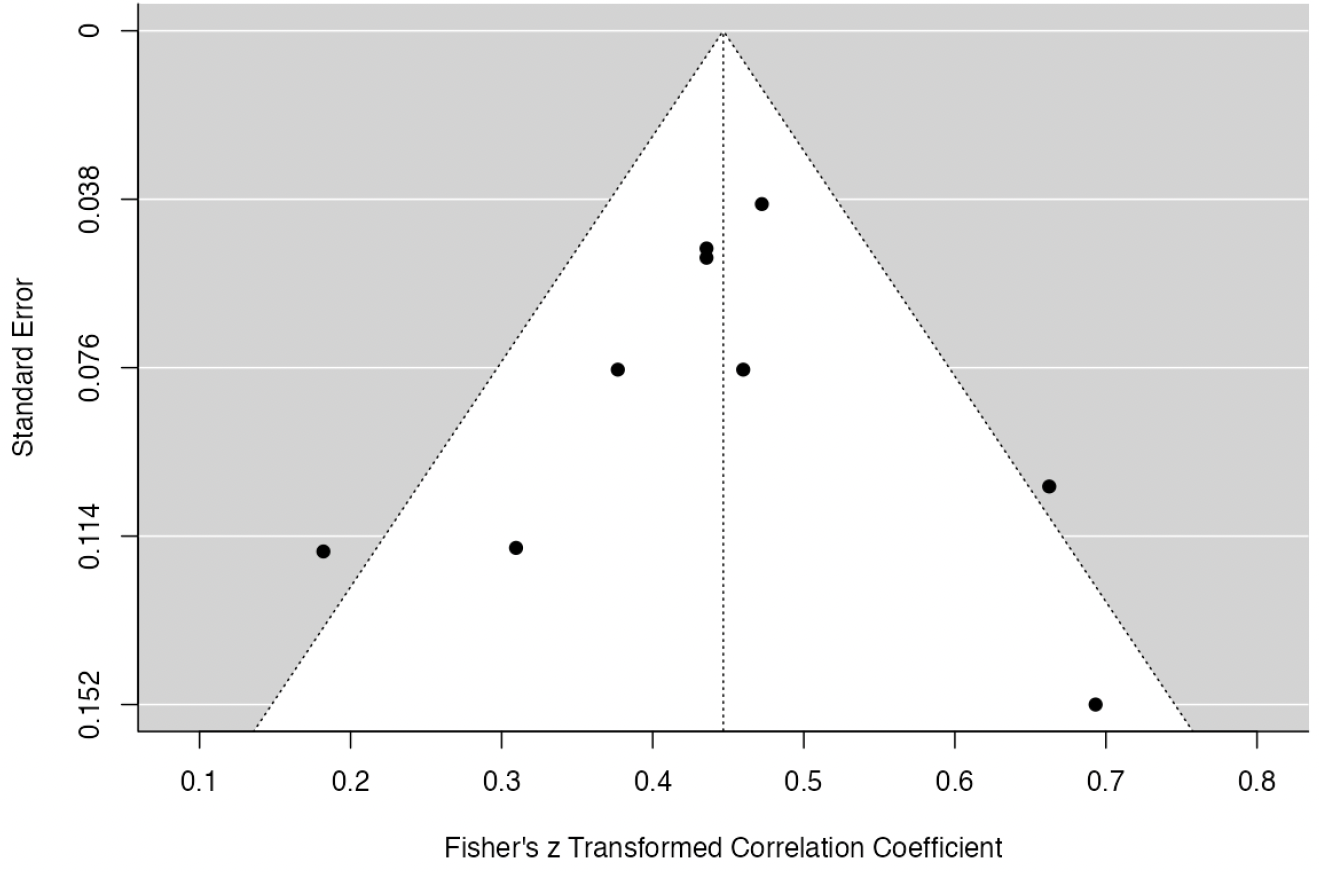
**

**S20. Forest Plot of Abuse in psychological abuse victims
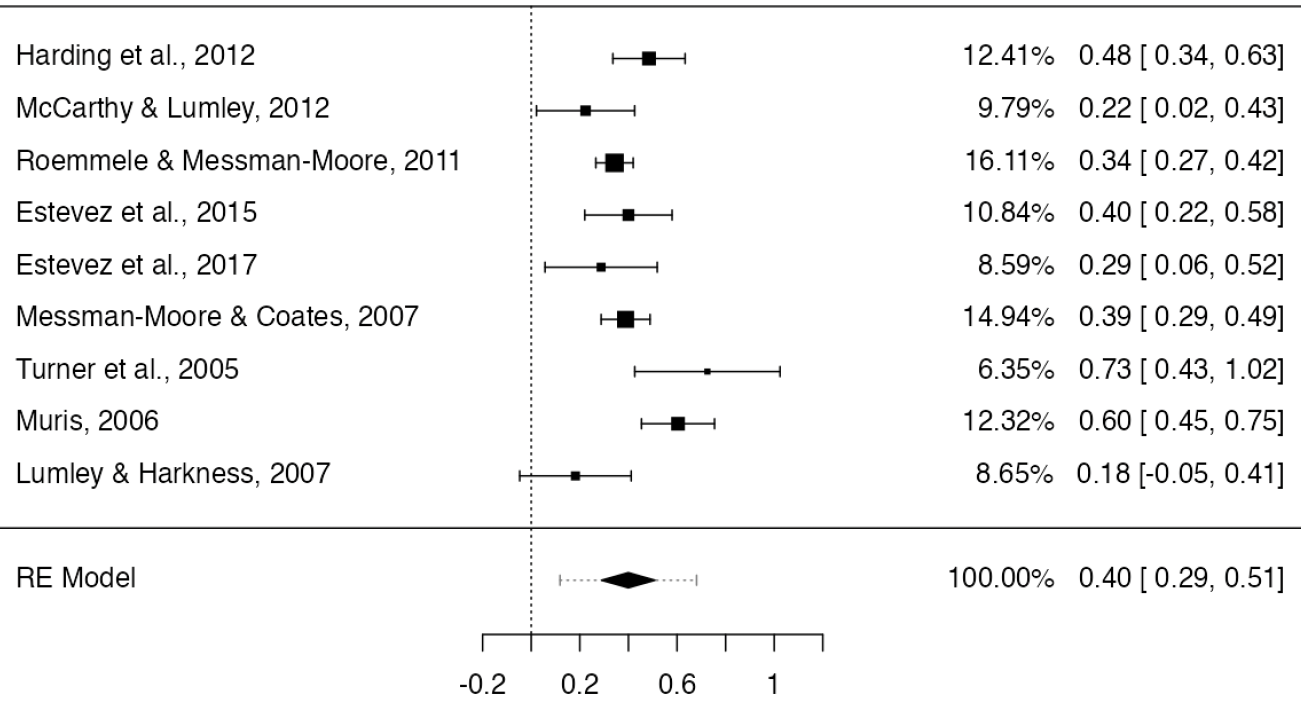
**

**S21. Funnel Plot of Abuse in psychological abuse victims
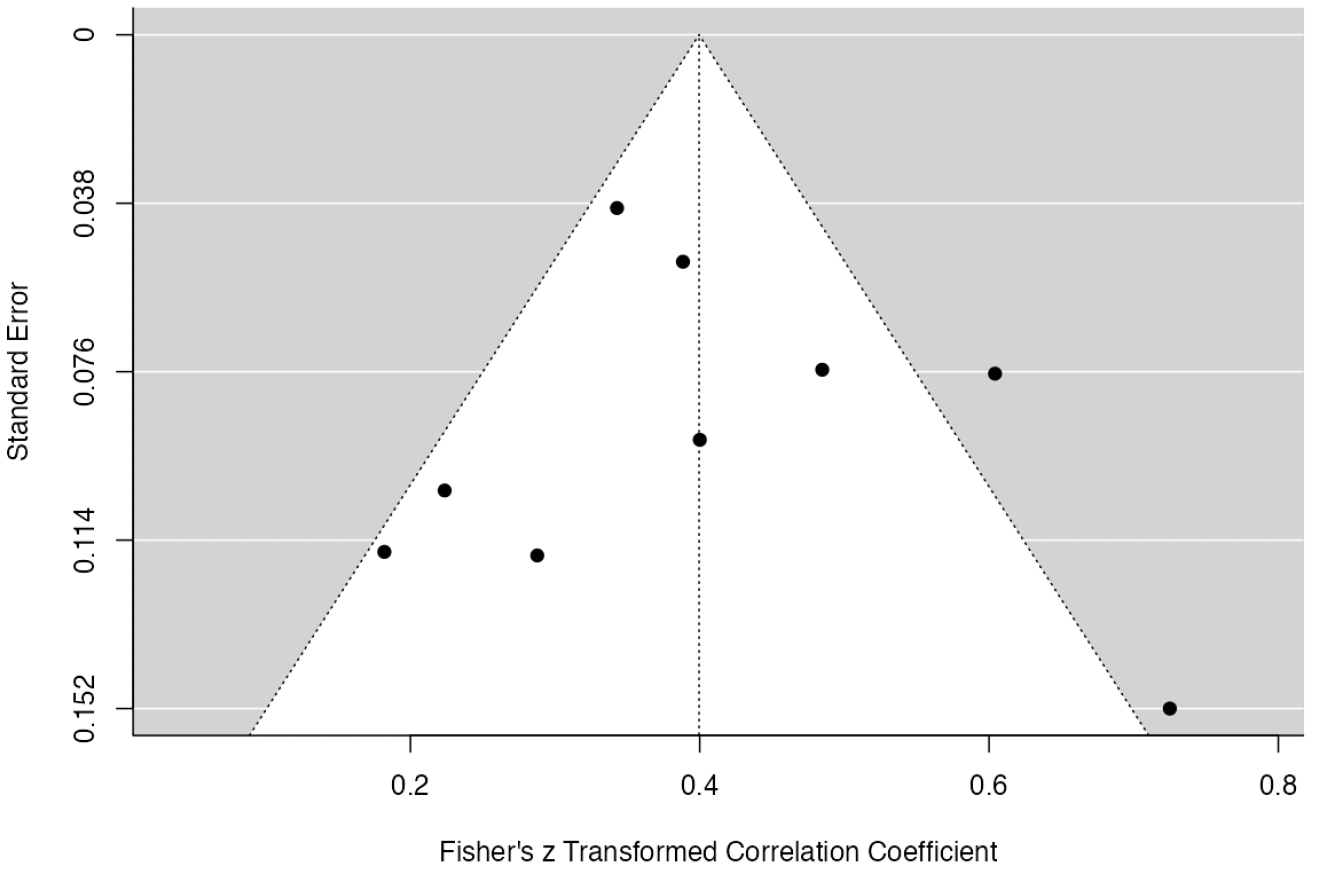
**

**S22. Forest Plot of Social Isolation in psychological abuse victims
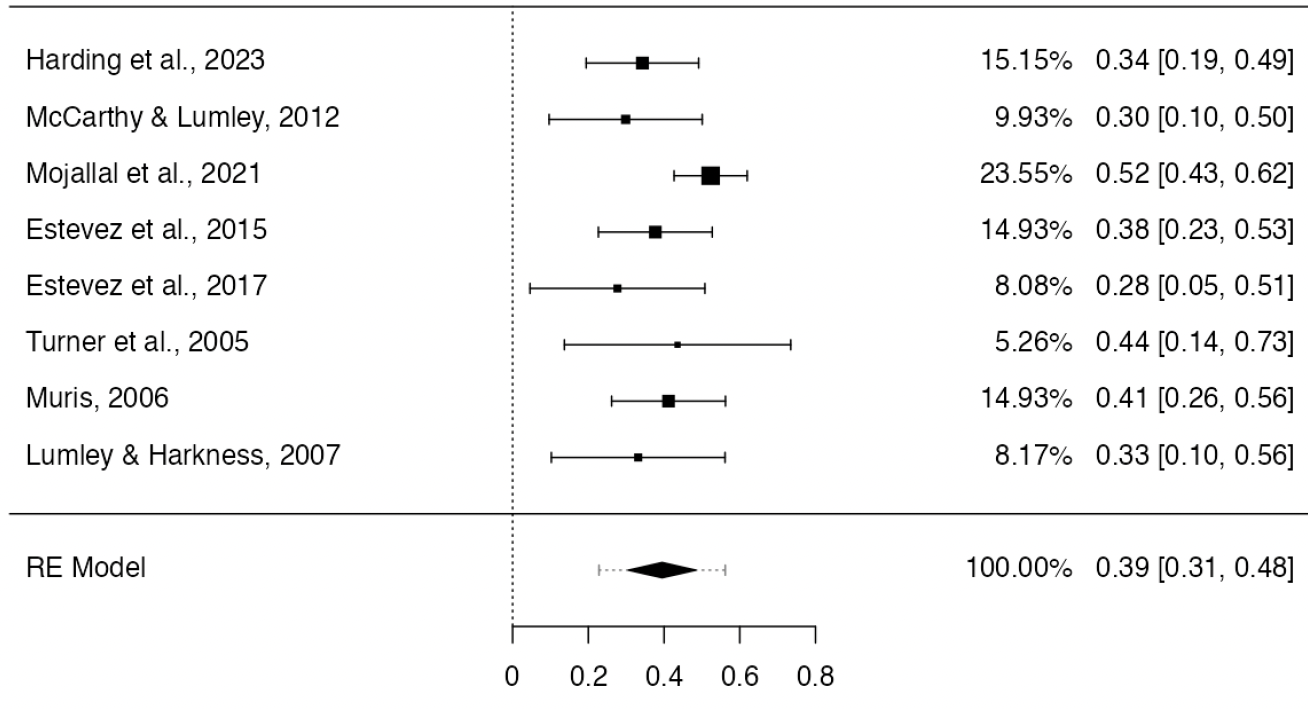
**

**S23. Funnel Plot of Social Isolation in psychological abuse victims
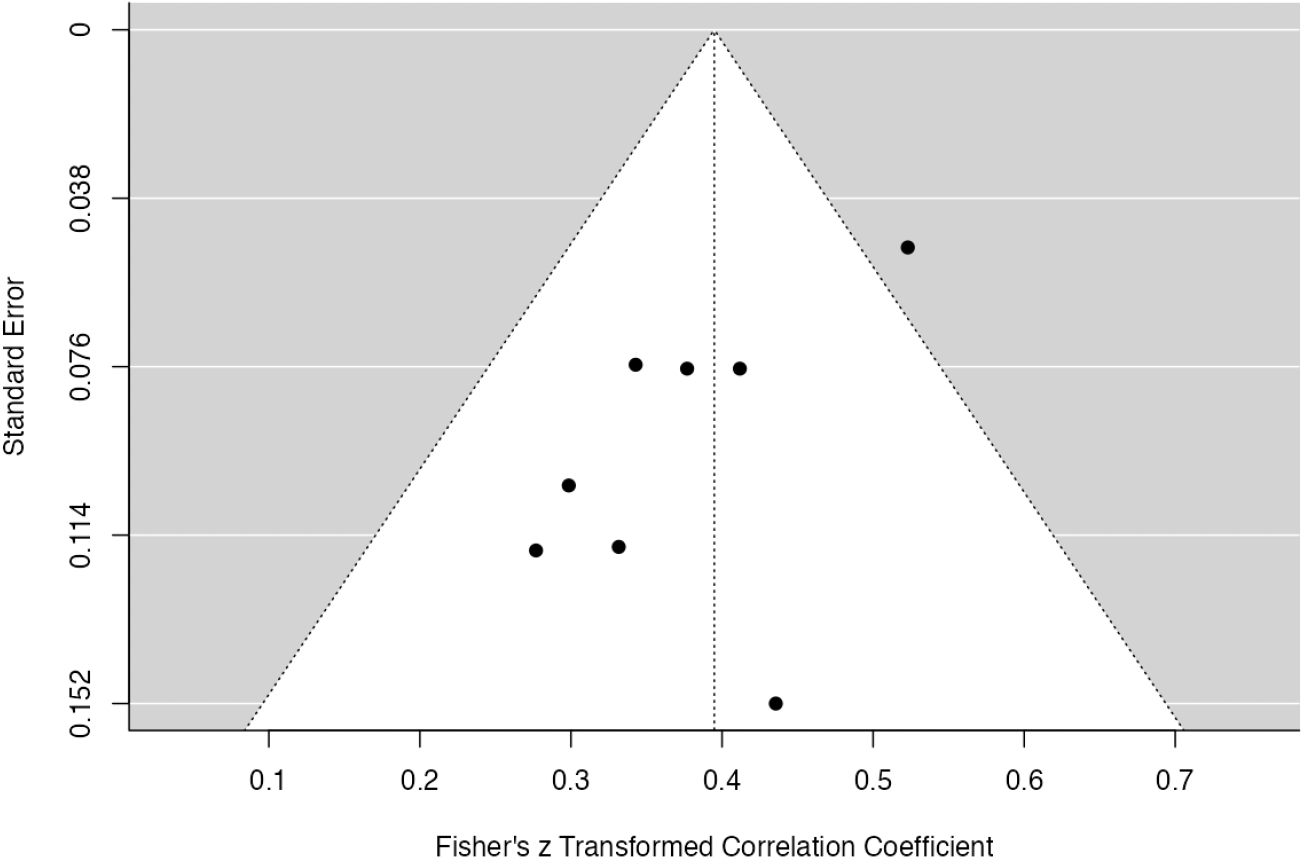
**

**S24. Forest Plot of Failure in psychological abuse victims
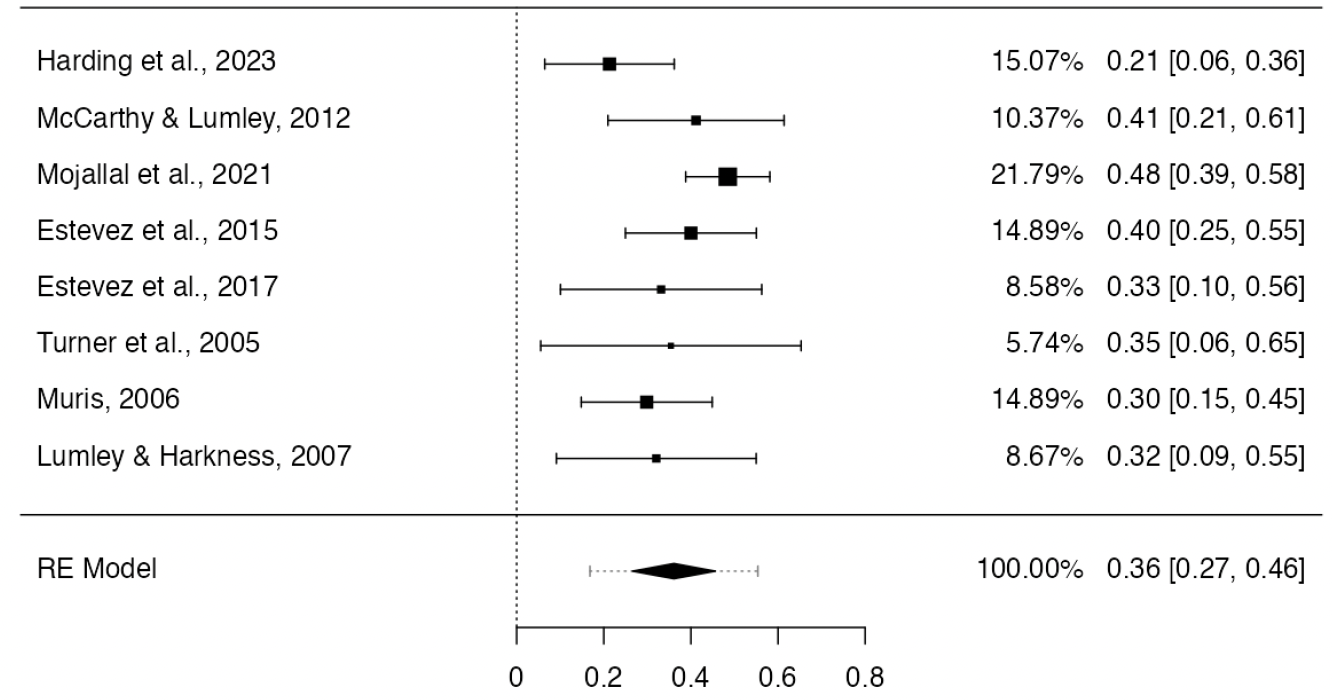
**

**S25. Funnel Plot of Failure in psychological abuse victims
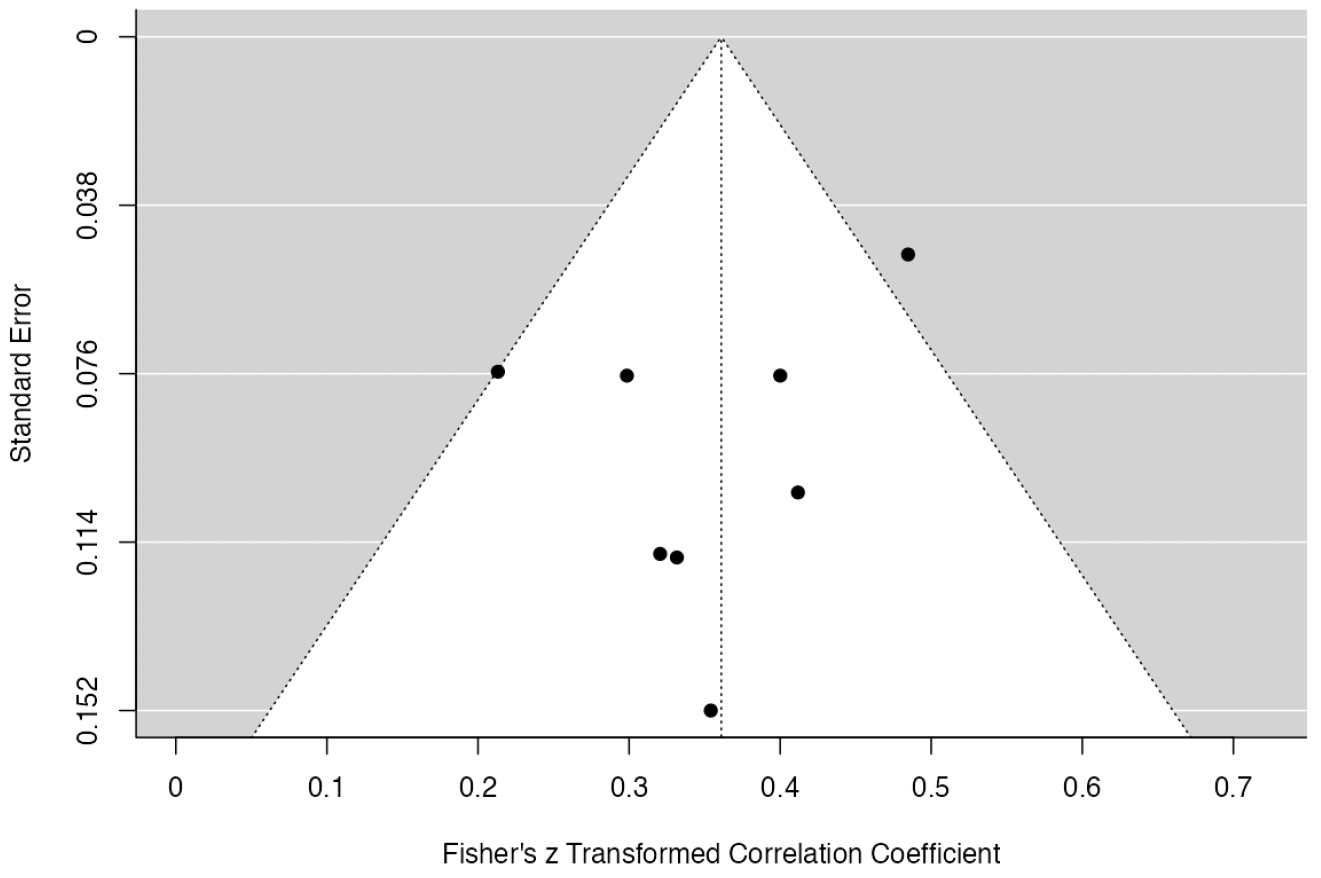
**

**S26. Forest Plot of Abandonment in psychological abuse victims
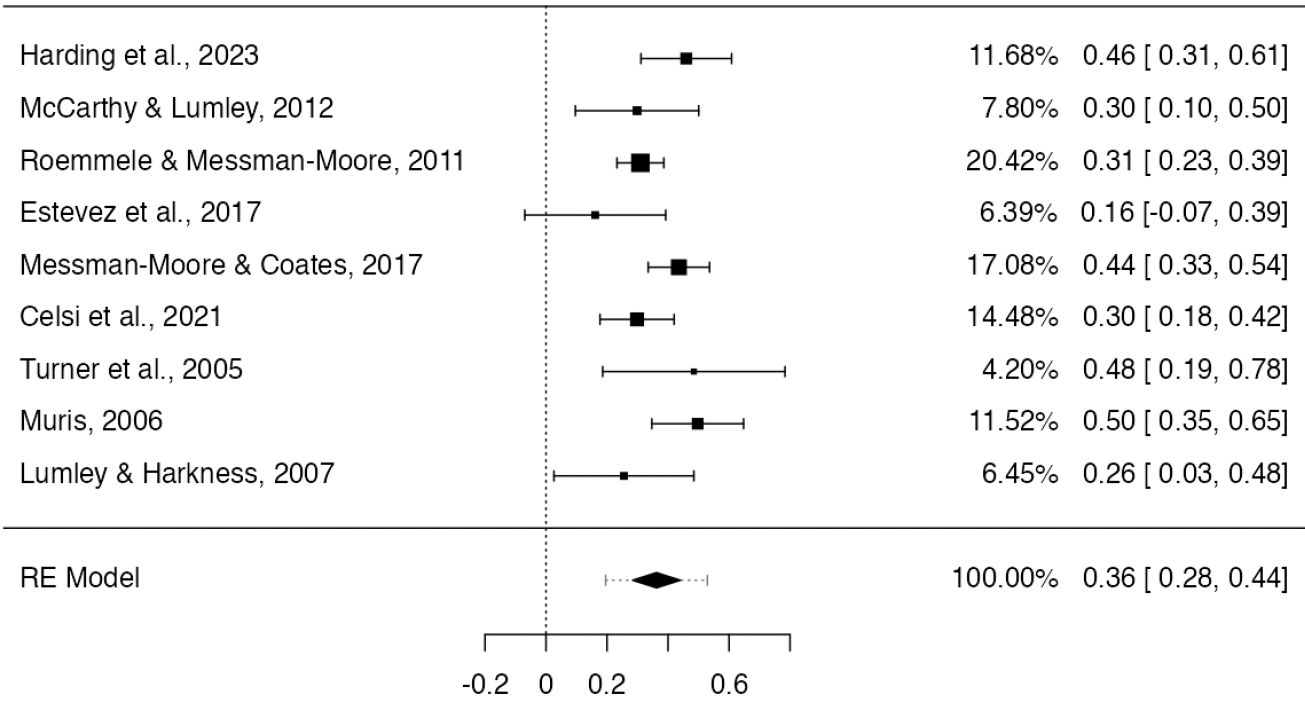
**

**S27. Funnel Plot of Abandonment in psychological abuse victims
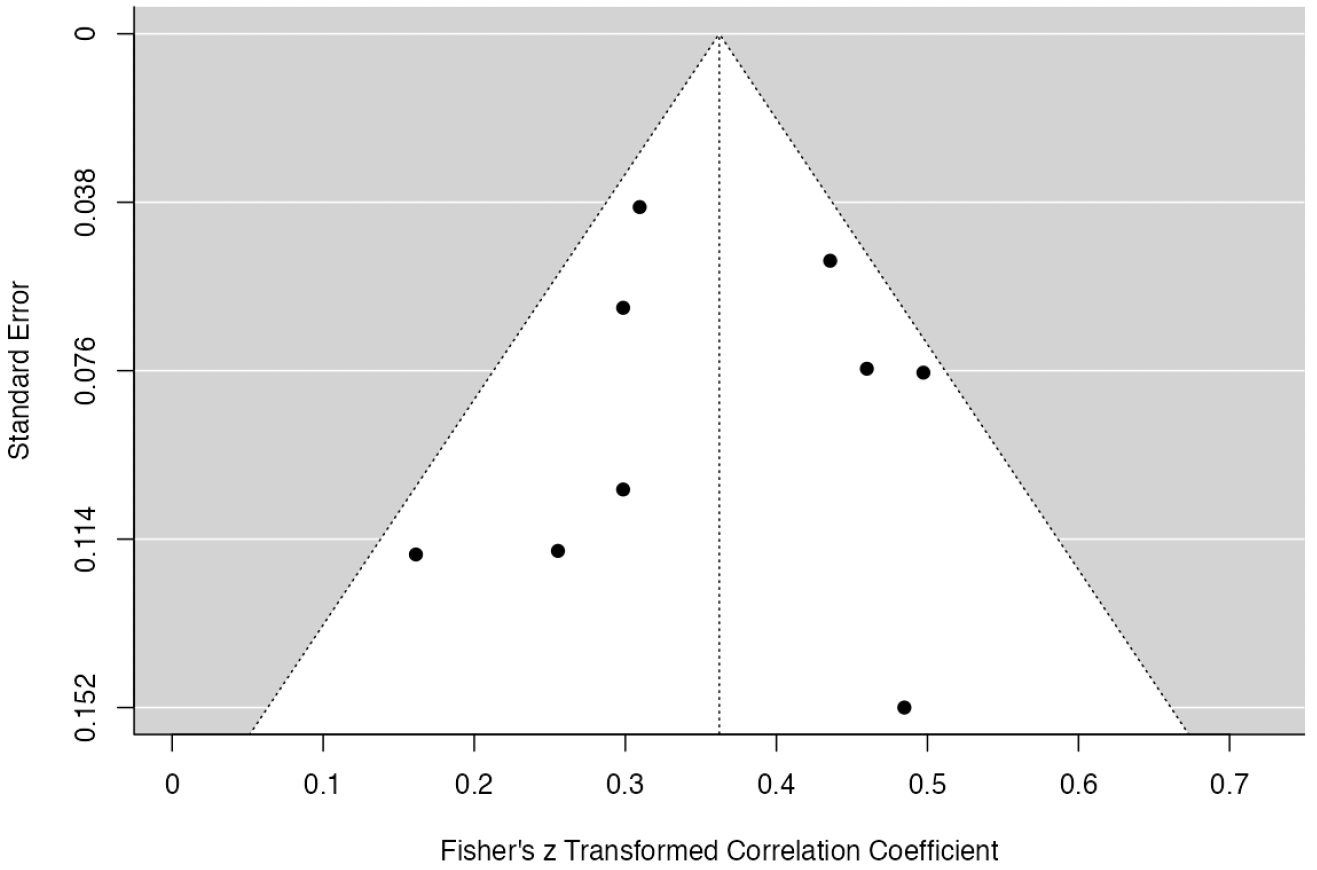
**

**S28. Forest Plot of Emotional Inhibition in psychological abuse victims
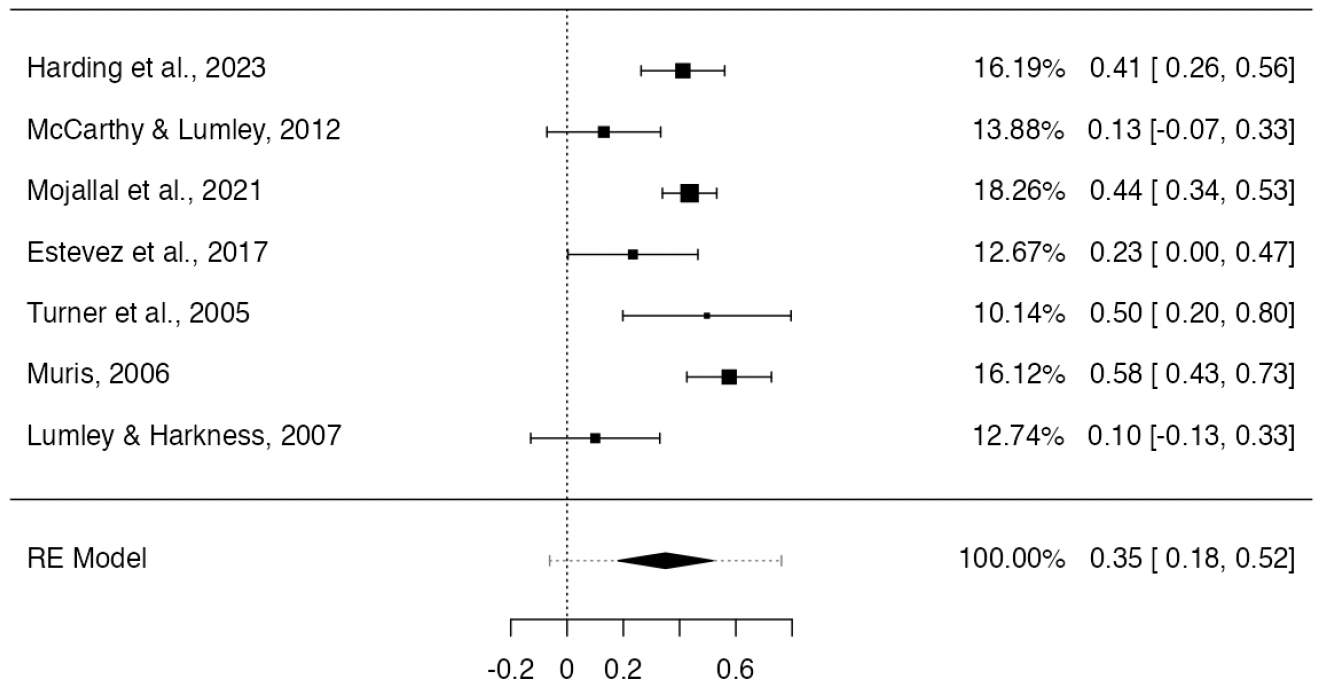
**

**S29. Funnel Plot of Emotional Inhibition in psychological abuse victims
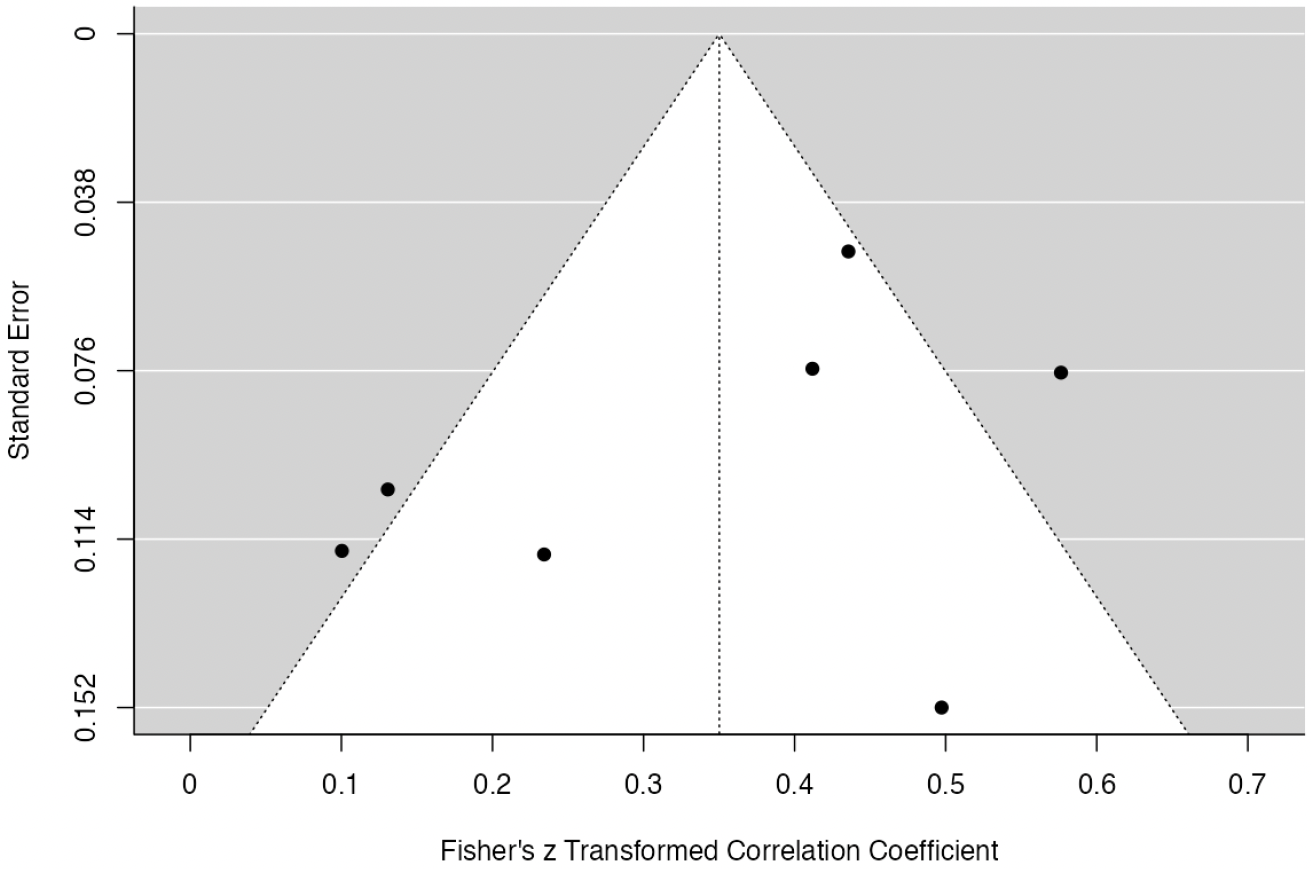
**

**S30. Forest Plot of Vulnerability to Harm in psychological abuse victims
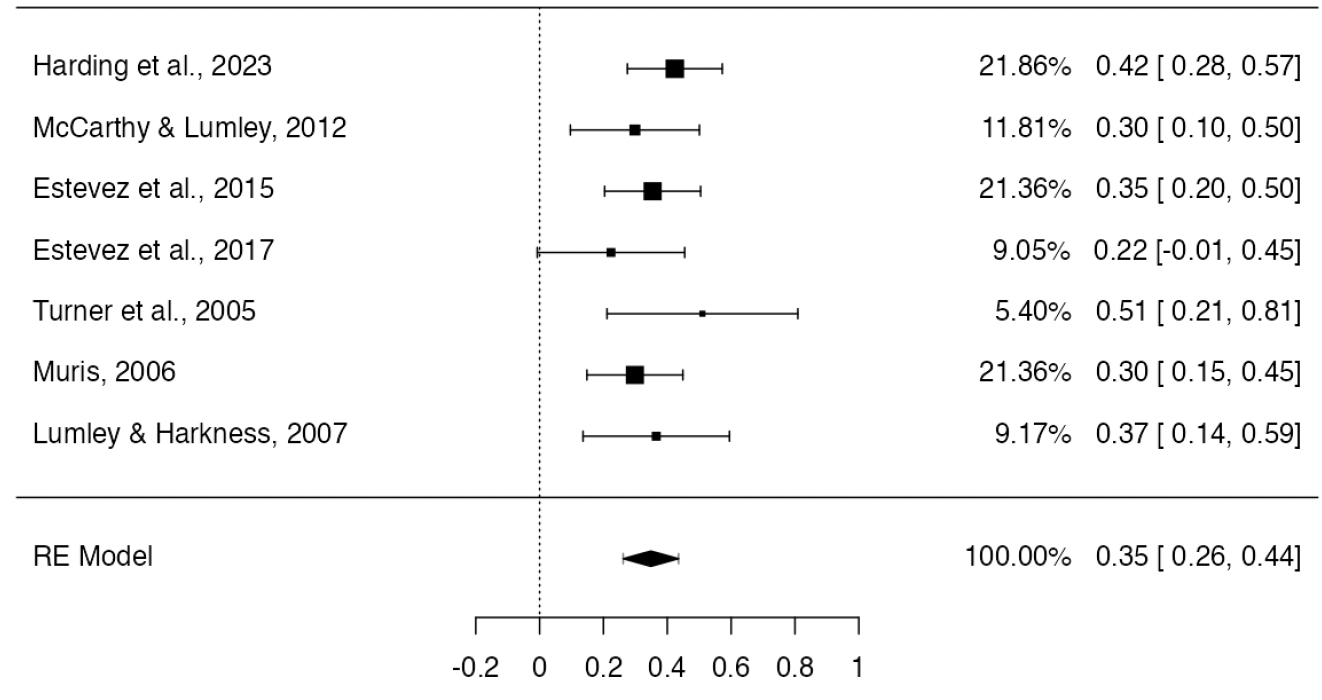
**

**S31. Funnel Plot of Vulnerability to Harm in psychological abuse victims
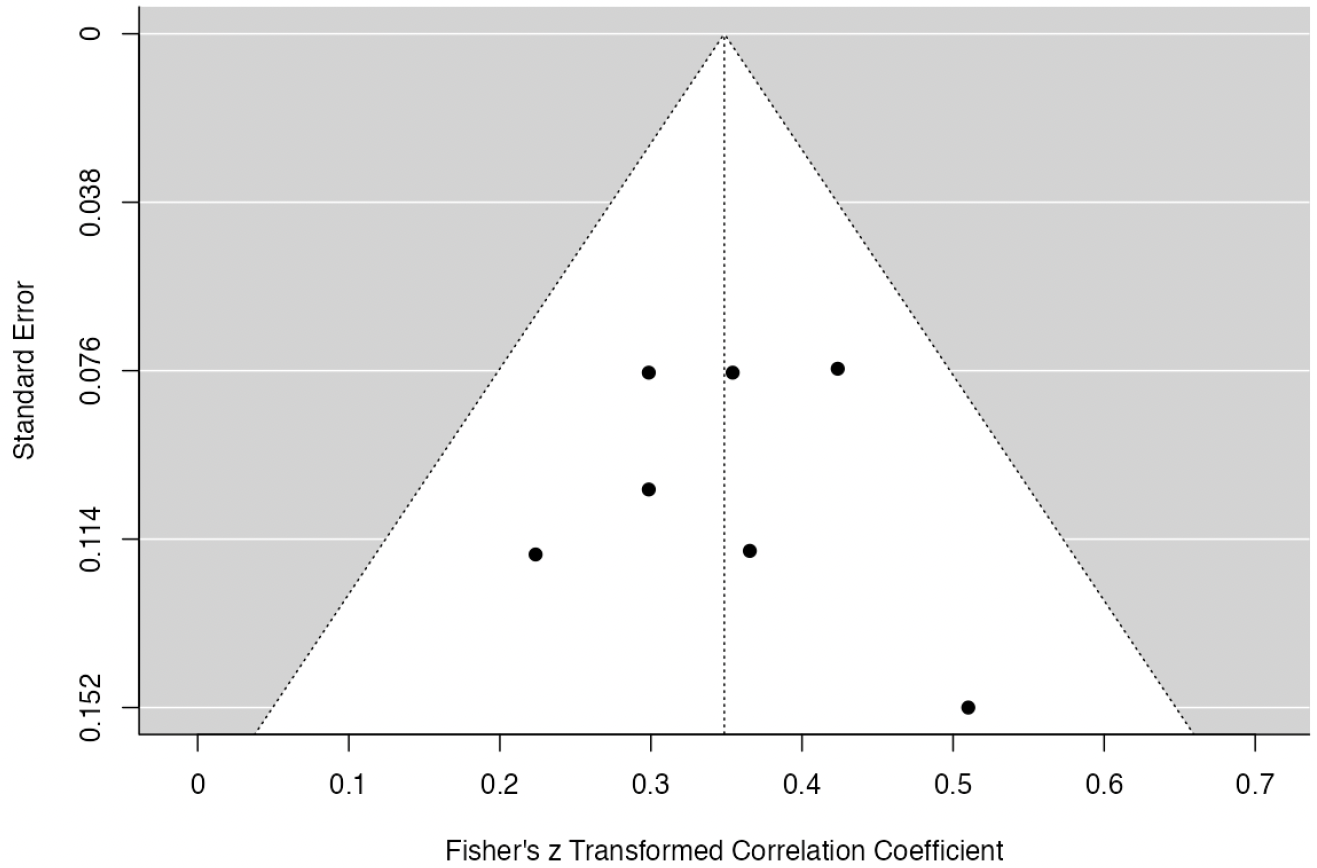
**

**S32. Forest Plot of Defectiveness in psychological abuse victims
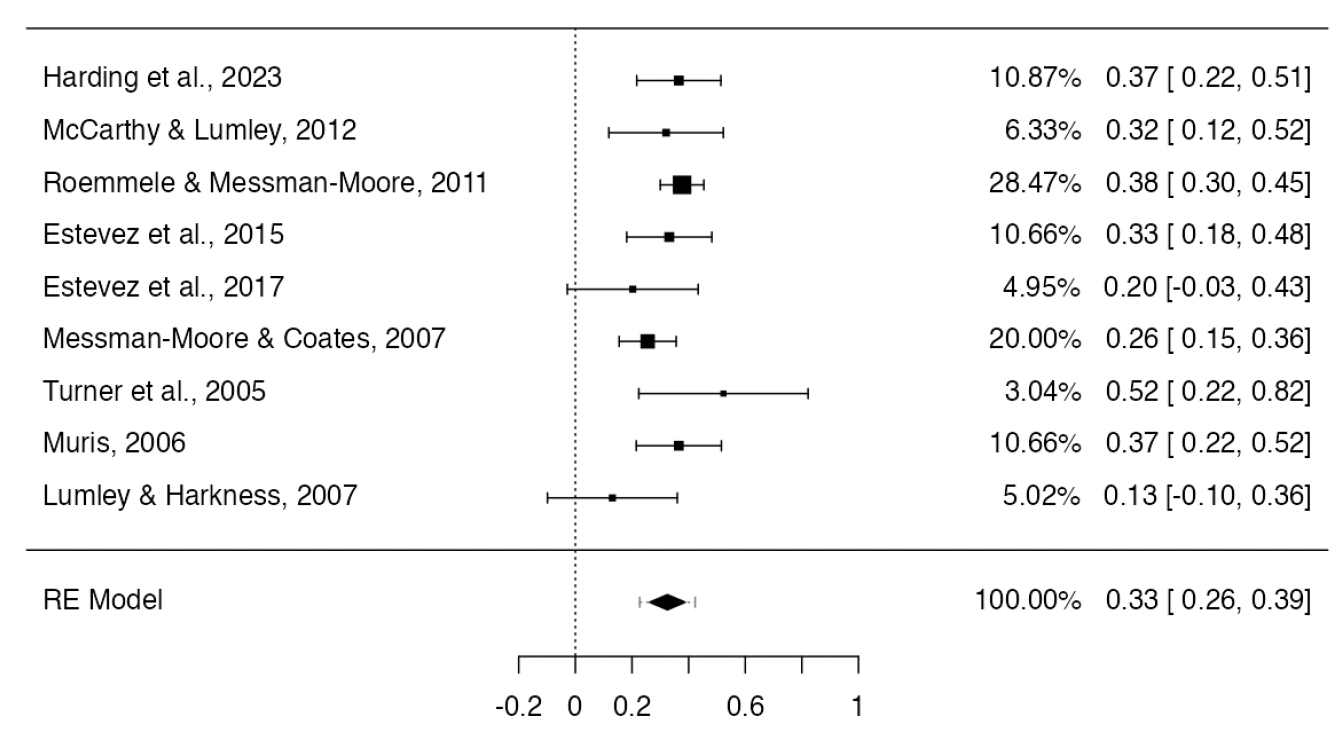
**

**S33. Funnel Plot of Defectiveness in psychological abuse victims
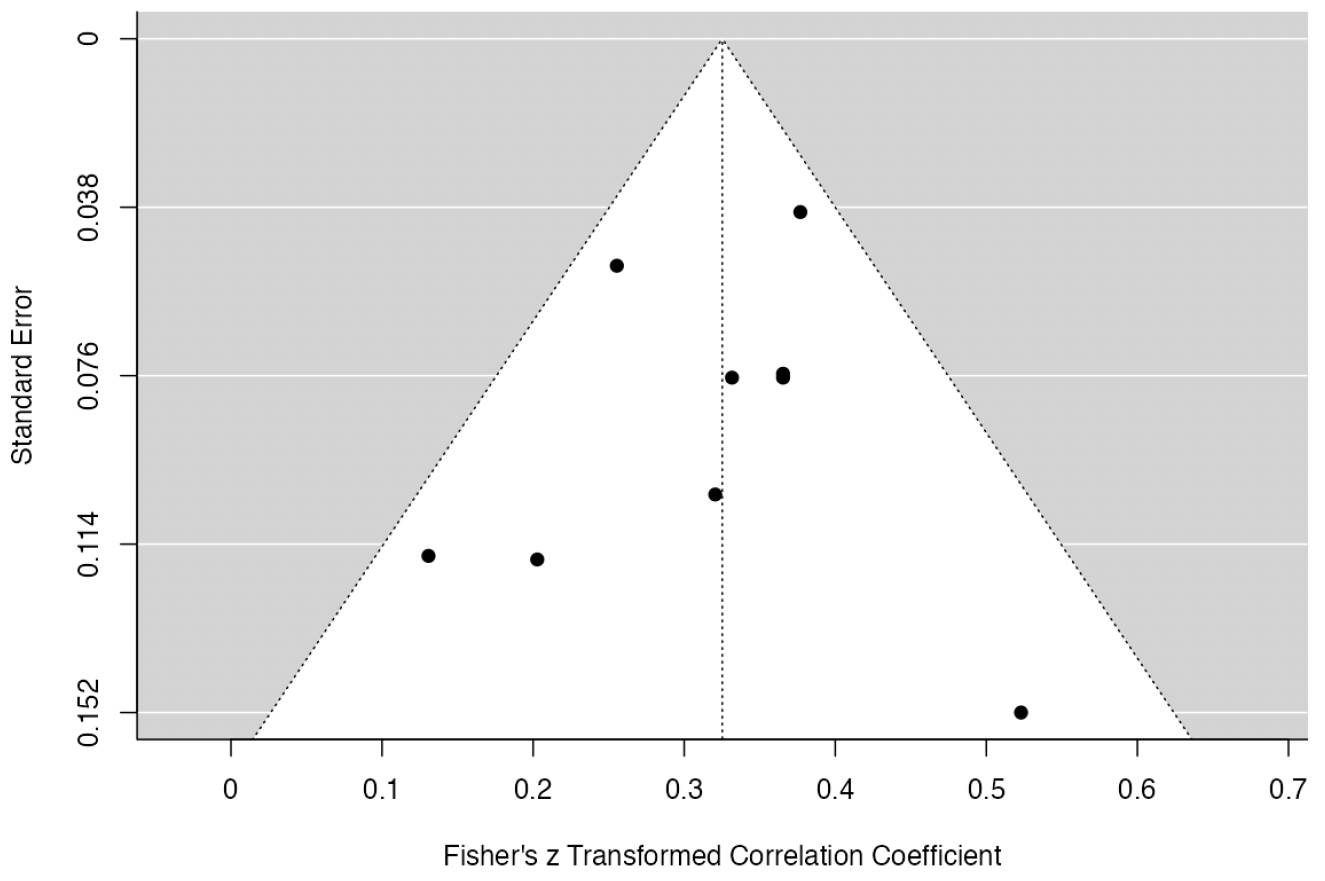
**

**S34. Forest Plot of Dependence in psychological abuse victims
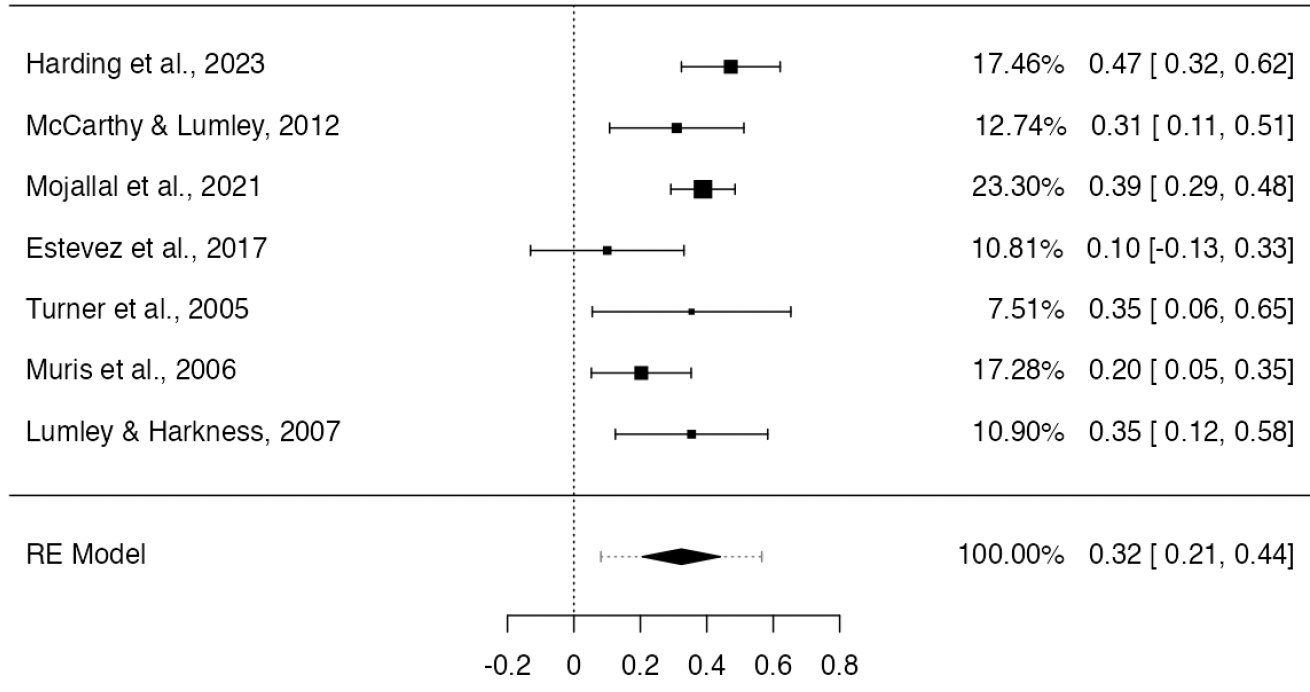
**

**S35. Funnel Plot of Dependence in psychological abuse victims
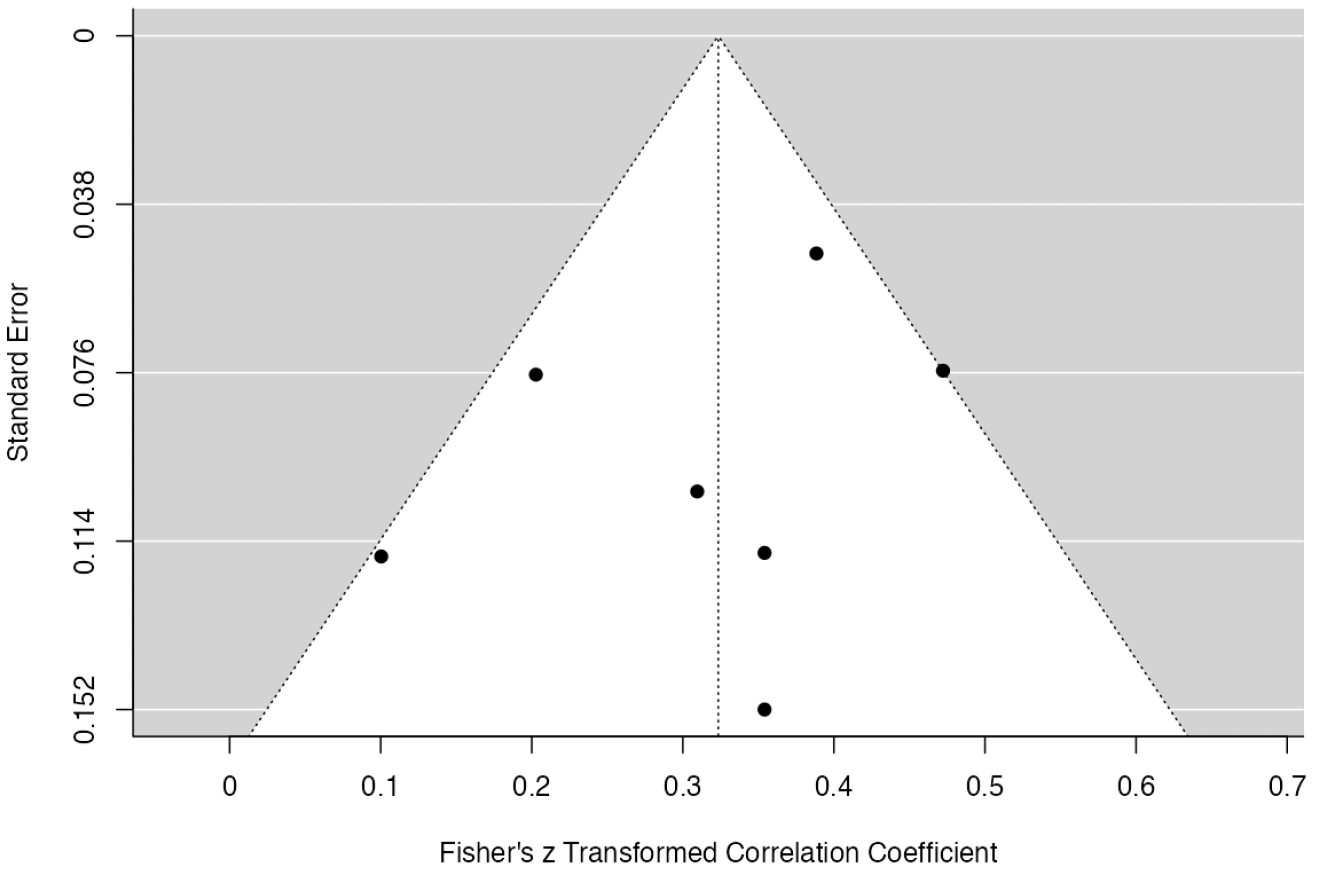
**

**S36. Forest Plot of Subjugation in psychological abuse victims
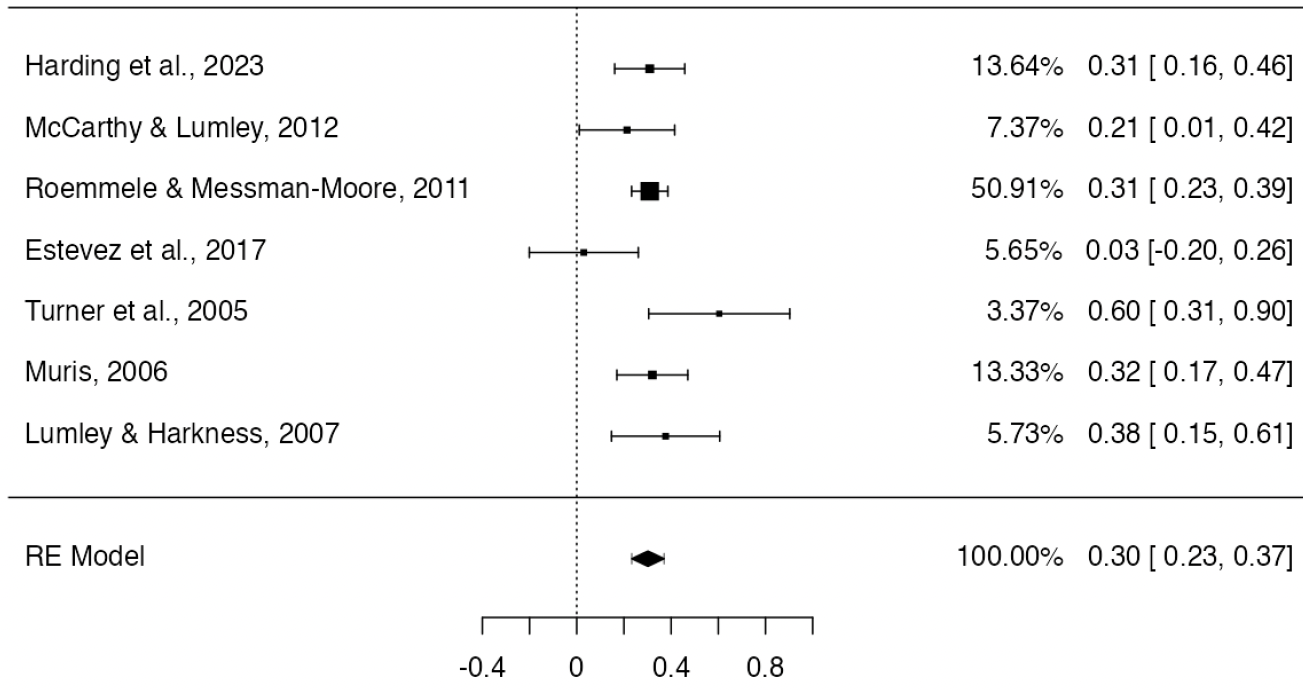
**

**S37. Funnel Plot of Subjugation in psychological abuse victims
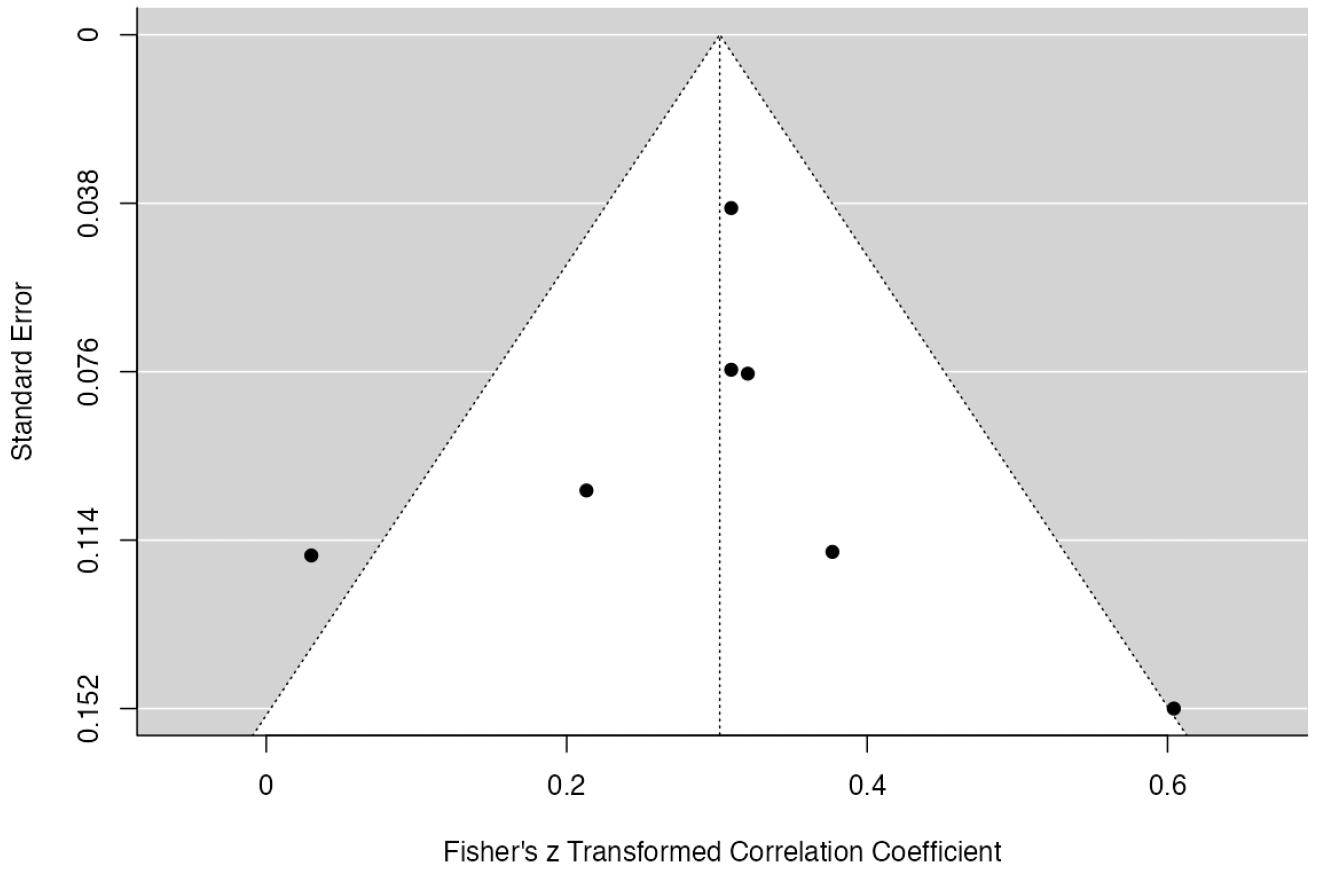
**

**S38. Forest Plot of Emotional Deprivation in physical abuse victims
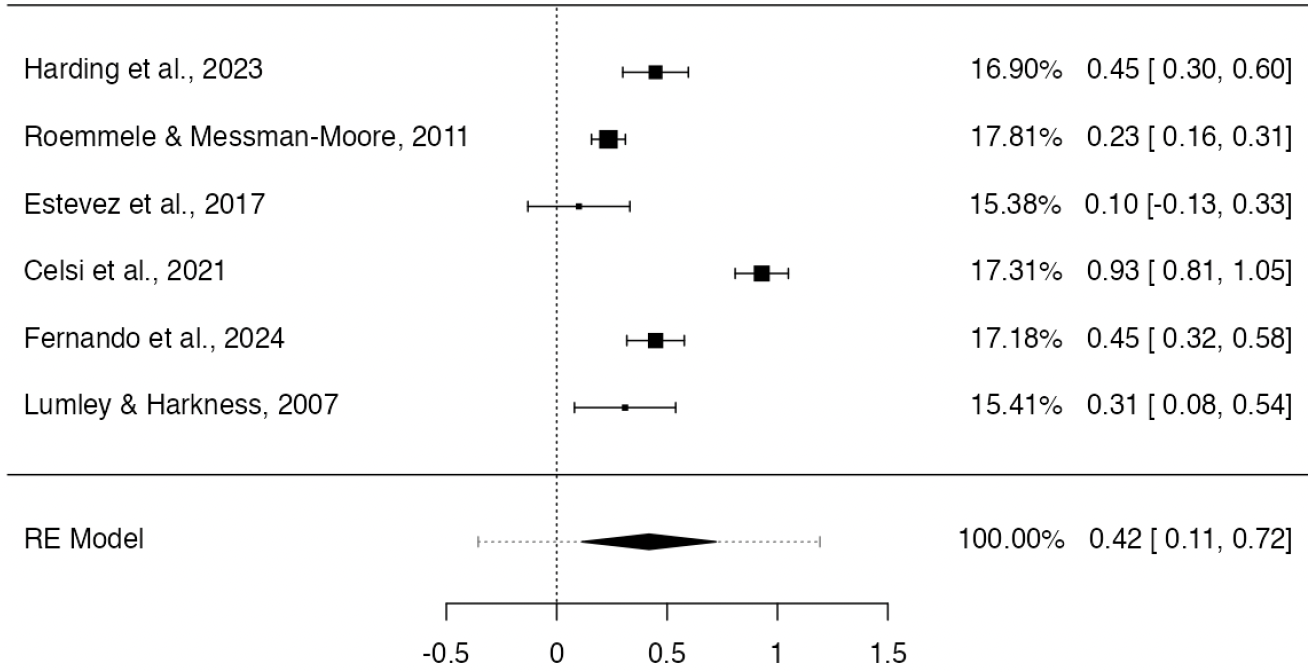
**

**S39. Funnel Plot of Emotional Deprivation in physical abuse victims
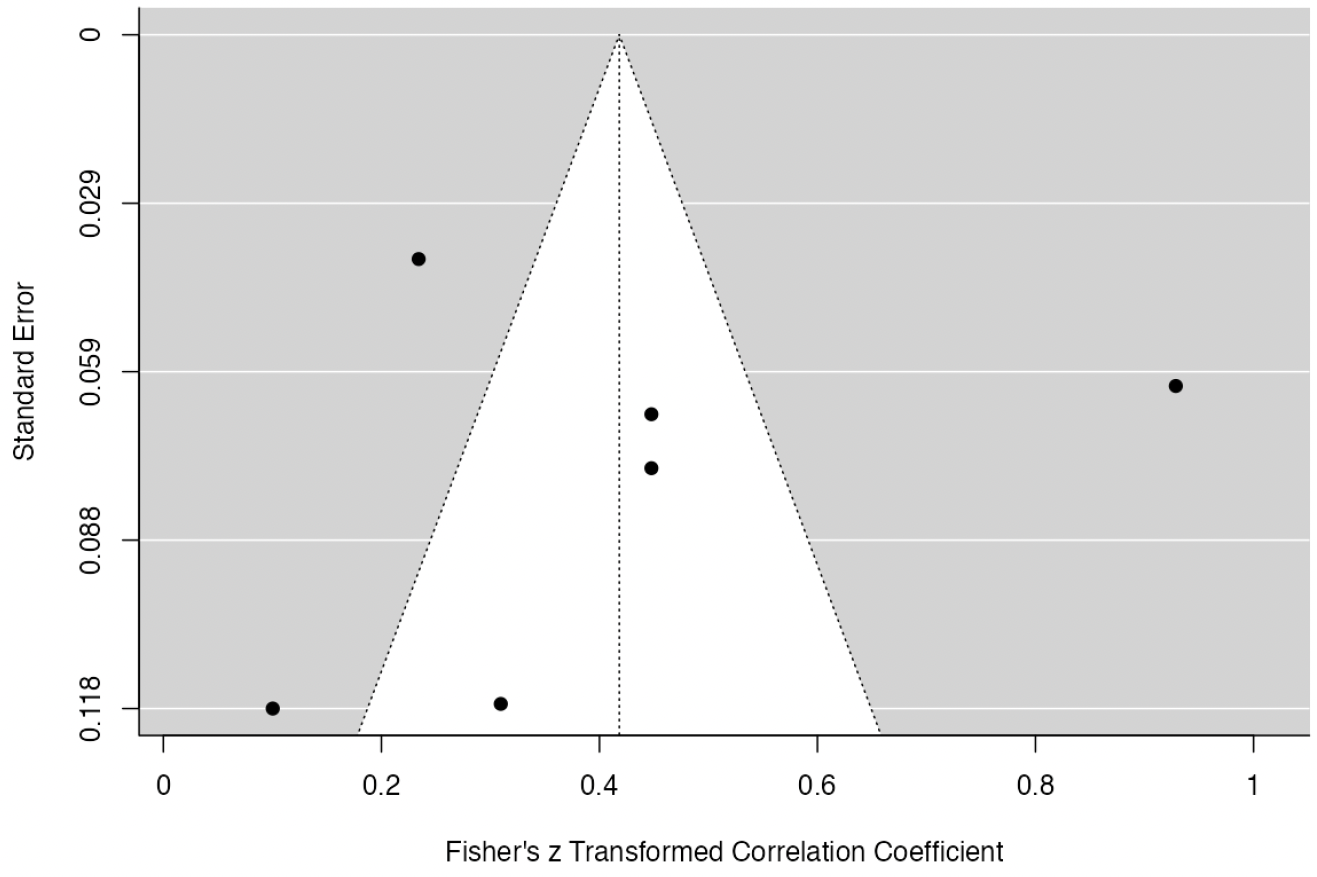
**

**S40. Forest Plot of Social Isolation in physical abuse victims
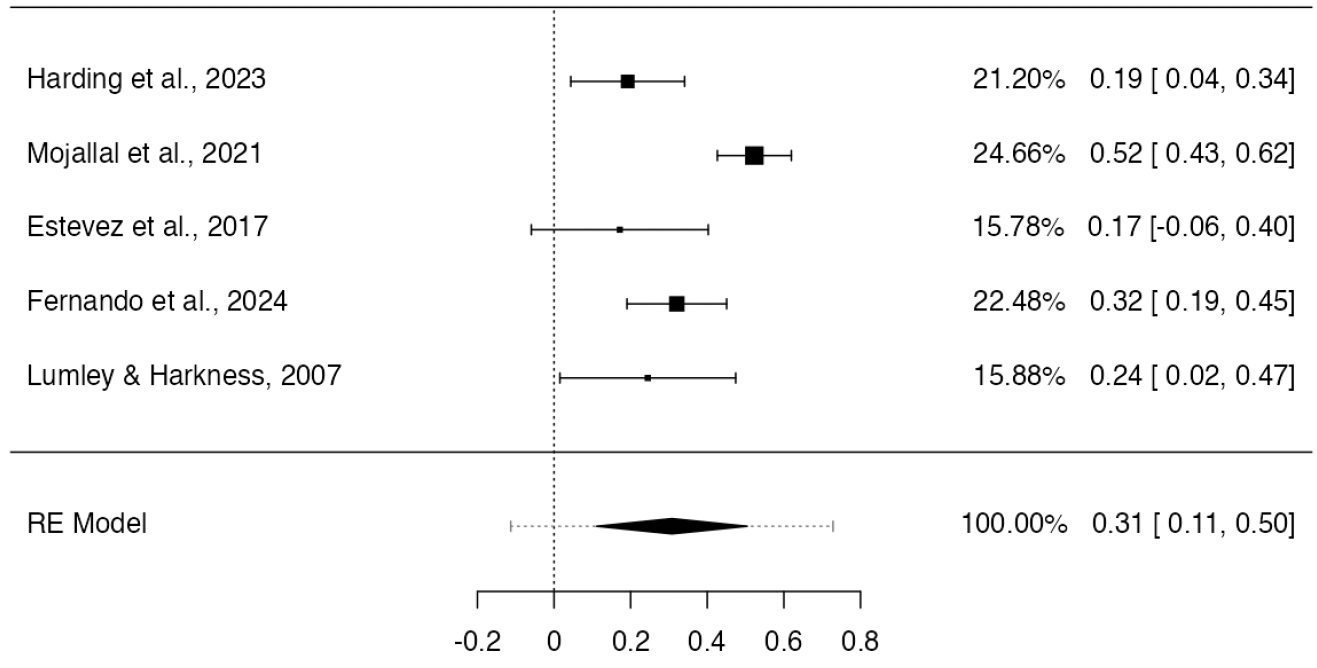
**

**S41. Funnel Plot of Social Isolation in physical abuse victims
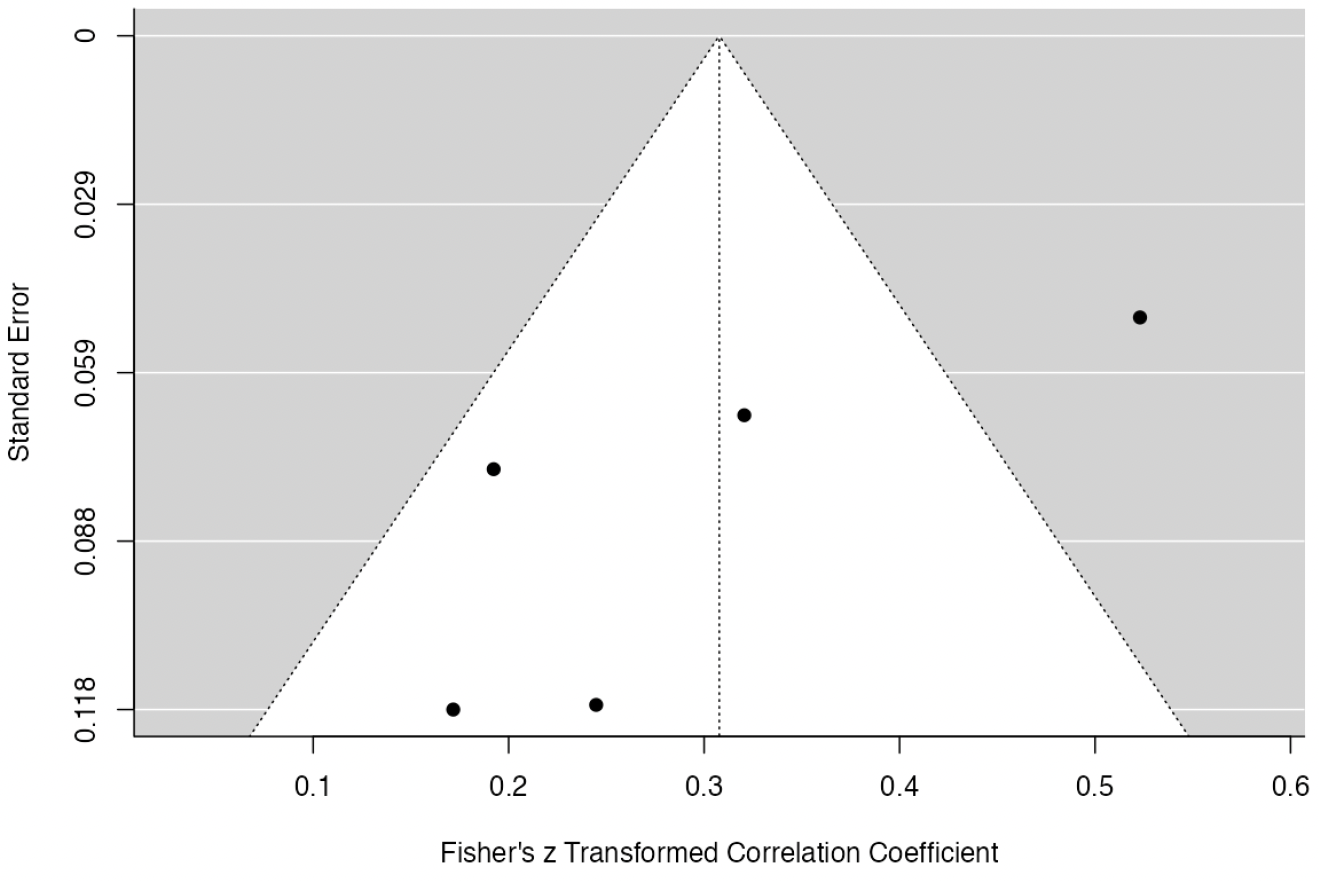
**

**S42. Forest Plot of Vulnerability to Harm in physical abuse victims
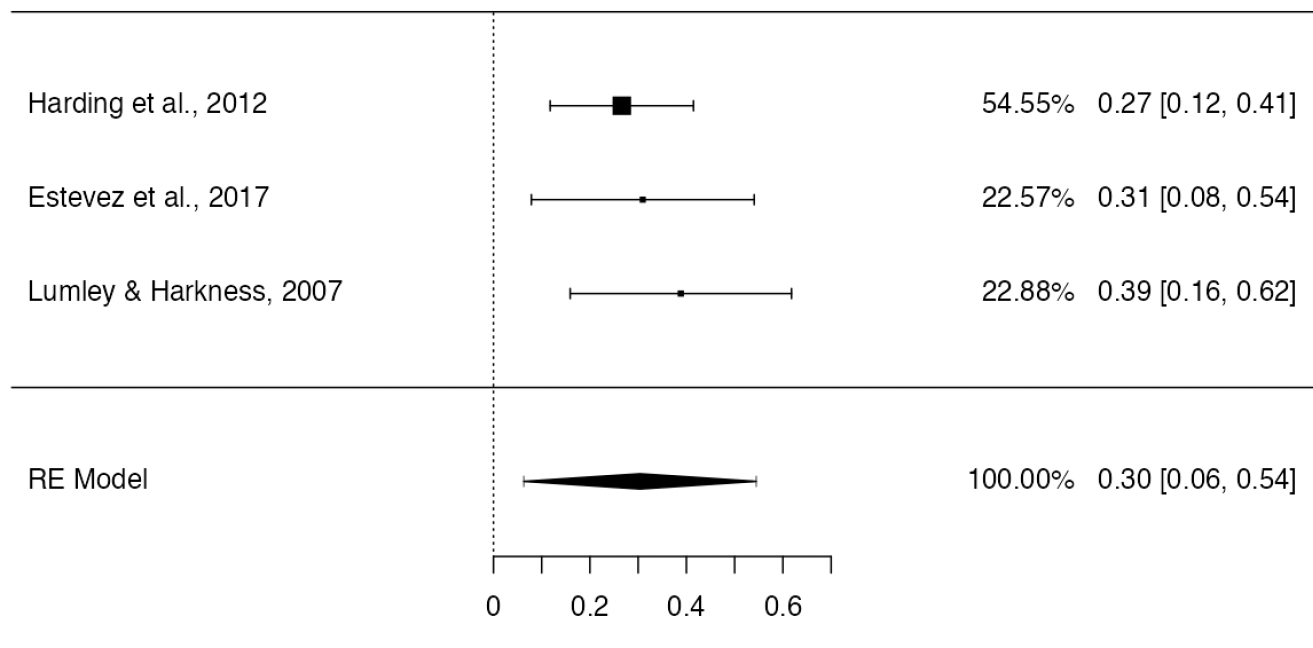
**

**S43. Funnel Plot of Vulnerability to Harm in physical abuse victims
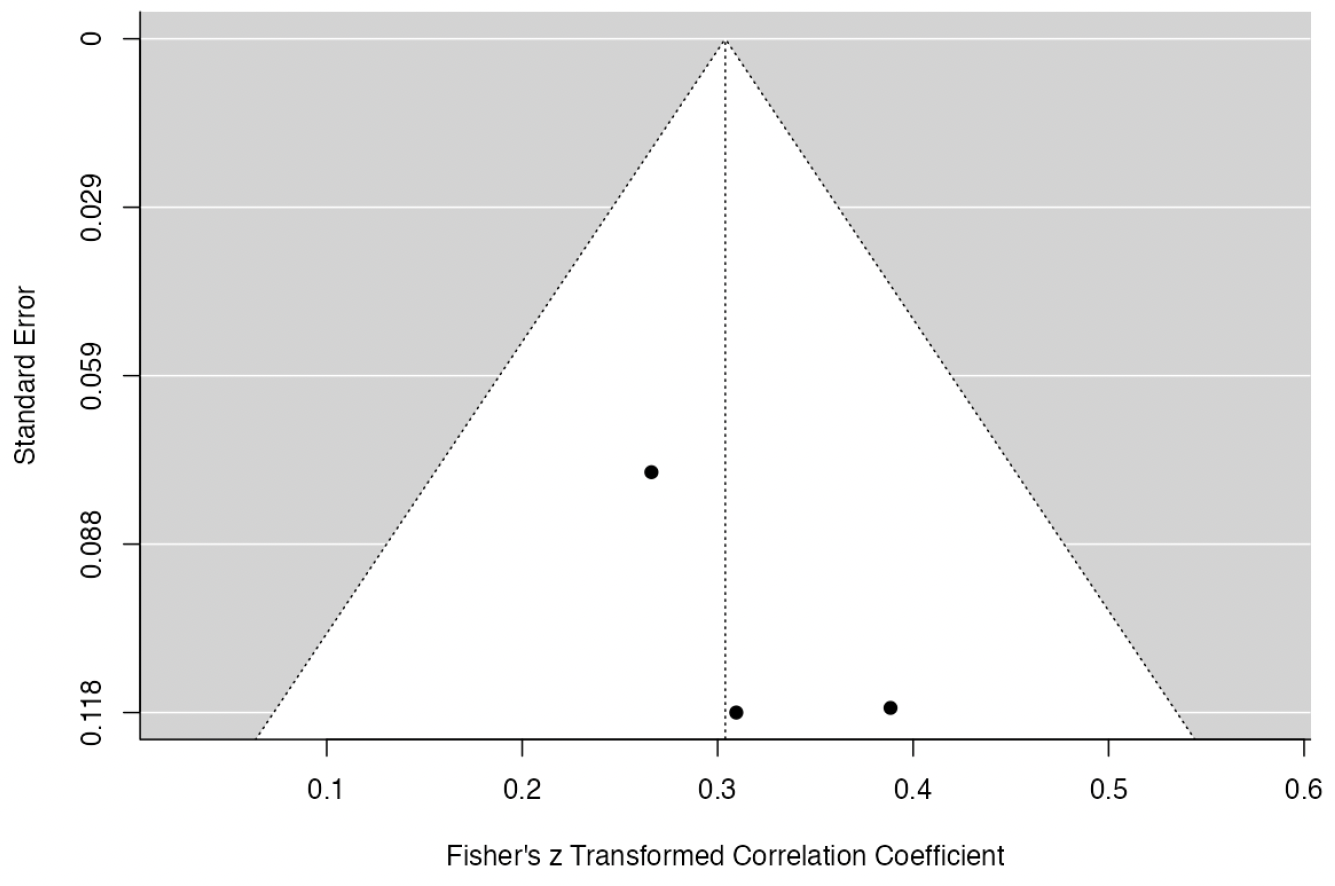
**

**S44. The correlation coefficient of the schema for victims of sexual abuse**

| **EMS** | **Correlation coefficient** | **Lower limit - Upper limit** | **t (p)** | **Q (d.f., p)** | **Tau²**  **(tau)** | **I²** | **Prediction interval** | **Begg and Mazumdar (p)** | **Egger’s regression (p)** | **Trim and Fill** |
| --- | --- | --- | --- | --- | --- | --- | --- | --- | --- | --- |
| Failure | 0.25 | 0.07- 0.44 | 4.31 (0.02) | 5.86 (3; 0.11) | 0.006 (0.08) | 49.87% | -0.06- 0.57 | 0.00 (1.00) | 0.39 (0.72) | 1 |
| Vulnerability to harm | 0.23 | -0.01- 0.47 | 4.18 (0.05) | 1.17 (2; 0.55) | 0 (0) | 0% | -0.01- 0.47 | 0.33 (1.00) | 0.80 (0.56) | 0 |
| Dependence | 0.23 | 0.09- 0.37 | 5.33 (0.01) | 3.64 (3; 0.30) | 0.001 (0.03) | 17.49% | 0.04- 0.41 | -0.66 (0.33) | -0.52 (0.65) | 1 |
| Abuse | 0.22 | 0.09- 0.36 | 4.74 (0.009) | 8.02 (4; 0.09) | 0.005 (0.07) | 50.15% | -0.01- 0.46 | 0.20 (0.81) | 0.88 (0.44) | 0 |
| Social isolation | 0.21 | 0.12- 0.30 | 6.62 (0.003) | 0.99 (4; 0.91) | 0 (0) | 0% | 0.12- 0.30 | 0.20 (0.81) | -0.09 (0.93) | 0 |
| Emotional deprivation | 0.19 | 0.12- 0.26 | 7.22 (<.001) | 2.66 (5; 0.75) | 0 (0) | 0% | 0.12- 0.26 | -0.06 (1.00) | 0.33 (0.75) | 1 |
| Abandonment | 0.16 | 0.09- 0.23 | 6.15 (0.002) | 1.38 (5; 0.92) | 0 (0) | 0% | 0.09- 0.23 | 0.06 (1.00) | 0.04 (0.96) | 2 |
| Self-sacrifice | 0.16 | 0.04- 0.29 | 4.12 (0.02) | 3.10 (3; 0.37) | 0.001 (0.03) | 17.77% | -0.007- 0.33 | 0.33 (0.75) | 1.40 (0.29) | 2 |
| Emotional inhibition | 0.16 | 0.04- 0.27 | 4.20 (0.02) | 0.10 (3; 0.99) | 0 (0) | 0% | 0.04- 0.27 | -0.66 (0.33) | -0.30 (0.79) | 1 |
| Defectiveness | 0.15 | 0.07- 0.23 | 5.31 (0.006) | 1.15 (4; 0.88) | 0 (0) | 0% | 0.07- 0.23 | 0.60 (0.23) | 0.80 (0.47) | 3 |
| Enmeshment | 0.12 | -0.12- 0.36 | 2.16 (0.16) | 0.47 (2; 0.79) | 0 (0) | 0% | -0.12- 0.36 | 1.00 (0.33) | 0.41 (0.75) | 0 |
| Unrelating standards | 0.12 | 0.01- 0.24 | 3.37 (0.04) | 1.39 (3; 0.70) | 0 (0) | 0% | 0.01- 0.24 | 0.33 (0.75) | 0.16 (0.88) | 0 |
| Subjugation | 0.08 | -0.02- 0.19 | 2.62 (0.07) | 0.42 (3; 0.93) | 0 (0) | 0% | -0.02- 0.19 | 0.33 (0.75) | 0.58 (0.61) | 2 |

**S45. Forest Plot of subjugation in IPV victims
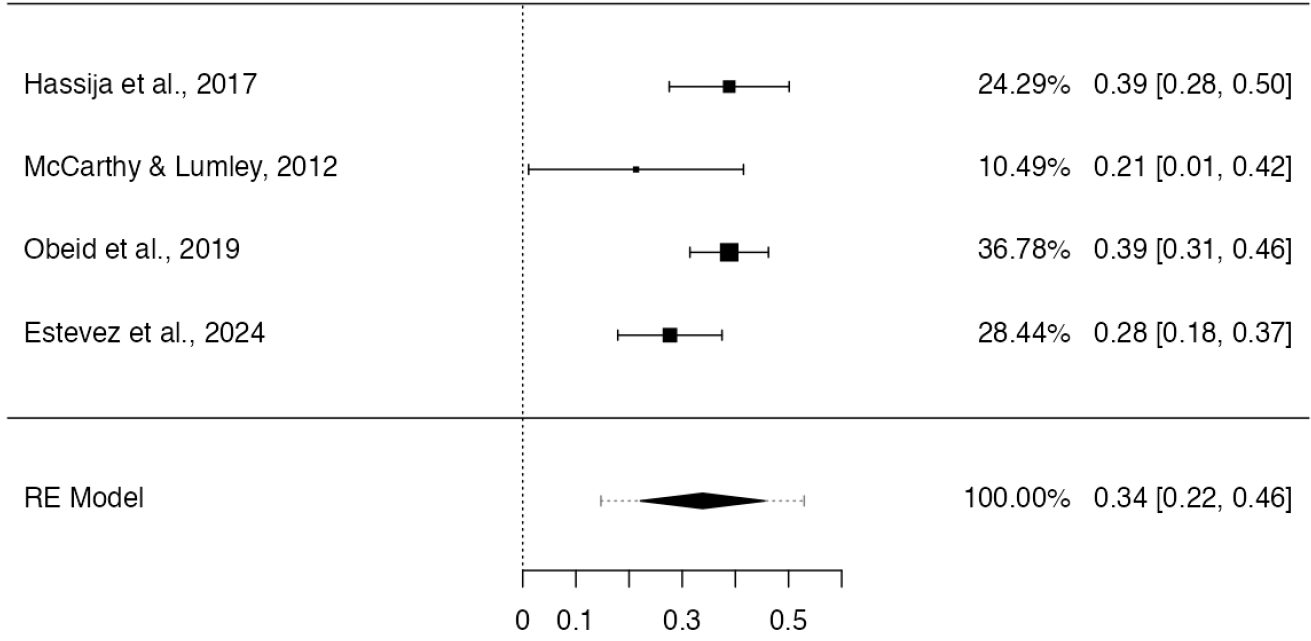
**

**S46. Funnel Plot of subjugation in IPV victims**

**
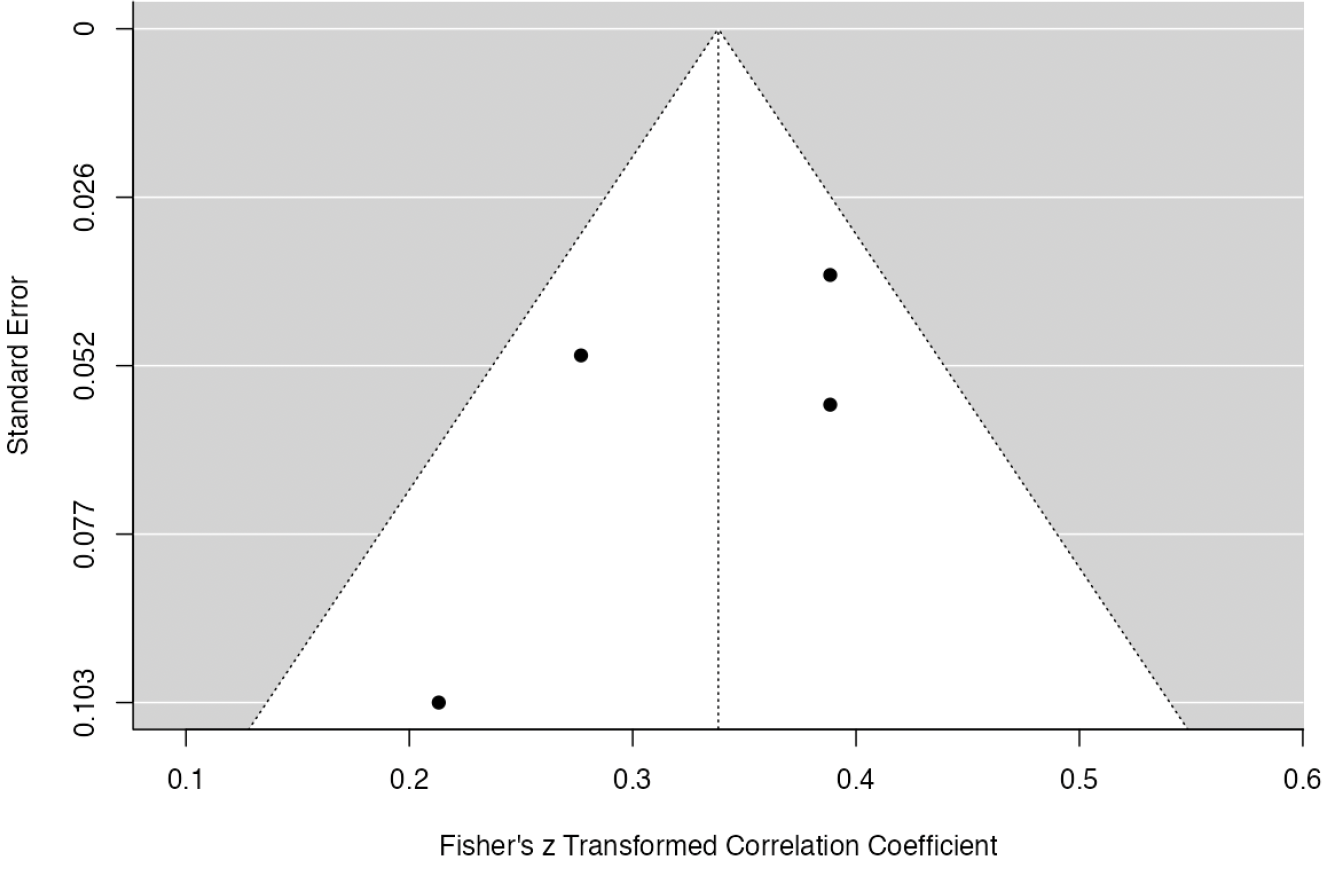
**

**S47. Forest plot of emotional deprivation in IPV victims
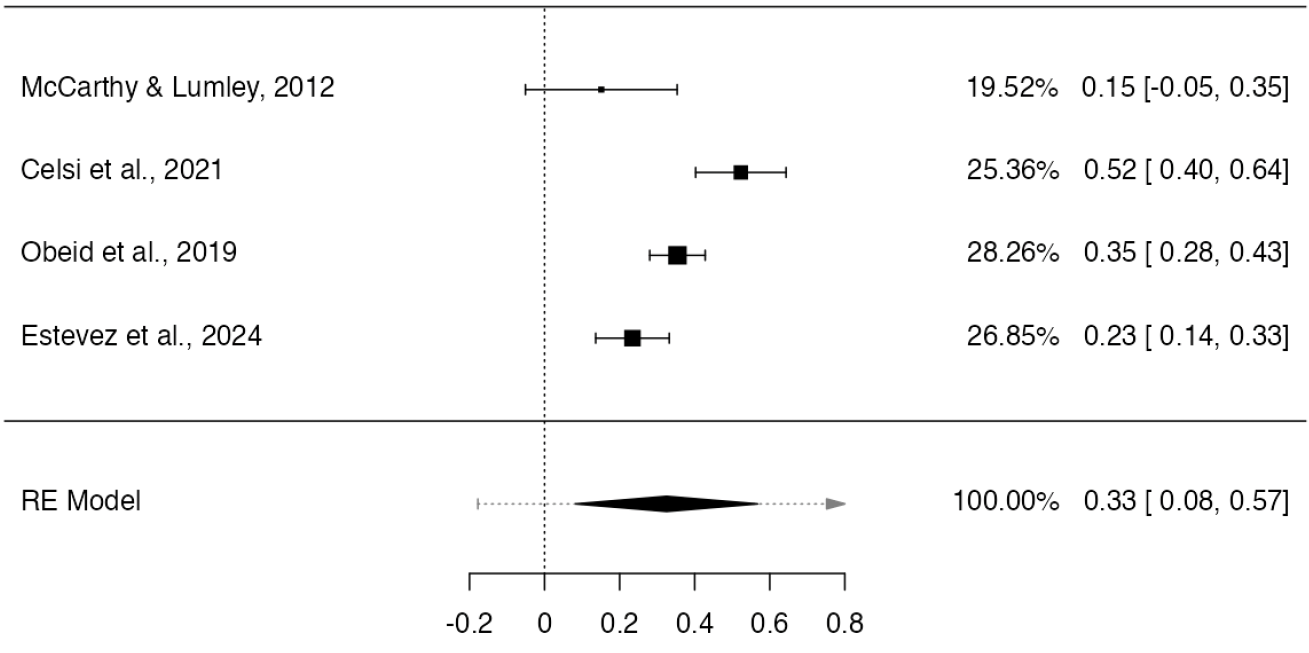
**

**S48. Funnel plot of emotional deprivation in IPV victims
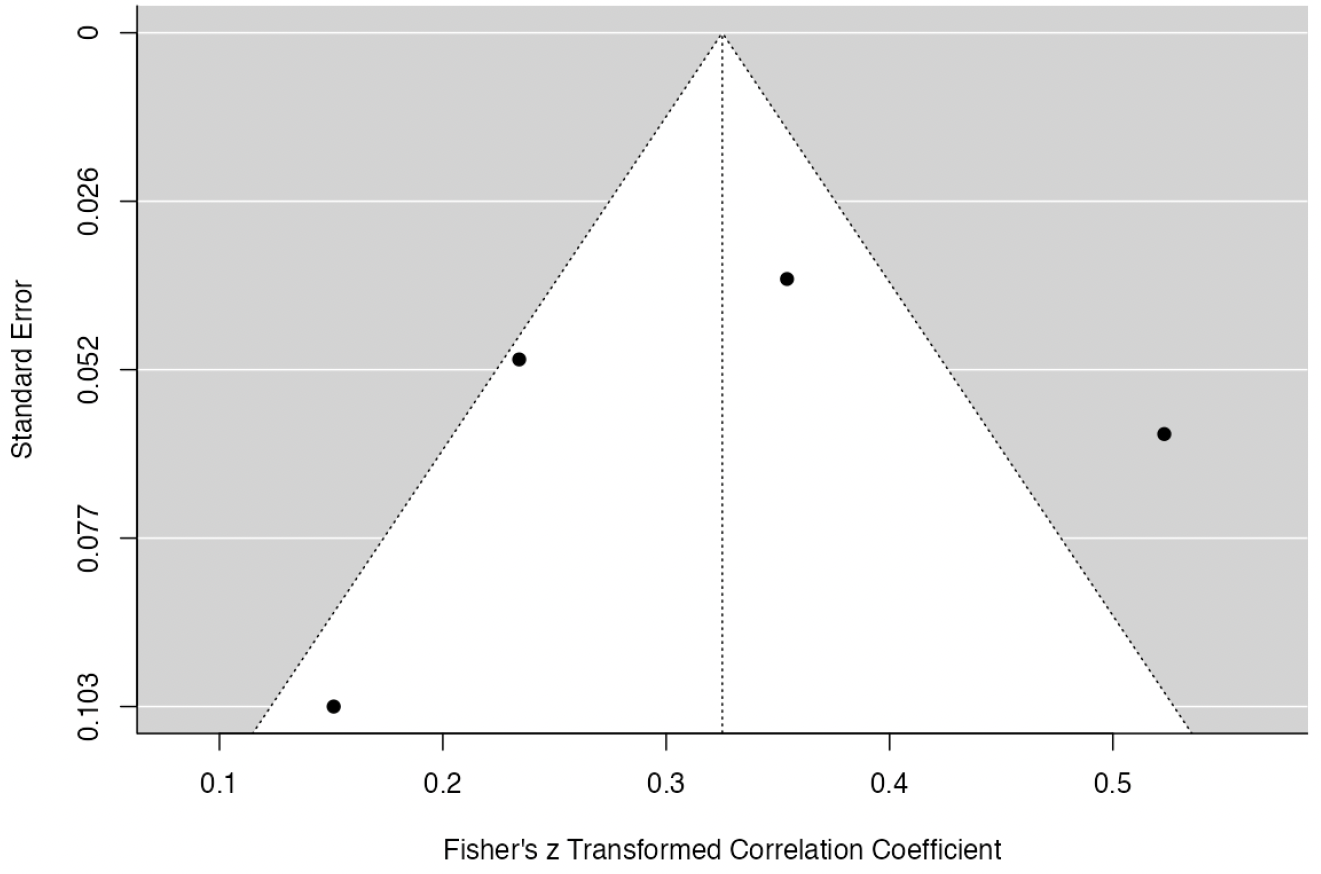
**

**S49. Forest plot of abuse in IPV victims
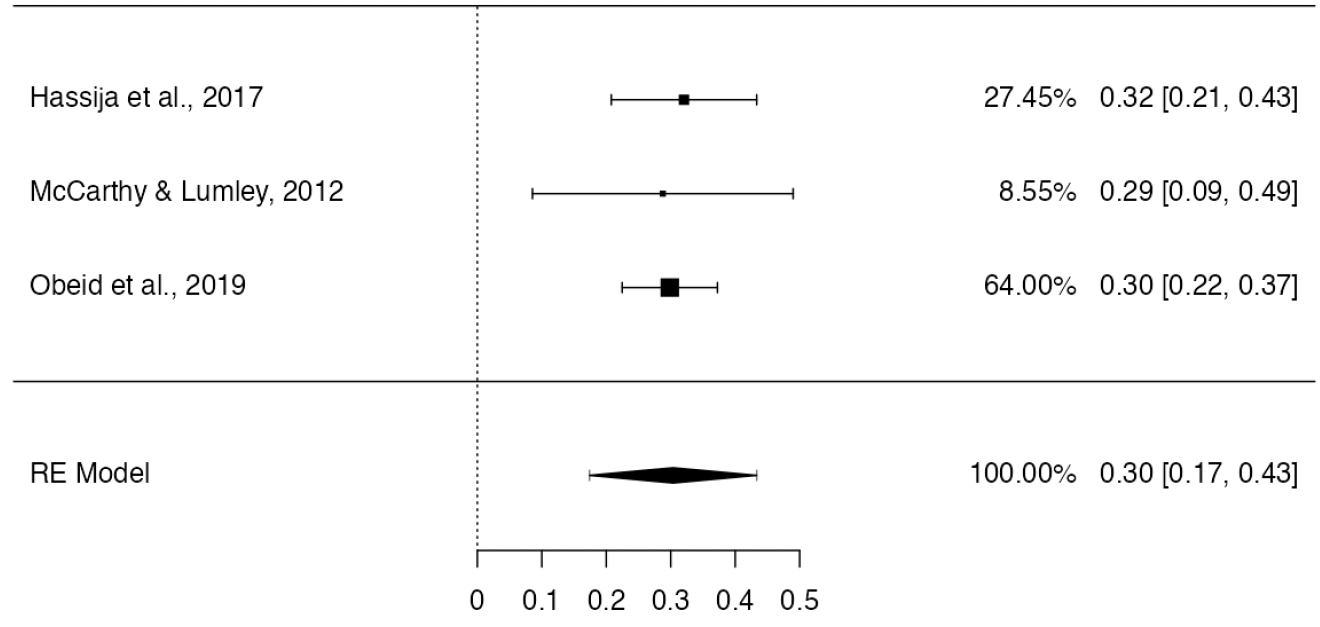
**

**S50. Funnel plot of abuse in IPV victims
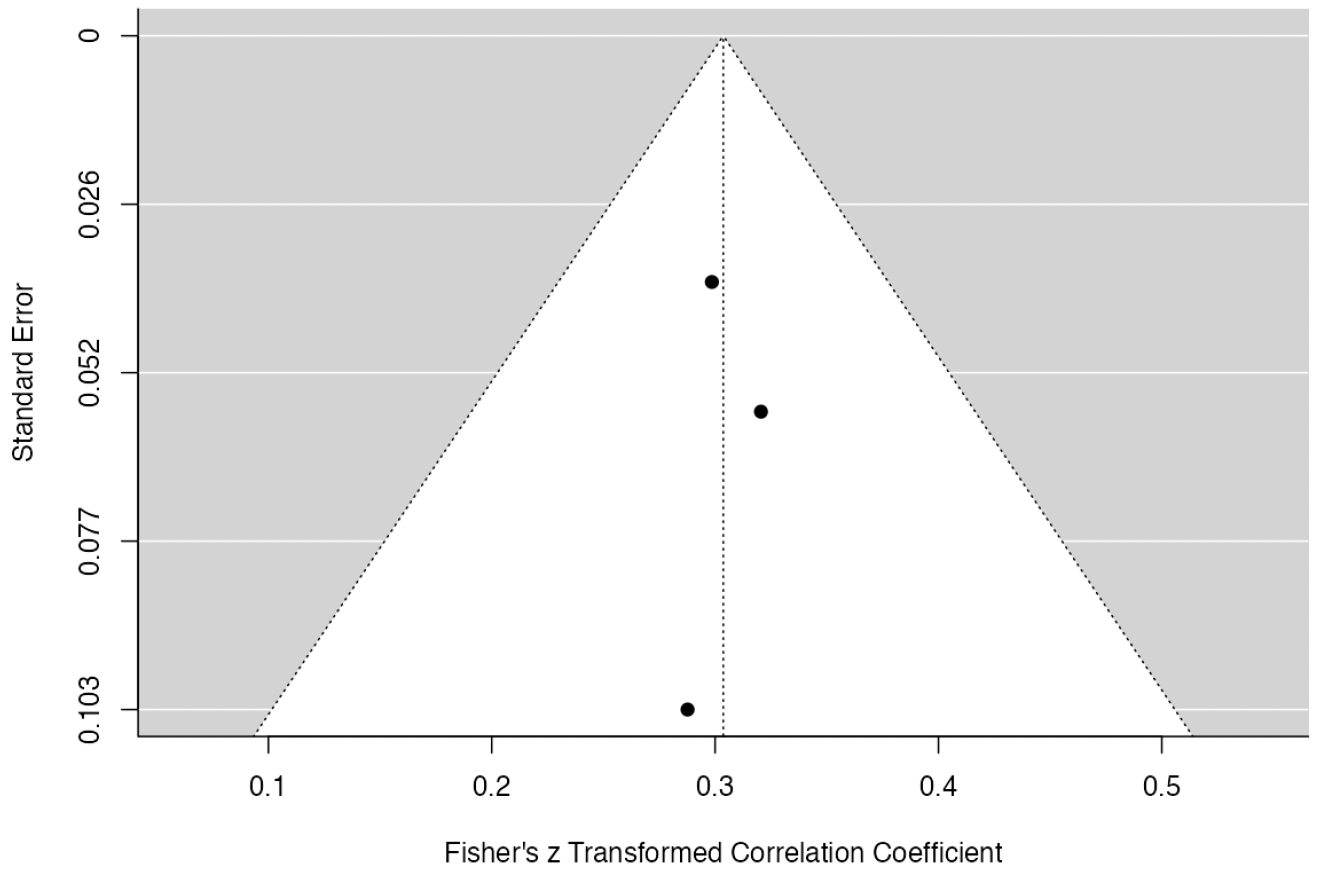
**

**S51. Forest plot of social isolation in IPV victims
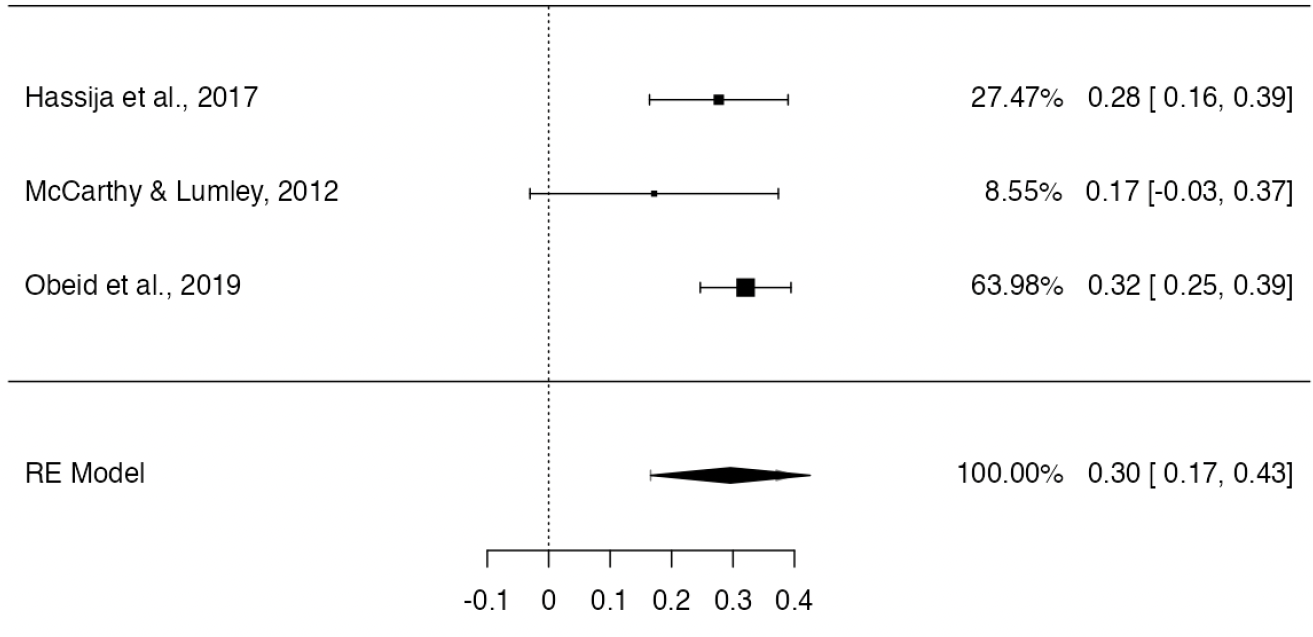
**

**S52. Funnel plot of social isolation in IPV victims**

**
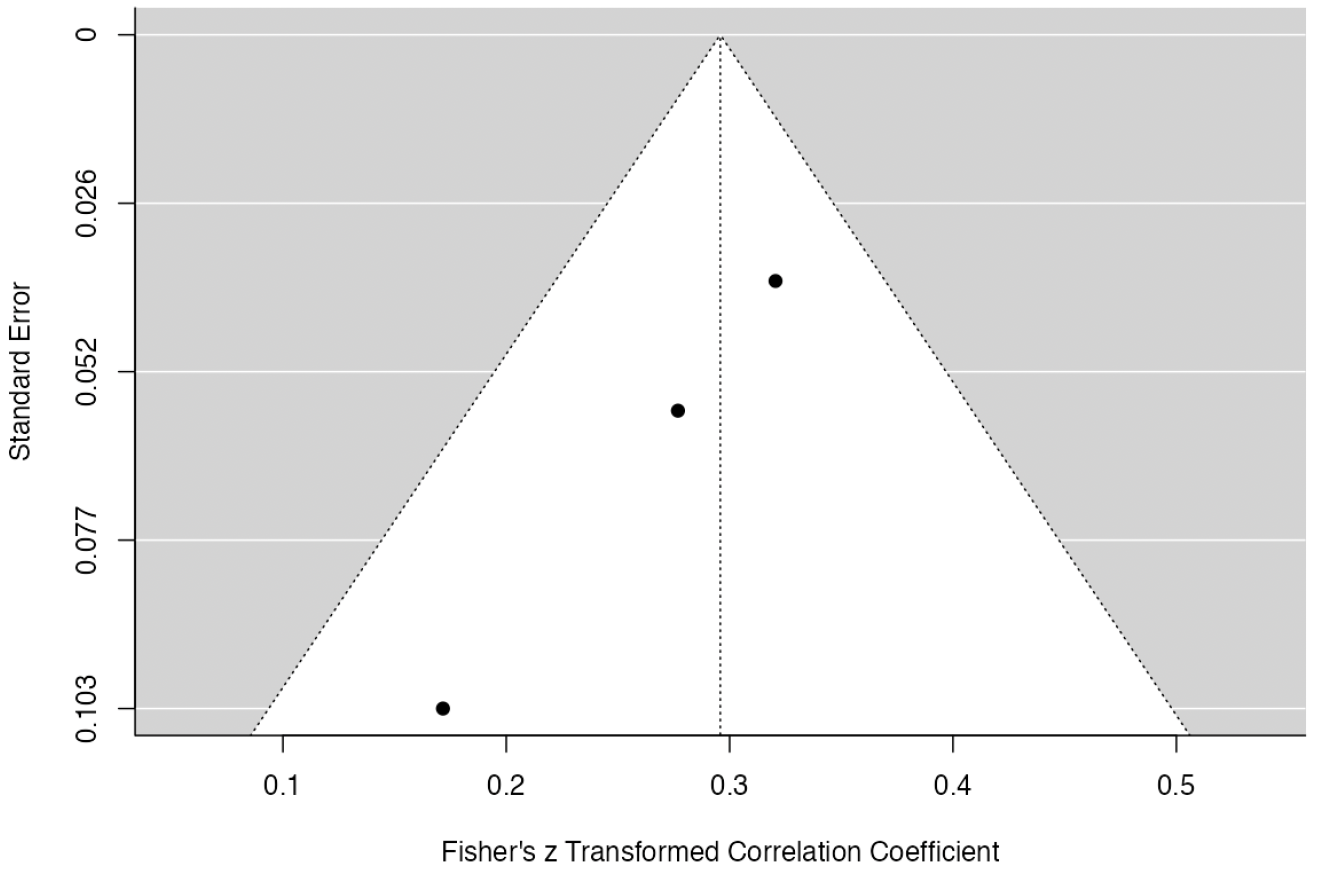
**

1. Department of Medical Science, Surgery, and Neurosciences, University of Siena, Siena, Italy

   *Corresponding Author, e-mail: [allison.uvelli@unisi.it](mailto:allison.uvelli@unisi.it) [↑](#footnote-ref-1)
2. School of Cognitive Psychotherapy (SPC), Grosseto, Italy [↑](#footnote-ref-2)
3. School of Cognitive Psychotherapy (SPC), Roma, Italy [↑](#footnote-ref-3)
